# Design and Synthesis of Actin-Targeting 10-Phenoxy Cytochalasan Analogues: Balancing Cytotoxicity and Migrastatic Activity

**DOI:** 10.1021/acsmedchemlett.5c00629

**Published:** 2026-01-09

**Authors:** Žaneta Javorská, Tereza Volfová, Johan Faivre, Wim Dehaen, Silvie Rimpelová, Magdaléna Labíková, Daniel Rösel, Jan Brábek, Pavla Perlíková

**Affiliations:** † Department of Organic Chemistry, Faculty of Chemical Technology, 52735 University of Chemistry and Technology Prague, Technická 5, 166 28 Prague, Czechia; ‡ Department of Cell Biology, BIOCEV, Faculty of Science, Charles University, Průmyslová 595, 252 50 Vestec, Prague West Czechia; 3 Department of Informatics and Chemistry, Faculty of Chemical Technology, University of Chemistry and Technology Prague, Technická 5, 166 28 Prague, Czechia; 4 Department of Biochemistry and Microbiology, Faculty of Food and Biochemical Technology, University of Chemistry and Technology Prague, Technická 5, 166 28 Prague, Czechia

**Keywords:** Cytochalasan, actin polymerization inhibitors, migrastatics, cancer cell invasiveness, cytotoxicity

## Abstract

Cytochalasans are actin polymerization inhibitors with potent migrastatic activity, but their potential therapeutic use is limited by their cytotoxicity. Here, we describe a modular late-stage approach that introduces unprecedented 10-phenoxy substituents into the cytochalasan scaffold via a Mitsunobu reaction. A series of ten 10-phenoxycytochalasan analogues was synthesized and evaluated for actin polymerization inhibition, migrastatic activity, and cytotoxicity (BLM, MRC-5, and HaCaT). At 10 μM concentration, several 7-hydroxy-10-phenoxycytochalasans (**12a,d**–**g**) significantly inhibited actin polymerization in vitro and showed migrastatic effects in a spheroid invasion assay. Para-substituents of the phenoxy group modulated cytotoxicity without compromising actin polymerization inhibition or migrastatic activity. In contrast, lipophilic ortho-substituents predicted by molecular docking to enhance actin binding failed to manifest migrastatic activity, underscoring the limitations of the molecular docking with this type of compounds. These findings demonstrate that migrastatic and cytotoxic effects can be decoupled in cytochalasan analogues and highlight 10-phenoxy substitution as a promising strategy toward noncytotoxic migrastatic agents.

## Graphical abstract

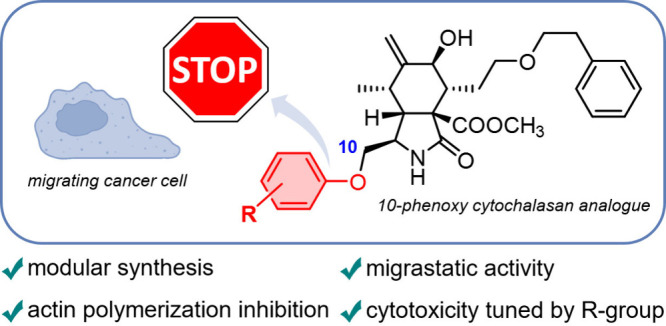

Actin polymerization is a critical process in the dynamics of the cytoskeleton and is indispensable for cell motility. The active migration and invasiveness of tumor cells are pivotal factors in the formation of metastases, which significantly worsen the prognosis of cancer patients. A considerable proportion of cancer-related deaths is attributable to metastatic disease;^1^ however, current cancer therapies do not specifically target the cellular processes underlying invasiveness and metastasis. The development of novel compounds that can effectively limit these processes, known as migrastatics,^2,3^ represents a promising new direction in cancer treatment by interfering with actin polymerization and related mechanisms of cell motility.

Cytochalasans are a class of natural compounds that inhibit actin polymerization by binding to the barbed ends of growing actin filaments (Figure 1).^4^ Among them, cytochalasin D (CytD, **1**) is a particularly potent inhibitor^5^ and is widely employed as a tool for investigating actin cytoskeleton dynamics. Cytochalasin B (CytB, **2**) exerts a similar, although less pronounced, effect.^6^ Both compounds display migrastatic activity,^7–10^ and the effects have been confirmed in vivo,^11,12^ highlighting the potential of cytochalasans as scaffolds for the development of migrastatic agents. A key limitation, however, is their relatively high cytotoxicity. While toxic effects typically manifest at concentrations higher than those required for migrastatic activity, strategies to minimize cytotoxicity are critical for advancing cytochalasans as migrastatics. One such approach involves the synthesis of prodrugs, which are selectively unmasked in tumor cells, as exemplified by the recently discovered prodrug of CytB (**2**).^13^ Another strategy involves modifying the structure of cytochalasan analogs based on structure–activity relationship (SAR).

**Figure 1:**
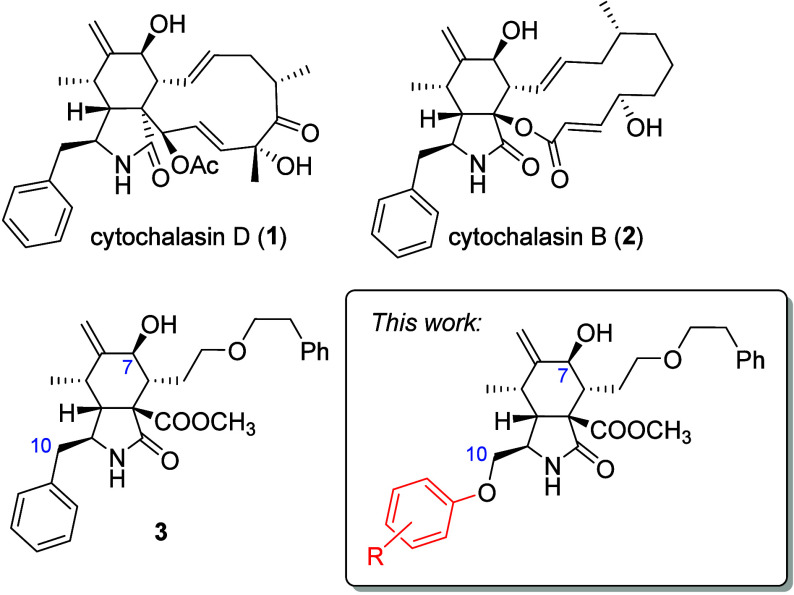
Natural cytochalasans and their synthetic analogues.

Although over 400 cytochalasans have been isolated from various fungi,^14^ the structural complexity and challenging syntheses of these compounds have limited comprehensive SAR studies within this compound class. To overcome these limitations, our group undertook a systematic investigation of cytochalasan scaffold modifications. We previously demonstrated that compound **3** (Figure 1), which lacks the complex macrocyclic moiety typical for most natural cytochalasans and contains a simple phenyl-substituted aliphatic side chain instead, retains migrastatic activity, albeit lower than that of CytD (**1**) or CytB (**2**).^15^ Notably, compound **3** exhibited no cytotoxicity at concentrations of up to 50 μM, providing the first evidence that migrastatic activity and cytotoxicity are less tightly correlated than previously assumed. We showed that migrastatic activity is modulated by the substitution pattern of the perhydroisoindolone core, with the 7-hydroxy group and exocyclic double bond enhancing activity.

Substitutions at position 10 may profoundly influence the biological activity of cytochalasan analogues, as structural variability at this site is highly restricted among this class of compounds. In natural cytochalasans, substituents at position 10 are derived from lipophilic amino acids that serve as biosynthetic precursors.^16^ The most common are phenylalanine as a source of the phenyl group at position 10 of cytochalasins and tryptophan providing the 3-indolyl group in chaetoglobosins. Replacement of substituents or the introduction of new functional groups may affect the interaction with target proteins and consequently influence the biological activity of the corresponding novel analogues. So far, modifications at this position have only been achieved via semisynthetic approaches or mutasynthesis.^17–19^ Our group has developed a late-stage modification method to introduce non-natural aryl groups at position 10 using iron-mediated cross-coupling with Grignard reagents.^20^ However, due to the reactivity of Grignard reagents, this approach is limited in scope and does not enable modular synthesis of analogues containing the critical 7-hydroxy group. In this study, we employed nucleophilic substitution, which offers broader applicability for the introduction of phenoxy groups at position 10 that are not present in any known natural cytochalasan. The novel 10-phenoxy cytochalasan analogues were designed on the basis of both virtual screening and conventional SAR approaches, with a particular emphasis on actin polymerization inhibition and the migrastatic activity of the resulting compounds.

A library of virtual cytochalasans was constructed using a building block enumeration approach, replacing phenyl moiety in the 10-position of derivative **3** and conjugating the corresponding fragment with commercially available phenols, alcohols, thiophenols, and thiols. This resulted in a screening database of 76 294 compounds. These compounds were ranked using molecular docking to the known crystal structure of monomeric G-actin bound to CytD (**1**) (PDB: 3EKU).^21^ Inspection of the top ranked compounds showed the presence of many compounds derived from phenols with bulky lipophilic substituents, particularly in the *ortho*-position. These compounds were then prioritized for synthesis. During the preparation of this manuscript, a new crystal structure of CytD (**1**) bound to F-actin was released (PDB: 9L2N).^22^ The docking protocol used in the original virtual screen was repeated for the prioritized compounds to compare any differences in pose prediction between these two actin structures, obtaining similar poses and similar scores ().

The introduction of phenoxy groups can be achieved either through a Mitsunobu reaction of a cytochalasan-based alcohol with a phenol or through a nucleophilic substitution of a cytochalasan-based electrophile with the corresponding phenolate. Our initial selection of phenols to be reacted with the cytochalasan precursors was based on molecular docking results (favoring *ortho*-alkylated phenols) and also included phenols with electron-donating or withdrawing groups in the *para*- and *meta*-positions, typical for the Topliss batchwise scheme.^23^

The synthesis of the cytochalasan core was developed earlier in our group.^20^ Pyrrolidone **4** served as the starting material in a 9-step synthesis to obtain 10-hydroxycytochalasan analogue **6** via its silyl-protected intermediate **5** (Scheme 1). Alcohol **6** served as a precursor for subsequent modifications in position 10 leading to cytochalasans lacking a hydroxyl group on the six-membered ring (Scheme 1). To introduce phenoxy groups into the cytochalasan core, two methods were extensively studied: a nucleophilic substitution and a Mitsunobu reaction. The nucleophilic substitution between bromide **7**, provided by Appel reaction of alcohol **6** (81% yield), and a corresponding 2-*tert*-amylphenol at the presence of NaI and K_2_CO_3_ as a base were performed in acetonitrile at 70 °C. The *o*-*tert*-amylphenoxy derivative **8c** was obtained in a yield of 70%. The Mitsunobu reaction directly combined alcohol **6** with the corresponding phenols. The reaction was performed in the presence of PPh_3_ and di-*tert*-butyl azodicarboxylate (DtBAD) in THF at 120 °C for 1 h in a microwave reactor. Two phenoxy derivatives, **8a** and **8b**, were obtained in moderate yields of 52% and 19%, respectively. Although purification of **8a** required a preparative HPLC separation, and it was observed that the Mitsunobu reaction is highly sensitive to traces of water in the solvent, both the nucleophilic substitution and the Mitsunobu reaction effectively produced the desired products with comparable yields.

**Scheme 1:**
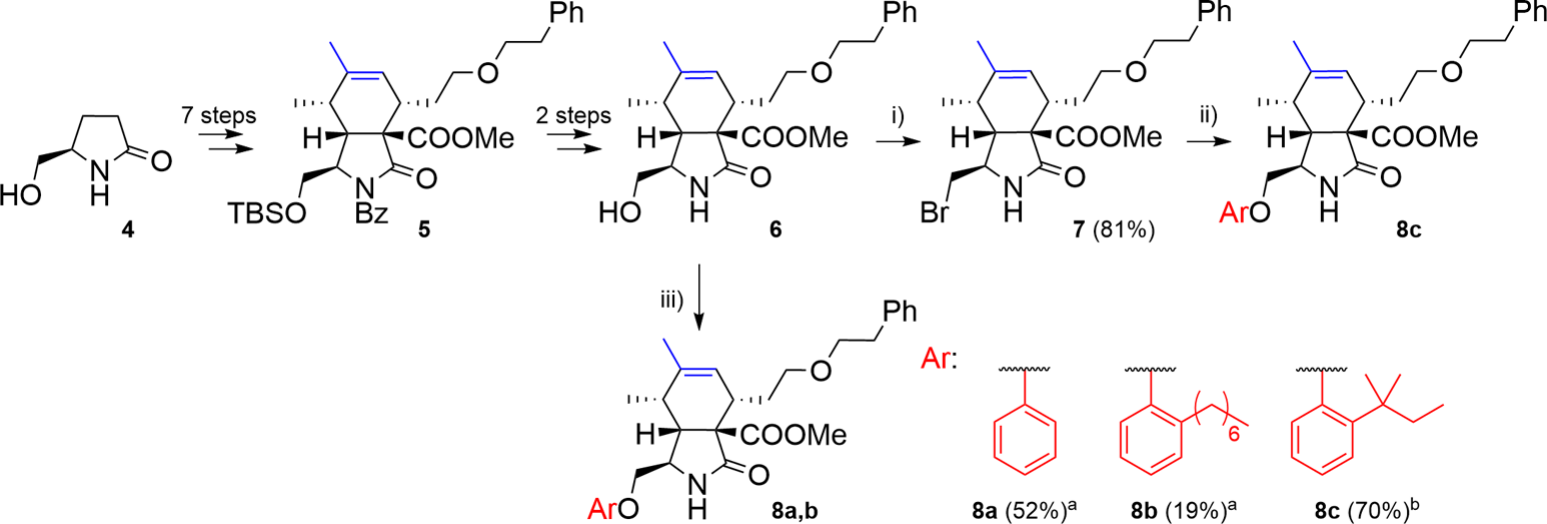
Reagents and Conditions: (i) CBr_4_, PPh_3_, DCM, 23 °C, 2 h; (ii) ArOH, K_2_CO_3_, NaI, CH_3_CN, 70 °C, 28 h; (iii) ArOH, PPh_3_, DtBAD, THF, 120 °C, 1 h, Microwave Irradiation

a Prepared by pathway (iii).
b Prepared by pathway (ii).

The 7-hydroxy group has previously been shown to be essential for the migrastatic activity of cytochalasan analogues lacking the macrocyclic moiety.^15^ Therefore, to obtain 10-phenoxy cytochalasan analogues with this substitution pattern, we first prepared a suitable intermediate (Scheme 2). The silyl-protected alcohol **5** was reacted with mCPBA in DCM to produce two diastereomeric epoxides, **9a** (74%) and **9b** (18%), in a ratio of approximately 4:1, which were separable using flash chromatography with an excess of silica gel (120 g of silica gel were used per 1 g of silyl-protected alcohol **5**). Subsequently, epoxide isomerization was carried out by generating a sterically hindered organoaluminum base in situ.^24^ First, LiTMP was formed from 2,2,6,6-tetramethylpiperidine (HTMP) and butyllithium, followed by reaction with Et_2_AlCl and epoxide **9a** in toluene. During the epoxide isomerization step, partial deprotection of the benzoyl group occurred; therefore, a small amount of sodium and methanol were directly added to the reaction mixture, resulting in full deprotection, and compound **10** was obtained in a yield of 54%. The stereochemistry of derivatives **9** and **10** was confirmed by 2D NMR spectra. The final step was the deprotection of the silyl group, performed by using 3HF·NEt_3_ in acetonitrile. Alcohol **11** was obtained in an excellent yield of 94%.

**Scheme 2:**
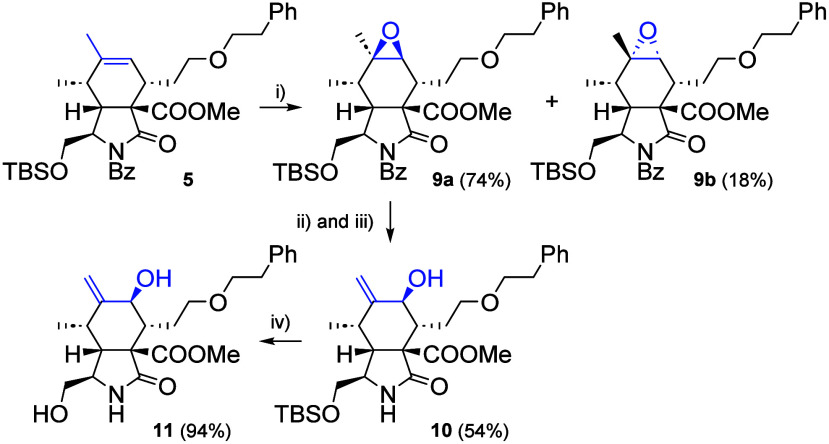
Reagents and Conditions: (i) mCPBA, DCM, 0 to 23 °C, 1.5 h; (ii) HTMP, BuLi, Et_2_AlCl, Toluene, 0 to 23 °C, 7.5 h; (iii) NaOMe, MeOH, 23 °C, 1 h; (iv) 3HF·NEt_3_, CH_3_CN, 23 °C, 20 h

With alcohol **11** in hand, we evaluated both the nucleophilic substitution and the Mitsunobu reaction for introducing a phenoxy group into the cytochalasan core. Unfortunately, we faced challenges in preparing the bromide required for the first approach. Following multiple optimizations of the reaction conditions for the Appel reaction, we managed to synthesize bromide **S1** (for details, see ); however, the results exhibited limited consistency and reproducibility, preventing us from obtaining a sufficient quantity of the material for the subsequent steps. Therefore, our focus shifted to the Mitsunobu conditions.

Using alcohol **11** and phenols in the presence of PPh_3_ and DtBAD in THF, we successfully obtained seven 10-phenoxy cytochalasan derivatives **12a–12g**, with moderate yields from 15% to 46% (Scheme 3). Although a series of final compounds was achieved, it is important to mention that there are some limitations. Typically, at least two side products were observed in the reaction mixture. Two specific ones (**S3** and **S4**) were fully characterized (see the ), while others were observed by NMR of the crude reaction mixture. As a result, all products required extensive purification. The reaction is also highly sensitive to moisture, which poses challenges in achieving consistent results. Despite these difficulties, we were able to acquire sufficient amounts of final products for subsequent biological testing.

**Scheme 3:**
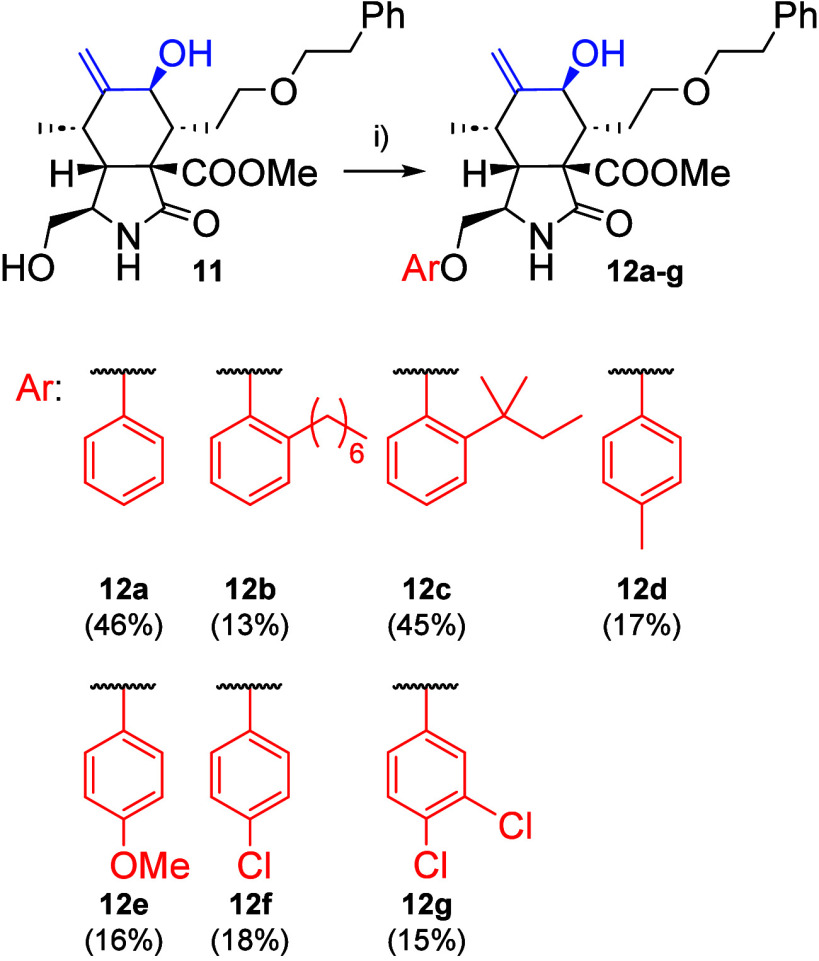
Reagents and Conditions: (i) ArOH, PPh_3_, DtBAD, THF, 90 or 120 °C, 0.5–4 h, with or without Microwave Irradiation

The 11 novel cytochalasan analogues were subjected to in vitro cytotoxicity screening (Table 1). Compounds **8a**–**8c**, which lack the 7-hydroxy group, and derivatives **12b**, **12c**, **12f**, and **12g** exhibited moderate cytotoxic activities at tens of micromolar concentrations in both cancerous (BLM, human melanoma) and noncancerous (MRC-5, human fibroblasts; HaCaT, human keratinocytes) cell lines after 72 h of treatment. Derivative **12e** exhibited modest cytotoxicity only against HaCaT cells, with an IC_50_ value of 35.84 μM. In contrast, alcohol **11**, which lacks the 10-phenoxy group, as well as derivatives **12a** and **12d**, did not exhibit cytotoxicity up to 50 μM (the highest one evaluated). Importantly, all novel compounds were considerably less cytotoxic than the natural products CytD (**1**) and CytB (**2**) in MRC-5 and HaCaT cells, while their effects on BLM cells were either slightly weaker or comparable.

**Table 1:** Cytotoxic Activity of Compounds **8a**–**8c**, **11**, and **12a**–**12g** in Human Melanoma Cells (BLM), Human Fibroblasts (MRC-5), and Human Keratinocytes (HaCaT) after 72 h of Treatment Determined by the WST-1 Assay^a^

	IC_50_ ^a^ ± SEM [μM]		
	BLM	MRC-5	HaCaT
CytD (**1**)	26.20 ± 0.55^b^	0.27 ± 0.01^b^	0.02 ± 0.01
CytB (**2**)	13.71 ± 1.20^b^	2.36 ± 0.07^b^	0.74 ± 0.08
**8a**	24.07 ± 0.96	25.42 ± 2.45	24.10 ± 2.43
**8b**	31.28 ± 0.78	18.01 ± 0.88	18.50 ± 1.73
**8c**	21.77 ± 1.58	14.68 ± 0.87	20.71 ± 1.37
**11**	>50	>50	>50
**12a**	>50	>50	>50
**12b**	19.35 ± 2.18	27.52 ± 1.02	11.74 ± 0.51
**12c**	17.54 ± 1.75	18.53 ± 2.19	29.54 ± 2.78
**12d**	>50	>50	>50
**12e**	>50	>50	35.84 ± 1.13
**12f**	33.63 ± 3.28	36.47 ± 1.01	39.11 ± 0.82
**12g**	23.92 ± 1.83	30.05 ± 3.72	28.99 ± 0.94

a The IC_50_ value represents the half-maximum inhibitory concentration.

b The values represent the mean that originates from at least three independent experiments; SEM – the standard error of the mean.

c Values taken from ref 15.

We next evaluated the ability of the novel cytochalasan analogues to inhibit actin polymerization (Figure 2A, ) and their migrastatic activity (Figure 2B, ), using CytD (**1**) as a positive control. Both assays were conducted at a single concentration of 10 μM. Compounds **8a** and **8c** displayed only weak inhibition of actin polymerization *in vitro*, and the effect of **8b** was not significantly different from that of the negative control (DMSO). In contrast, the phenoxy cytochalasan analogues bearing a 7-hydroxy group (**12a**–**g**) exhibited pronounced inhibition of actin polymerization, although they were less potent than CytD (**1**). All compounds were further evaluated in a spheroid invasion assay using BLM cells (Figure 2B, ). The strongest migrastatic effects were observed for compounds **12a** and **12d**–**g**. The importance of the phenoxy substituent at position 10 for biological activity was underscored by 10-hydroxy derivative **11**, which showed neither inhibition of actin polymerization nor migrastatic effects.

**Figure 2:**
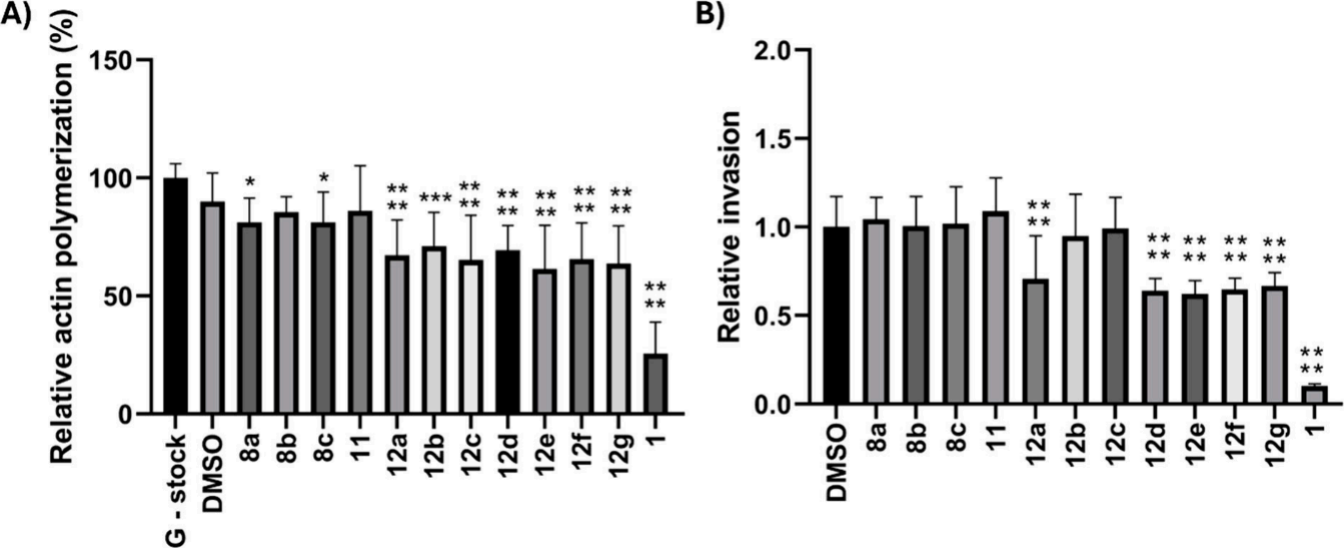
Actin polymerization assay (A) at a 10 μM concentration of evaluated compounds. Spheroid invasion assay of BLM cells (B) treated with 10 μM concentration of the tested compounds for 48 h. The error bars represent standard errors of the mean. The compounds were measured in several batches, and therefore, the error bars for individual control experiments (G-stock, DMSO) within different batches may vary from those shown in the figure. The *p*-values always refer to the control experiments performed within the corresponding batch. *p ≤ 0.05; ***p ≤ 0.001; ****p ≤ 0.0001 (one-way ANOVA).

To further investigate the cellular effects of these compounds, derivative **12e**, which showed inhibition of actin polymerization and migrastatic activity and did not exhibit cytotoxicity up to 50 μM concentration in both BLM and MRC-5 cells and only weak cytotoxicity in HaCaT, was selected for microscopic analysis of F-actin dynamics in living U-2 OS cells (Figure 3; for video files, see ). Time-lapse microscopy over a 60 min period revealed that treatment with **12e** at 10 μM concentration did not cause any apparent alterations in actin microfilament organization. However, at a concentration of 50 μM, a significant reduction in membrane ruffling and lamellipodia formation was observed. This observation confirms that compound **12e** acts as an inhibitor of actin polymerization.

**Figure 3:**
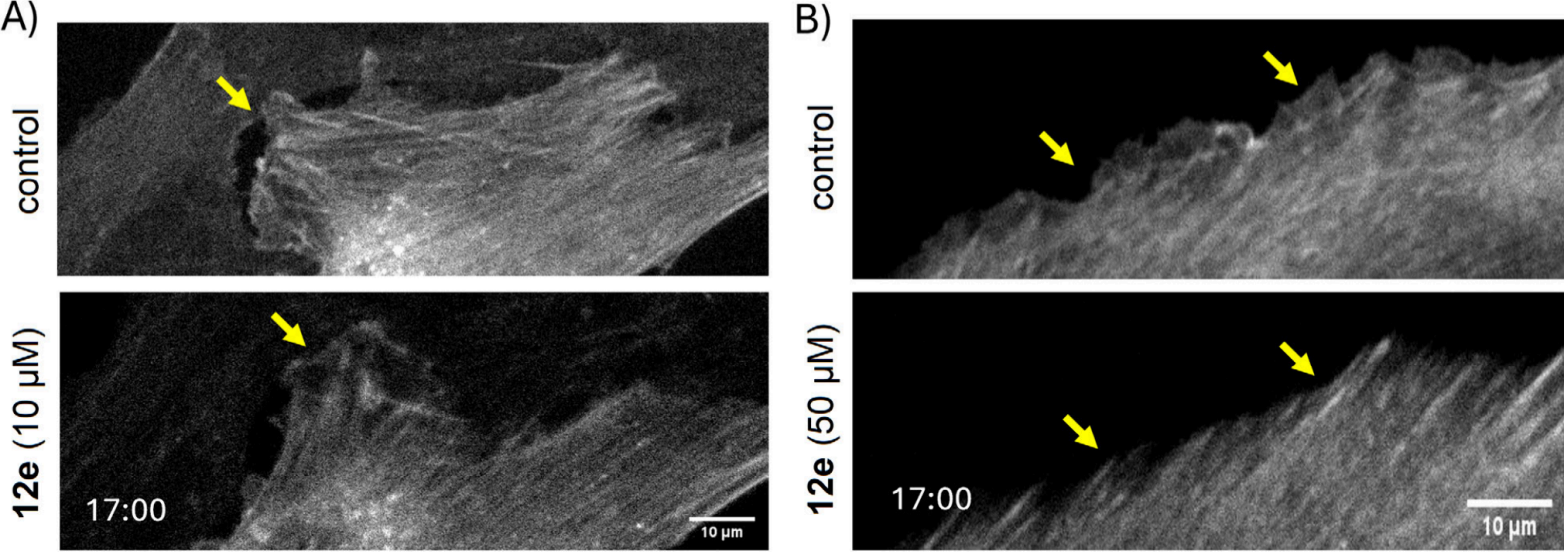
Representative fluorescent images from time-lapse imaging of the actin cytoskeleton in untreated cells (control) and cells treated with compound **12e** at different concentrations. Images of U-2 OS cells stably expressing LifeAct-tdTomato were obtained from video recordings (for video files, see ) using 10 μM (A) and 50 μM (B) concentrations of **12e**. The upper images show untreated cells (control), and the bottom images show the same cells after 17 min of treatment at the given concentration. U-2 OS cells were imaged in μ-Slide 8-well ibiTreat chambers using a Leica DMi8 SP2 microscope (40×/0.6 dry objective). The arrowheads indicate changes in the membrane ruffling. Scale bar: 10 μm.

Neither of the two compounds prioritized by virtual screenings **12b** and **12c** showed activity in the spheroid cell migration assay. However, both compounds inhibited actin polymerization in vitro and were cytotoxic. The expected activity of both compounds was mainly rationalized by shape and the lipophilic effect. In the predicted pose, the bulky lipophilic substituents of the phenoxy groups in compounds **12b** and **12c** are close to several lipophilic amino acids such as TYR133, TYR169 and MET355. Finally, in the hydrophobic cleft region of actin, at which the 10-phenyl group of CytD (**1**) is found, other known binders such as jaspisamide A (PDB:1QZ6)^25^ and reidispongiolide (PDB:2ASP)^26^ seem to engage via lipophilic interactions using the tail segment of these molecules. Therefore, it was surprising that the lipophilic substitution pattern identified in the in silico hits failed to confer migrastatic activity, even though the observed inhibition of actin polymerization indicated successful direct actin engagement without the corresponding migrastatic activity. In addition to the generally known limitations of molecular docking,^27,28^ we attribute the ambivalent results of docking to overestimation of lipophilic effects by the scoring method. We also did not account for solvent exposure: The region where the lipophilic tail is expected to bind is not deeply buried and is partially solvent-exposed, particularly for larger **12b**. As the region around the phenyl group in the 10-position of CytD (**1**) is relatively different in the newly released F-actin structure (PDB:9L2N), redocking was attempted but this resulted in a structure that was scored highly and had good agreement with the originally obtained pose.

10-Phenoxycytochalasan analogues **8** and **12** exhibit intriguing correlations among cytotoxicity, inhibition of actin polymerization, and migrastatic activity. The 7-hydroxy group is crucial for the migrastatic effect and significantly enhances inhibition of actin polymerization. Compounds lacking this substituent (**8a**-**c**) inhibited actin polymerization only weakly or not at all and did not display migrastatic activity. However, they showed cytotoxicity against all three cell lines used in this study. These findings indicate that the cytotoxic effect in this case is most likely mediated by a different mechanism than actin polymerization inhibition and that the compounds probably interact with additional, yet unidentified, protein targets.

All 10-phenoxycytochalasan analogues bearing a 7-hydroxy group (**12a**–**12g**) proved to be similarly potent inhibitors of actin polymerization in vitro. Interestingly, further substitution of the 10-phenoxy group modulates the cytotoxicity and migrastatic activity of the resulting compounds. The unsubstituted derivative **12a** and those carrying electron-donating substituents in the *para*-position (**12d**,**12e**), were generally not cytotoxic, with the exception of compound **12e** that exhibited modest cytotoxic effect only against HaCaT cells (IC_50_ of 35.84 μM) whereas electron-withdrawing substituents markedly increased cytotoxicity (**12f**,**12g**). Actin polymerization is important not only for cell motility but also for other cellular processes, and therefore, disruption of actin cytoskeleton is commonly associated with cytotoxic effects. However, our data show that for 10-phenoxycytochalasans **12** cytotoxicity does not correlate with inhibition of actin polymerization. This observation further supports the hypothesis that the cytotoxicity of cytochalasans is not always directly linked to inhibition of actin polymerization and may, at least in part, arise from interactions with other proteins. For example, CytB (**2**) inhibits the glucose transporter GLUT1.^29^ In accordance with this hypothesis, the inhibition of actin polymerization and migrastatic activities remains unchanged within the 7-hydroxy-10-phenoxy cytochalasan series **12** while cytotoxicity is affected by the electronic properties of the phenoxy group. Elucidation of the exact protein targets involved will be addressed in future studies.

The most remarkable finding concerns compounds **12b** and **12c**, which, although capable of inhibiting actin polymerization to the same extent as other 7-hydroxy-10-phenoxycytochalasans, did not show any migrastatic effect. Impaired cellular permeability is an unlikely explanation, since these compounds displayed the strongest cytotoxic activity among all 7-hydroxy-10-phenoxy cytochalasans. A hypothesis to explain the lack of migrastatic activity in cells is competition between binding of these derivatives to actin and another, yet unidentified protein associated with the cytotoxic effect or an uneven intracellular distribution of the compounds.

Our results demonstrate that in cytochalasan analogues lacking the macrocyclic ring cytotoxicity can be tuned by introducing *para*-substituents on the 10-phenoxy group, where electronic effects dictate the activity. At the same time, activity observed in the actin polymerization assay is not necessarily predictive of migrastatic potential.

A recent study describing the interference of CytB (**2**) with actin-binding proteins suggests that the impact of cytochalasans on the actin cytoskeleton is not limited to direct interactions with actin only.^30^ Consistently, our results indicate that novel cytochalasan derivatives might interact not only with actin but also with additional protein targets that remain to be identified.

In conclusion, we developed a new method for the modification of compounds bearing a cytochalasan core. Introduction of a 10-phenoxy group, which does not naturally occur in cytochalasan structures, in the final step of the synthesis enables a modular approach to generate a series of analogues with diverse biological activities. We demonstrated that the presence of a 7-hydroxy group on the cytochalasan core is essential for migrastatic activity, while cytotoxicity can be modulated via electronic effects of *para*-substituents on the phenoxy group.

Unexpectedly, compounds designed by virtual screening that carried bulky lipophilic alkyl substituents in the *ortho*-position of the 10-phenoxy group (**12b**,**12c**) did not exhibit migrastatic activity despite effectively inhibiting actin polymerization. These findings suggest that cytochalasan analogues might interact with cellular targets beyond actin, contributing to cytotoxic effects. Importantly, the relationship between actin inhibition, migrastatic activity, and cytotoxicity is complex, therefore, further studies are required to elucidate the precise mechanisms of action.

The ability to decouple the cytotoxicity from migrastatic activity represents a key advance that may guide the future design of novel derivatives suitable as lead structures for the development of noncytotoxic migrastatic agents.

## Supplementary Material





## Notes

Supplementary videos (fluorescence microscopy of the actin cytoskeleton) (ZIP)
Methods (molecular docking, cytotoxicity assay, actin polymerization assay, spheroid invasion assay, fluorescence microscopy of the actin cytoskeleton); synthesis of compounds **7–12**, and **S1**; supplementary tables (S1–S3), supplementary figures (S1–S5) and supplementary scheme (S1); characterization of side-products **S3** and **S4**; HPLC purity measurements, including HPLC traces of the final compounds **8**, **11** and **12**; and copies of NMR spectra of all synthesized compounds (PDF)

## ABBREVIATIONS

BLM: human melanoma cell line
CytB: cytochalasin B
CytD: cytochalasin D
DtBAD: di-*tert*-butyl azodicarboxylate
HTMP: 2,2,6,6-tetramethylpiperidine
MRC-5: human fibroblasts
U-2 OS: human cells from osteosarcoma
