# Supplementary material for: Design and Synthesis of Actin-Targeting 10-Phenoxy Cytochalasan Analogues: Balancing Cytotoxicity and Migrastatic Activity
Source: ACS Med Chem Lett. 2026 Jan 9;17(2):409–15. doi: 10.1021/acsmedchemlett.5c00629 (PMC12907919; doi:10.1021/acsmedchemlett.5c00629)
Supplement: Supplementary file 2 [file ml5c00629_si_002.pdf]

Supplementary information

Design and Synthesis of Actin-Targeting 10-Phenoxy Cytochalasan Analogues: Balancing Cytotoxicity and Migrastatic Activity

*Žaneta Javorská,[1] Tereza Volfová,[2] Johan Faivre,[1] Wim Dehaen,[1,3] Silvie Rimpelová,[4] Magdaléna Labíková,[1] Daniel Rösel,[2] Jan Brábek,[2] Pavla Perlíková[1,]**

[1] Department of Organic Chemistry, Faculty of Chemical Technology, University of Chemistry and Technology Prague, Technická 5, 166 28 Prague, Czechia

[2] Department of Cell Biology, BIOCEV, Faculty of Science, Charles University, Průmyslová 595, 252 50 Vestec, Prague West, Czechia

[3] Department of Informatics and Chemistry, Faculty of Chemical Technology, University of Chemistry and Technology Prague, Technická 5, 166 28 Prague, Czechia

[4] Department of Biochemistry and Microbiology, Faculty of Food and Biochemical Technology, University of Chemistry and Technology Prague, Technická 5, 166 28 Prague, Czechia

Table of Contents:

Molecular docking S3
Cytotoxicity assay S5
Actin polymerization assay S6
Spheroid invasion assay S8
Fluorescence microscopy of the actin cytoskeleton S10
Synthesis S10
Supplementary scheme S1 and synthesis of bromide S1 .... S24
Supplementary figure S5 and characterization of side-products S3 and S4 .... S26
HPLC purity of final cytochalasan analogues S28
HPLC traces of final cytochalasan analogues S29
Copies of NMR spectra S31
References S51

Molecular docking

Virtual library construction

The virtual library of 10-substituted cytochalasans was constructed using a custom scripting in Python with the RDKit, a cheminformatics library. Commercially available building blocks were sourced from MCULE, using `[c,CX4]-[SX2H,OX2H]` as a SMARTS query. These building blocks were filtered by molecular weight, retaining only building blocks with a molecular weight under 200 Da. The collected building blocks were conjugated to the cytochalasan core using ReactionSMARTS. Post hoc, a filter was applied to remove compounds containing common protecting groups such as Boc and TBS.

Molecular docking

Ranking of compounds was done using Molecular Docking in the Molecular Operating Environment.[i] The MMFF94x forcefield was used for all energy minimization steps. The crystal structure of cytochalasin D-bound monomeric actin was extracted from the PDB (3EKU),[1] and prepared using the QuickPrep tool. The virtual library was prepared for docking by applying the Wash tool and retaining the dominant predicted protomer at pH 7.4; the structures were minimized, an initial conformation was generated and partial charges were assigned using the in-built tools. Initial placement during docking was done under pharmacophoric constraints, locking the amide in place so it can make the well-known interactions with GLY168 and ALA170 and correctly place the isoindolone core. Initial scoring was done using the London dG scoring function and rescoring was done using the GBVI/WSA scoring function. The rigid docking protocol was selected with 30 initial poses and 5 retained after rescoring. For final ranking, docking scores were corrected for molecular weight dependency using a linear correction. The final 0.1% were then inspected and assessed manually.

[i] *Molecular Operating Environment (MOE)*, 2024.0601 Chemical Computing Group ULC, 910-1010 Sherbrooke St. W., Montreal, QC H3A 2R7, **2025**.

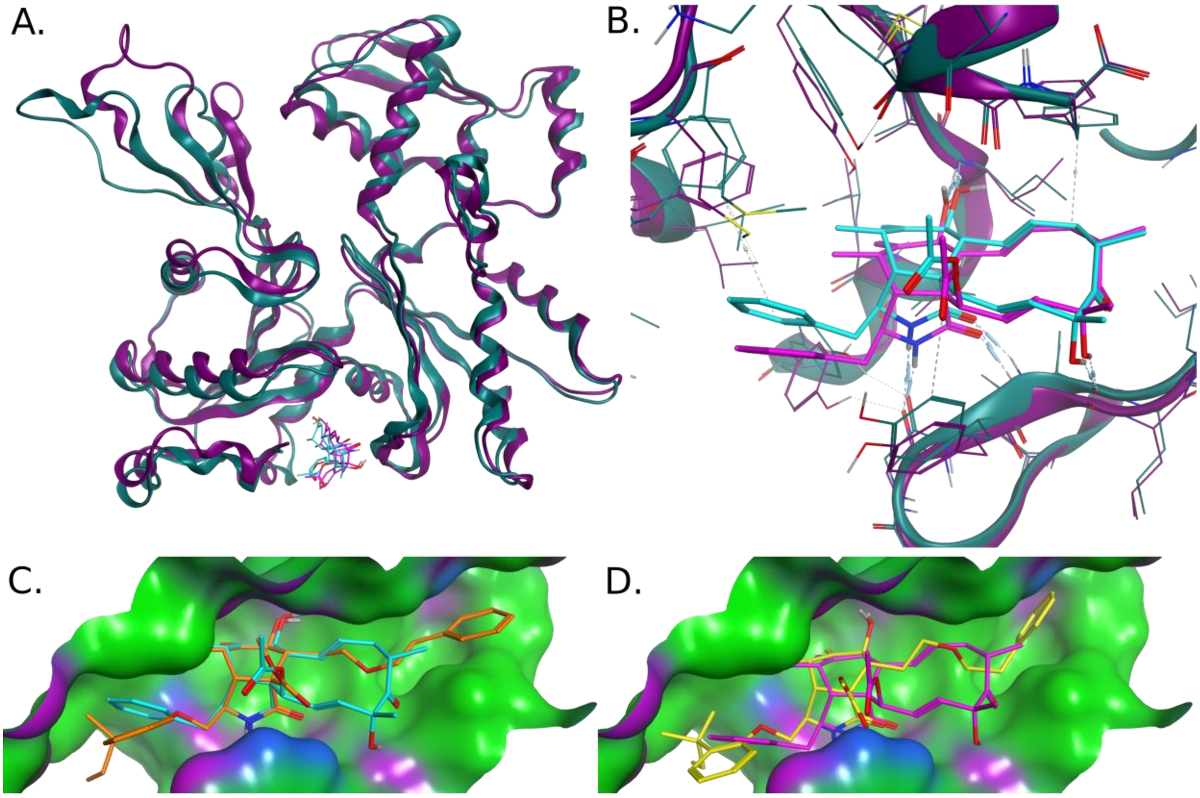

Figure S1. Overlaid structure of 9L2N (purple) and 3EKU (cyan) actin monomer (RMSD(Cα) = 1.749 for full protein) (A); zoom in on CytD (**1**) binding location in 9L2N (purple) and 3EKU (cyan) (pocket overlay RMSD(Cα) = 0.935 when aligning pocket residues), showing pocket residues (B); CytD (**1**, cyan) and **12c** (orange) docked to 3EKU, the pocket surface is colored according to hydrophobic (green), mild polar (blue) and H-bonding (purple) regions (C); CytD (**1**, purple) and **12c** (yellow) docked to 9L2N (D).

Cytotoxicity assay

Cell culture

Human BLM (melanoma), MRC-5 (lung fibroblast (PD-25); Merck, USA), and HaCaT (human keratinocytes; Cell Line Service, Germany) cell lines were used to evaluate cytotoxicity of the studied compounds *in vitro*. The BLM cell line was kindly provided by L. van Kempen and J.H.J.M. van Krieken, Department of Pathology, Radboud University, Nijmegen Medical Centre, the Netherlands. All cell lines were maintained in exponential phase of growth and were routinely passaged using trypsin–EDTA solution. BLM and HaCaT cells were cultured in high-glucose Dulbecco's modified Eagle's medium (DMEM; Merck, USA) supplemented with 10% (*v/v*) fetal bovine serum (FBS; Merck, USA). MRC-5 cells were maintained in minimum essential medium (Merck, USA) containing 10% (*v/v*) FBS and 1% (*v/v*) solution of non-essential amino acids (Merck, USA). Cell cultures were kept at 37 °C in a humidified atmosphere with 5% CO_2.

Cytotoxicity assay

Compound cytotoxicity was assessed using the WST-1 assay (Merck, USA) similarly as reported in ref.[2] Briefly, $5 \cdot 10^3$ BLM, MRC-5 or HaCaT cells were seeded into 96-well plates (Avantor, Czech Republic) in 100 μL of cell culture media per well and incubated for 24 h. The cells were then treated with serial dilutions of the studied compounds (in additional 100 μL of culture media per well) for 72 h. After that, WST-1 solution (4% (*v/v*) in phenol red-free DMEM) was added, and absorbance was measured after 1 h (for BLM cells) and 2 h (for MRC-5 and HaCaT cells), respectively at 450 nm (650 nm as a reference wavelength). Cells treated only with cell culture media served as controls. Experiments were performed three times independently each in three replicates. Half-maximal inhibitory concentrations (IC_{50}) were determined from dose–response curves using AAT Bioquest software.

Actin polymerization assay

The actin polymerization assay was carried out using the Actin Polymerization Biochem Kit™ (Cytoskeleton, Inc., Denver, CO) following the manufacturer's instructions. In brief, 25 µl of G-buffer containing pyrene-labeled actin (0.4 mg/ml) was mixed with 2.48 µl of either DMSO (control, final conc. 0.1% DMSO) or the tested inhibitor (final conc. of the inhibitor 10 µM, 0.1% DMSO) in a 386-well plate. Polymerization was initiated by adding 2.48 µl of 10× Actin Polymerization Buffer, and fluorescence was monitored over time with a spectrophotometer (Infinite 200 PRO, Tecan Life Sciences, Tecan Group Ltd., Zürich, CHE) until the signal reached a plateau. Fluorescence was detected at 420 nm with excitation at 360 nm. For each experiment, the time point at which the G-stock (control) reached 90% of its maximal fluorescence intensity was determined. Data from all fluorescence curves at this time point were obtained, normalized to the control, and analyzed in GraphPad Prism using one-way ANOVA. Each experiment was repeated independently three times, with a minimum of three technical replicates per run. Representative graphs for one measurement are presented in Fig. S2 and S3.

Table S1: Actin polymerization assay at 10 µM concentration.

Compound	Mean difference ± SEM
	Relative actin polymerization (%)
**DMSO**	89.94 ± 7.630
**8a**	81.21 ± 6.652
**8b**	85.44 ± 6.325
**8c**	81.16 ± 6.686
**11**	86.03 ± 6.881
**12a**	67.21 ± 6.384
**12b**	71.06 ± 6.383
**12c**	65.25 ± 6.384
**12d**	65.36 ± 6.383
**12e**	61.46 ± 6.384
**12f**	65.57 ± 6.385
**12g**	63.63 ± 6.384
CytD (**1**)	25.53 ± 6.652

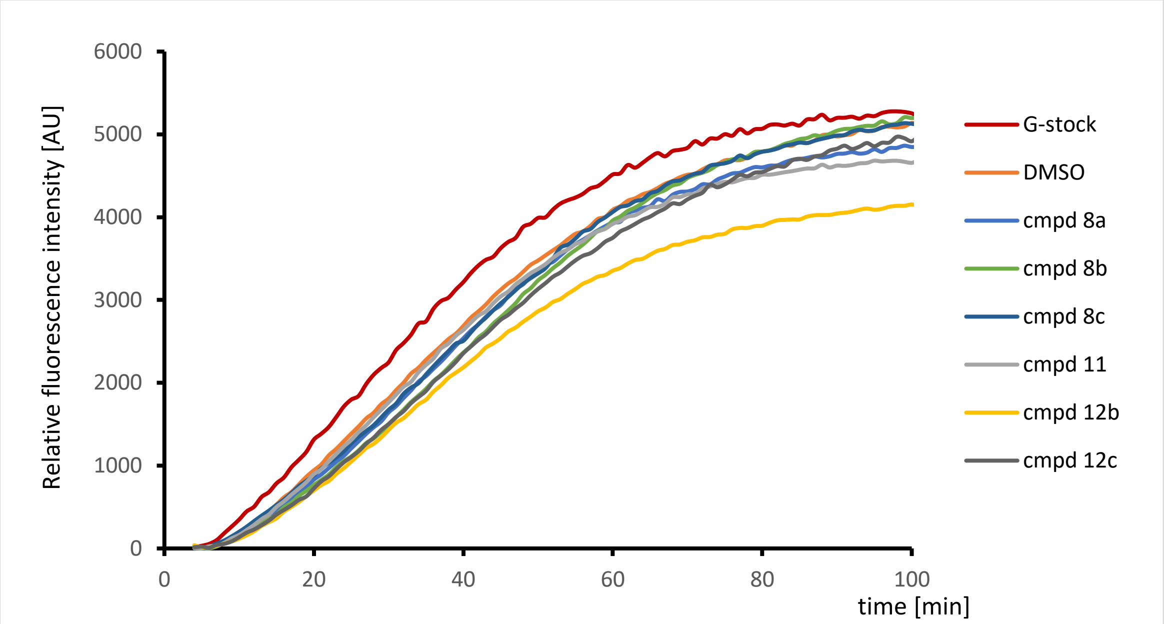

Figure S2: Actin polymerization assay at 10 μM concentration of tested compounds. Representative graph of one measurement with compounds **8a-c**, **11**, **12b,c**.

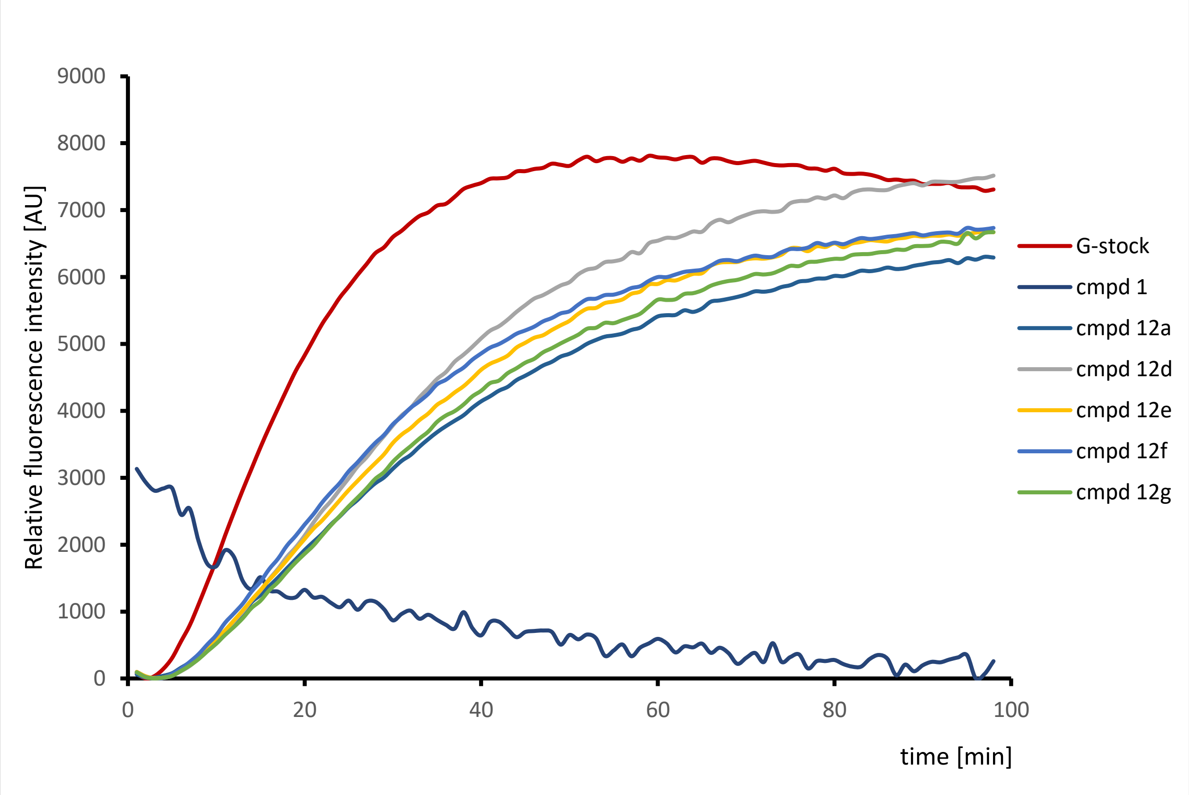

Figure S3: Actin polymerization assay at 10 μM concentration of tested compounds. Representative graph of one measurement **12a,d-g**, and CytD (**1**).

Spheroid invasion assay

The spheroid invasion assay was performed according to the Sartorius protocol (IncuCyte® S3 3D Spheroid Invasion Assay). Human BLM cells were seeded ($4 \cdot 10^3$ cells per well) into a ULA 96-well plate (BIOFLOAT™, SARSTED AG & Co. KG, Germany) and cultured for two days to allow spheroid formation. A collagen type I solution [1.5 mg/ml rat tail collagen (in-lab prepared) in 1× DMEM low-glucose (Merck, USA), 1% FBS (Merck, USA) and 7.5% $NaHCO_3$ (Merck, USA)] was then added into each well. After polymerization at 37 °C in a humidified atmosphere with 5% CO_2, the collagen was overlaid with culture medium containing either inhibitor (final conc. 10 μM) or DMSO (control). The BLM spheroids were imaged with a Leica DMi8 microscope (5×/0.15 dry objective, LAS X Life Science Microscope software) immediately after medium overlay (0 h) and again after 48 h. Cell spread area (outlined by yellow line in Fig. S4) at 48 h was quantified using Fiji software (ImageJ, NIH, Bethesda, MD, USA). Cellular invasiveness was defined as ratio of the cell spread area including the spheroid core to the spheroid core area at 48h. Data were normalized to the control (DMSO) and reported as relative invasion. In three independent experiments, a minimum of eight spheroids were analyzed. Statistical analysis was performed in GraphPad Prism using one-way ANOVA. Representative images of BLM cell spheroid invasion assay are given in Fig S4

Table S2: Spheroid invasion assay with BLM cell line at 10 μM concentration, 48 h.

Compound	Mean ± SE
	Relative invasion
**8a**	1.044±0.056
**8b**	1.005±0.057
**8c**	1.019±0.057
**11**	1.090±0.058
**12a**	0.708±0.055
**12b**	0.949±0.058
**12c**	0.992±0.057
**12d**	0.641±0.058
**12e**	0.623±0.059
**12f**	0.647±0.058
**12g**	0.668±0.059
**1**	0.103±0.060

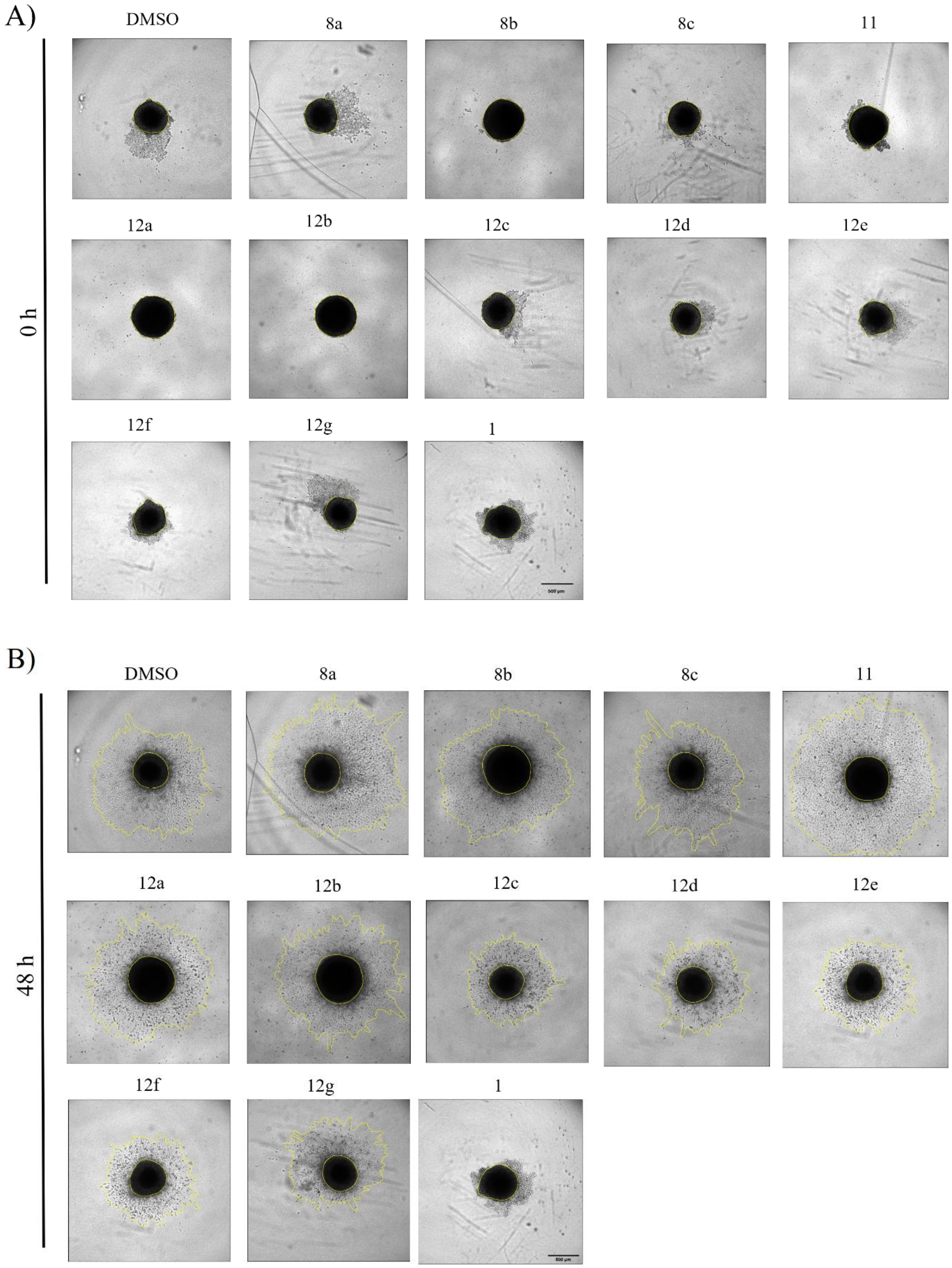

Figure S4. Representative microscopy images of human melanoma cell (BLM) spheroid invasion assay with inhibitors. The cells were treated with CytD (**1**) and the tested compounds **8a-c**, **11**, **12a-g** at 10 µM concentration. Images were taken at 0 h (A) and 48 h (B) after treatment using Leica DMi8 microscope (5×/0.15 dry objective). Cell spheroid treated with DMSO (first image) was used as a control. Scale bar 500 µM.

Fluorescence microscopy of the actin cytoskeleton

U-2 OS cells (human osteosarcoma, ATCC, cat. n. HTB-96) stably expressing LifeAct-tdTomato were seeded at a density of $2{\cdot}10^3$ cells per well in μ-Slide 8-well ibiTreat chambers (ibidi, Germany) and cultured in high-glucose DMEM (Merck, USA) supplemented with 10% (*v/v*) fetal bovine serum (Merck, USA) for 24 h at 37 °C in a humidified atmosphere with 5% CO_2. Initially, cells were imaged under control conditions (untreated) using a Leica DMi8 microscope (40×/0.6 dry objective, LAS X LifeScience Microscope Software). Following the completion of the control recording, compound **12e** was added at final concentrations of 10μM and 50μM, and the same x-y position was recorded every 40 s over a total recording period of 60 min. The red fluorescence of tdTomato was imaged using a 550 nm laser and a QUAD Band Filter cube (575-615nm), laser power was set to 6%. The obtained image sequences were processed and analyzed using Fiji software (Rasband, W.S., ImageJ, NIH, Bethesda, MD, USA).

Synthesis

General remarks

Solvents and chemicals were purchased from commercial suppliers and purified using standard techniques. 2-(Hept-1-yl)phenol,[3] intermediates **5** and **6** (ref.[2]) were synthesized using previously published protocols. Thin-layer chromatography (TLC) was conducted using silica gel plates Merck 60 F254.

All chemical procedures were performed using appropriate personal protective equipment in a fume hood. Handling of pyrophoric reagents followed standard safety protocols.

^{1}H, ^{13}C NMR spectra were measured on Agilent 400-MR DDR2 or JEOL-ECZL400G (400.0 MHz for ^{1}H, 100.6 MHz for ^{13}C). The complete assignment of all NMR signals was done by a combination of H,H-COSY, H,C-HSQC, and H,C-HMBC experiments. For the determination of the stereochemistry of compounds **9a** and **9b**, H,H-ROESY and H,H-NOESY experiments were conducted. The spectra were recorded in $CDCl_3$. It served as an internal standard (δ_{CDCl3} = 7.26 ppm) for ^{1}H NMR and (δ_{CDCl3} = 77.0 ppm) for ^{13}C NMR. HPLC analyses were carried out using an Agilent 1100 Series HPLC system (Agilent Technologies, Waldbronn, Germany). System control and data acquisition were performed using ChemStation software (Agilent Technologies, Waldbronn, Germany). Chromatograms were processed using

OriginPro 2019b (OriginLab Corporation, Northampton, MA, USA). Cytochalasan atom numbering was used for the assignment of the NMR signals.[4] Flash chromatography (FC) was performed using the Büchi Pure C-815 Flash system on CHROMABOND Flash empty cartridges filled with silica gel Silicycle – Siliaflash® P 60 (particle size of 40–63 µm, pore diameter of 60 Å). Purification of some compounds was done by HPLC Büchi Pure C-850 FlashPrep system on a column packed with 5 µm silica-based stationary phase (ProntoSIL 60-5-Si 150 × 20 mm, BISCHOFF Chromatography). Low- and high-resolution mass spectroscopic data were obtained on LTQ Orbitrap XL (Thermo Fisher Scientific) using ESI at the Mass Spectrometry Core Facility, IOCB Prague. Optical rotations were measured at 20 °C, and $[\alpha]_D$ values are given in 10^{-1} deg cm^2 g^{-1} at the Analytical Laboratories, IOCB Prague. All compounds are >95% pure by HPLC analysis.

Methyl (1*R*,3a*R*,4*S*,7*S*,7a*R*)-1-(bromomethyl)-6,7-dimethyl-3-oxo-4-(2-phenethoxyethyl)-1,2,3,4,7,7a-hexahydro-3a*H*-isoindole-3a-carboxylate (7)

Ph_3P (678 mg, 2.58 mmol) and CBr_4 (854 mg, 2.58 mmol) were added to a solution of alcohol **6** (450 mg, 1.12 mmol) in dry DCM (18 mL) at 23 °C under argon atmosphere. Then, the resulting mixture was stirred until full conversion (2 h, TLC, EtOAc) and concentrated under reduced pressure. The crude product was purified by flash chromatography on silica (eluent: EtOAc in hexane 0% to 100%, the product elutes at 65%) to give **7** (421 mg, 81%) as a colorless dense oil. **^{1}H NMR** (400 MHz, $CDCl_3$) δ 7.29–7.16 (m, 5H, H–*o,m,p*-Ph), 6.02 (s, 1H, NH), 5.53 (m, $\sum J$ = 11.3 Hz, 1H, H-7), 3.77 (s, 3H, OCH_3), 3.67 (dt, J_{gem} = 9.3, $J_{15a,16a}$ = $J_{15a,16b}$ = 7.1 Hz, 1H, H-15a), 3.61–3.50 (m, 3H, H-14,15b), 3.41 (m, $\sum J$ = 7.0 Hz, 1H, H-10a), 3.35–3.25 (m, 2H, H-3,10b), 2.87 (t, $J_{16,15a}$ = $J_{16,15b}$ = 7.1 Hz, 2H, H-16), 2.70 (m, $\sum J$ = 13.3 Hz, 1H, H-8), 2.52–2.42 (m, 2H, H-4,5), 2.13 (m, $\sum J$ = 31.7 Hz, 1H, H-13a), 1.91 (m, $\sum J$ = 38.8 Hz, 1H, H-13b), 1.76 (s, 3H, H-12), 1.18 (d, $J_{11,5}$ = 7.0 Hz, 3H, H-11) ppm. **^{13}C NMR** (101 MHz, $CDCl_3$) δ 173.30 (C-1), 172.58 (*C*$OOCH_3$), 139.22 (C–*i*-Ph), 138.61 (C-6), 128.92 (C–*o*-Ph), 128.16 (C–*m*-Ph), 126.96 (C-7), 125.95 (C–*p*-Ph), 71.31 (C-15), 69.66 (C-14), 60.42 (C-9), 55.52 (C-3), 53.46 (C-4), 52.78 (COO*C*H_3), 37.66 (C-10), 36.55 (C-8), 36.29 (C-16), 33.85 (C-5), 29.83 (C-13), 20.10 (C-12), 14.02 (C-11) ppm. **HRMS** (ESI) *m*/*z* calcd for $C_{23}H_{30}O_4N^{79}BrNa$ $[M + Na]^+$ 486.1250; found 486.1245.

General procedure for Mitsunobu reaction

ArOH (1.1–3 eq.) and PPh_3 (1.5–3 eq.) were added to microwave vial and then evacuated and filled with argon (3×). A solution of compound **6** or **11** (1.00 eq.) in anhydrous THF (1–2 mL)

and DtBAD (1.5–3 eq.) were added and the resulting mixture was promptly heated to 90 °C or 120 °C. The reaction mixture was stirred at 90 °C or 120 °C under an argon atmosphere until full conversion (0.5–4 h, TLC) in an oil bath or a microwave reactor. After cooling to room temperature, the mixture was concentrated under reduced pressure. The crude products were purified by flash chromatography and preparative HPLC on silica (eluent: EtOAc in hexane 0% to 70%).

Methyl (1R,3aR,4S,7S,7aR)-6,7-dimethyl-3-oxo-4-(2-phenethoxyethyl)-1-(phenoxymethyl)-1,2,3,4,7,7a-hexahydro-3aH-isoindole-3a-carboxylate (8a)

The reaction was carried out following the general procedure for Mitsunobu reaction: phenol (35 mg, 0.37 mmol), PPh_3 (78 mg, 0.30 mmol), compound **6** (50 mg, 0.12 mmol) and DtBAD (88 mg, 0.38 mmol) in anhydrous THF (2 mL). The reaction mixture was stirred at 120 °C under an argon atmosphere for 1 h (TLC, EtOAc) in a microwave reactor. Flash chromatography (eluent: EtOAc in hexane 0% to 100%, the product elutes at 41% EtOAc), followed by preparative HPLC (eluent: EtOAc in hexane 0% to 100%, the product elutes at 40%) furnished compound **8a** (30 mg, 52%) as a yellowish dense oil. **^{1}H NMR** (400 MHz, $CDCl_3$) δ 7.33 – 7.16 (m, 7H, 2xH-*m*-OPh, 2xH-*o,m*-Ph, H-*p*-Ph), 6.99 (dddd, $J_{\text{H-}p\text{-Bz,H-}m\text{-Bz}}$ = 7.4, $J_{\text{H-}p\text{-Bz,H-}o\text{-Bz}}$ = 1.0 Hz, 1H, H-*p*-OPh), 6.86 (m, ΣJ = 13.3 Hz, 2H, 2xH-*o*-OPh), 6.15 (s, 1H, N-H), 5.56 (s, 1H, H-7), 3.92 (dd, J_{gem} = 8.9, $J_{10a,3}$ = 3.7 Hz, 1H, H-10a), 3.81 – 3.74 (m, 4H, H-10b, OCH_3), 3.69 (dt, J_{gem} = 9.3, $J_{15b,16}$ = 7.1 Hz, 1H, H-15b), 3.62 – 3.52 (m, 3H, H-14a,b,15a), 3.45 (dt, $J_{3,10b}$ = 8.2, $J_{3,10a}$ = $J_{3,4}$ = 3.7 Hz, 1H, H-3), 2.88 (dd, $J_{16,15a}$ = $J_{16,15b}$ = 7.2 Hz, 2H, 2xH-16), 2.75 (dd, $J_{8,13b}$ = 11.3, $J_{8,13a}$ = 2.6 Hz, 1H, H-8), 2.52 – 2.48 (m, 2H, H-4,5), 2.16 (dddd, J_{gem} = 14.0, $J_{13a,14a}$ = $J_{13a,14b}$ = 7.5, $J_{13a,8}$ = 2.6 Hz, 1H, H-13a), 1.94 (dddd, J_{gem} = 14.1, $J_{13b,8}$ = 11.0, $J_{13b,14b}$ = $J_{13b,14a}$ = 5.4 Hz, 1H, H-13b), 1.78 (s, 3H, 3xH-12), 1.20 (d, $J_{11,5}$ = 7.1 Hz, 3H, 3xH-11) ppm. **^{13}C NMR** (101 MHz, $CDCl_3$) δ 173.66 (C-1), 172.89 (*C*OOCH$_3$), 157.99 (C-*i*-OPh), 139.25 (C-*i*-Ph), 138.86 (C-6), 129.58 (C-*m*-OPh), 128.95 (C-*o*-Ph), 128.18 (C-*m*-Ph), 126.90 (C-7), 125.96 (C-*p*-Ph), 121.43 (C-*p*-OPh), 114.27 (C-*o*-OPh), 71.98 (C-10), 71.32 (C-15), 69.76 (C-14), 59.68 (C-9), 53.31 (C-3), 52.77 (COO*C*H$_3$), 50.68 (C-4), 36.45 (C-8), 36.32 (C-16), 33.91 (C-5), 29.90 (C-13), 20.20 (C-12), 14.05 (C-11) ppm. **HRMS** (ESI) m/z calcd for $C_{29}H_{35}O_5NNa$ $[M + Na]^+$ 500.2407; found 500.2406. $[\alpha]_D$ = +0.8° (*c* 0.152; $CHCl_3$).

Methyl (1*R*,3a*R*,4*S*,7*S*,7a*R*)-1-((2-heptylphenoxy)methyl)-6,7-dimethyl-3-oxo-4-(2-phenethoxyethyl)-1,2,3,4,7,7a-hexahydro-3aH-isoindole-3a-carboxylate (8b)

The reaction was carried out following the general procedure for Mitsunobu reaction: 2-(hept-1-yl)phenol (71 mg, 0.37 mmol), PPh_3 (94 mg, 0.36 mmol), compound **6** (50 mg, 0.12 mmol) and DtBAD (85 mg, 0.37 mmol) in anhydrous THF (2 mL). The reaction mixture was stirred at 120 °C under an argon atmosphere for 1 h (TLC, EtOAc) in a microwave reactor. Flash chromatography (eluent: EtOAc in hexane 0% to 100%, the product elutes at 41% EtOAc) furnished compound **8b** (13 mg, 19%) as a yellowish dense oil. **^{1}H NMR** (400 MHz, $CDCl_3$) δ 7.30 – 7.18 (m, 5H, H-*o,m,p*-Ph), 7.18 – 7.11 (m, 2H, H-3', H-5'), 6.92 (td, $J_{4',5'} = J_{4',3'} = 7.4$, $J_{4',6'} = 1.1$ Hz, 1H, H-4'), 6.78 (dd, $J_{6',5'} = 8.5$, $J_{6',4'} = 1.2$ Hz, 1H, H-6'), 6.00 (s, 1H, NH), 5.57 (m, $\sum J = 8.0$ Hz, 1H, H-7), 3.92 (dd, $J_{gem} = 9.0$, $J_{10a,3} = 3.8$ Hz, 1H, H-10a), 3.82 – 3.77 (m, 3H, H-10b, OCH_3), 3.69 (dt, $J_{gem} = 9.4$, $J_{15a,16} = 7.1$ Hz, 1H, H-15a), 3.63 – 3.52 (m, 3H, 2× H-14, H-15b), 3.46 (dt, $J_{3,10b} = 8.6$, $J_{3,4} = J_{3,10a} = 3.8$ Hz, 1H, H-3), 2.88 (t, $J = 7.2$ Hz, 2H, H-16), 2.76 (dd, $J_{8,13b} = 11.0$, $J_{8,13a} = 4.0$ Hz, 1H, H-8), 2.58 (t, $J_{17,18} = 3.6$ Hz, 2H, H-17), 2.52 – 2.48 (m, 2H, H-4, H-5), 2.17 (ddt, $J_{gem} = 14.0$, $J_{13a,14} = 6.8$, $J_{13a,8} = 4.0$ Hz, 1H, H-13a), 1.93 (ddt, $J_{gem} = 14.0$, $J_{13b,8} = 11.0$, $J_{13b,14} = 5.4$ Hz, 1H, H-13b), 1.79 (s, 3H, H-12), 1.58 (m, $\sum J = 24.0$ Hz, 2H, H-18), 1.38 – 1.24 (m, 8H, 2× H-22, 2× H-21, 2× H-20, 2× H-19), 1.21 (d, $J = 7.0$ Hz, 3H, H-11), 0.88 (m, $\sum J = 13.6$ Hz, 3H, H-23) ppm. **^{13}C NMR** (101 MHz, $CDCl_3$) δ 173.76 (C-1), 172.97 (*C*OO*C*H_3), 155.87 (C-1'-OPh), 139.41 (C-*i*-Ph), 138.95 (C-6), 131.34 (C-2'-OPh), 130.16 (C-3'-OPh), 129.07 (C-*o*-Ph), 128.31 (C-*m*-Ph), 127.13 (C-7), 126.89 (C-5'-OPh), 126.08 (C-*p*-Ph), 121.38 (C-4'-OPh), 111.21 (C-6'-OPh), 72.56 (C-10), 71.46 (C-15), 69.92 (C-14), 59.73 (C-9), 53.63 (C-3), 52.89 (COO*C*H_3), 50.88 (C-4), 36.59 (C-8), 36.46 (C-16), 34.01 (C-5), 31.92 (C-19/C-20/C-21/C-22), 30.10 (C-13/C-18), 30.07 (C-13/C-18), 30.00 (C-17), 29.62 (C-19/C-20/C-21/C-22), 29.32 (C-19/C-20/C-21/C-22), 22.76 (C-19/C-20/C-21/C-22), 20.35 (C-12), 14.22 (C-11/C-23), 14.19 (C-11/C-23) ppm. **HRMS** (ESI) m/z calcd for $C_{36}H_{49}O_5NNa$ $[M + Na]^+$ 598.3503; found 598.3498. $[\alpha]_D = -9.7°$ (*c* 0.156; $CHCl_3$).

Methyl (1*R*,3a*R*,4*S*,7*S*,7a*R*)-6,7-dimethyl-3-oxo-1-((2-(*tert*-pentyl)phenoxy)methyl)-4-(2-phenethoxyethyl)-1,2,3,4,7,7a-hexahydro-3aH-isoindole-3a-carboxylate (8c)

Compound **7** (40 mg, 86.1 µmol), K_2CO_3 (60 mg, 0.431 mmol) and NaI (5 mg, 28.4 µmol) were added to the microwave vial and then evacuated and filled with argon (3×). Subsequently, dry acetonitrile (2 mL) and 2-(1,1-dimethylpropyl)phenol (43 mg, 0.258 mmol) were added at 23 °C under argon atmosphere and the resulting mixture was stirred at 70 °C until full conversion (28 h, TLC, hexane/EtOAc: 1/1). The reaction mixture was concentrated under reduced pressure and EtOAc (15 mL) was added. The resulting mixture was washed with 1M

NaOH (2 × 20 mL) and aqueous phase was extracted with EtOAc (3 × 20 mL). The combined organic layers were washed brine (1 × 15 mL), dried ($MgSO_4$) and the solvent was evaporated *in vacuo*. The crude product was purified by flash chromatography on silica gel (eluent: EtOAc in hexane 0% to 100%, the product elutes at 33%) to give **8c** (33 mg, 70%) as a colorless dense oil. **^{1}H NMR** (400 MHz, $CDCl_3$) δ 7.31 – 7.15 (m, 7H, H-3',5', 2xH-*o,m*-Ph, H-*p*-Ph), 6.94 (ddd, $J_{4',3'}$ = $J_{4',5'}$ = 7.5, $J_{4',6'}$ = 1.3 Hz, 1H, H-4'), 6.81 (dd, $J_{6',5'}$ = 8.1, $J_{6',4'}$ = 1.3 Hz, 1H, H-6'), 5.98 (s, 1H, N-H), 5.58 (s, 1H, H-7), 3.91 – 3.88 (m, 2H, H-10a,b), 3.80 (s, 3H, OCH_3), 3.70 (dt, J_{gem} = 9.3, $J_{15b,16}$ = 7.1 Hz, 1H, H-15b), 3.63 – 3.53 (m, 3H, H-14a,b,15a), 3.47 (ddd, $J_{3,10b}$ = 8.0, $J_{3,4}$ = 4.6, $J_{3,10a}$ = 4.2 Hz, 1H, H-3), 2.89 (dd, $J_{16,15a}$ = $J_{16,15b}$ = 7.2 Hz, 2H, 2xH-16), 2.77 (dd, $J_{8,13b}$ = 11.3, $J_{8,13a}$ = 2.5 Hz, 1H, H-8), 2.51 (m, ΣJ = 20.2 Hz, 1H, H-5), 2.47 (dd, $J_{4,3}$ = $J_{4,5}$ = 4.6 Hz, 1H, H-4), 2.19 (dddd, J_{gem} = 14.2, $J_{13a,14a}$ = $J_{13a,14b}$ = 7.5, $J_{13a,8}$ = 2.6 Hz, 1H, H-13a), 1.92 (dddd, J_{gem} = 14.1, $J_{13b,8}$ = 11.0, $J_{13b,14b}$ = $J_{13b,14a}$ = 5.4 Hz, 1H, H-13b), 1.84 – 1.74 (m, 5H, 3xH-12, 2xH-19), 1.336 (s, 3H, 3xH-18a), 1.329 (s, 3H, 3xH-18b), 1.19 (d, $J_{11,5}$ = 7.2 Hz, 3H, 3xH-11), 0.64 (t, $J_{20,19}$ = 7.5 Hz, 3H, 3xH-20) ppm. **^{13}C NMR** (101 MHz, $CDCl_3$) δ 173.57 (C-1), 172.83 (*C*OOCH$_3$), 156.84 (C-1'), 139.24 (C-*i*-Ph), 138.78 (C-6), 136.16 (C-2'), 128.95 (C-*o*-Ph), 128.36 (C-3'), 128.18 (C-*m*-Ph), 127.05 (C-5'/7), 127.00 (C-5'/7), 125.96 (C-*p*-Ph), 121.20 (C-4'), 112.18 (C-6'), 73.01 (C-10), 71.33 (C-15), 69.76 (C-14), 59.50 (C-9), 53.70 (C-3), 52.84 (COO*C*H$_3$), 50.53 (C-4), 38.43 (C-17), 36.37 (C-8/16), 36.32 (C-8/16), 33.83 (C-5/12/19), 33.79 (C-5/12/19), 29.96 (C-13), 28.25 (C-18a), 28.09 (C-18b), 20.33 (C-12/19), 14.11 (C-11), 9.53 (C-20) ppm. **HRMS** (ESI) m/z calcd for $C_{34}H_{45}O_5NNa$ $[M + Na]^+$ 570.3190; found 570.3189. $[\alpha]_D$ = −7.7° (*c* 0.175; $CHCl_3$).

Methyl (1a*S*,2*R*,2a*S*,5*R*,5a*R*,6*S*,6a*R*)-4-benzoyl-5-(((*tert*-butyldimethylsilyl)oxy)methyl)-6,6a-dimethyl-3-oxo-2-(2-phenethoxyethyl)octahydro-2aH-oxireno[2,3-f]isoindole-2a-carboxylate (9a) and methyl (1a*R*,2*R*,2a*S*,5*R*,5a*R*,6*S*,6a*S*)-4-benzoyl-5-(((tert-butyldimethylsilyl)oxy)methyl)-6,6a-dimethyl-3-oxo-2-(2-phenethoxyethyl)octahydro-2aH-oxireno[2,3-f]isoindole-2a-carboxylate (9b)

To a solution of alkene **5** (1.00 g, 1.61 mmol) in DCM (15 mL), *m*CPBA (475 mg, 1.93 mmol, 70% (w/w)) was added at 0 °C. The resulting mixture was stirred for 1.5 h at 0 °C and at room temperature until full conversion (20 h, TLC, hexane/EtOAc: 4/1). Then, the reaction mixture was diluted with DCM (50 mL) and washed with a solution of $NaHCO_3$ (2 × 15 mL). The aqueous phase was extracted with DCM (1 × 50 mL). The combined organic layers were washed with brine (15 mL), dried ($MgSO_4$) and concentrated under reduced pressure. The diastereomeric mixture was purified by flash chromatography on silica (eluent: EtOAc in

hexane 0% to 20%, the products elute at 15%), affording the two diastereomers **9a** and **9b** (**9a**: 756 mg, 74%; **9b**: 185 mg, 18%) as yellow dense oils. **9a:** **^{1}H NMR** (400 MHz, $CDCl_3$) δ 7.77 (m, *ΣJ* = 15.2 Hz, 2H, 2xH-*o*-Bz), 7.56 (dddd, $J_{H\text{-}p\text{-}Bz,H\text{-}m\text{-}Bz}$ = 7.4, $J_{H\text{-}p\text{-}Bz,H\text{-}o\text{-}Bz}$ = 1.3 Hz, 1H, H-*p*-Bz), 7.45 (m, *ΣJ* = 15.2 Hz, 2H, 2xH-*m*-Bz), 7.30 – 7.15 (m, 5H, H-*p*-Ph, 2xH-*o,m*-Ph), 4.57 (ddd, $J_{3,10a/b}$ = 7.1, $J_{3,10a/b}$ = 4.4, $J_{3,4}$ = 2.7 Hz, 1H, H-3), 3.84 (s, 3H, OCH_3), 3.74 – 3.55 (m, 5H, 2xH-10,15, H-14a), 3.51 (ddd, J_{gem} = 9.9 Hz, $J_{14b,13b}$ = 8.4 Hz, $J_{14b,13a}$ = 5.4 Hz, 1H, H-14b), 2.86 (t, $J_{16,15}$ = 7.2 Hz, 2H, 2xH-16), 2.79 (dd, $J_{4,5}$ = 6.4 Hz, $J_{4,3}$ = 2.7 Hz, 1H, H-4), 2.74 (d, $J_{7,8}$ = 5.7 Hz, 1H, H-7), 2.38 (ddt, J_{gem} = 13.9 Hz, $J_{13a,8}$ = 11.6 Hz, $J_{13a,14a}$ = $J_{13a,14b}$ = 5.2 Hz, 1H, H-13a), 2.20 (ddd, $J_{8,13a}$ = 11.5 Hz, $J_{8,7}$ = 5.7 Hz, $J_{8,13b}$ = 2.6 Hz, 1H, H-8), 2.00 (dq, $J_{5,11}$ = 7.5 Hz, $J_{5,4}$ = 6.6 Hz, 1H, H-5), 1.72 (dddd, J_{gem} = 13.8 Hz, $J_{13b,14b}$ = 8.1 Hz, $J_{13b,14a}$ = 6.0 Hz, $J_{13b,8}$ = 2.7 Hz, 1H, H-13b), 1.27 – 1.24 (m, 6H, 3xH-11,12), 0.86 (s, 9H, $C(CH_3)_3$), 0.03 (s, 3H, $SiCH_3$), 0.02 (s, 3H, $SiCH_3$) ppm. **^{13}C NMR** (101 MHz, $CDCl_3$) δ 172.17 (*C*$OOCH_3$), 171.97 (C-1), 170.54 (C=O–Bz), 139.12 (C-*i*-Ph), 133.70 (C-*i*-Bz), 132.76 (C-*p*-Bz), 129.33 (C-*o*-Bz), 128.95 (C-*o*-Ph), 128.17 (C-*m*-Ph), 128.09 (C-*m*-Bz), 125.96 (C-*p*-Ph), 71.79 (C-10), 68.63 (C-14), 63.60 (C-15), 61.40 (C-7), 60.72 (C-9), 56.40 (C-6), 55.95 (C-3), 53.02 (COO*C*H_3), 44.20 (C-4), 39.72 (C-8), 36.26 (C-16), 36.08 (C-5), 29.19 (C-13), 25.79 (SiC*C*H_3), 18.93 (C-11/12), 18.30 (Si*C*CH_3), 12.80 (C-11/12), -5.57 (Si*C*H_3), -5.60 (Si*C*H_3) ppm. **HRMS** (ESI) m/z calcd for $C_{36}H_{49}O_7NNaSi$ $[M + Na]^+$ 658.3171; found 658.3169.

9b: **^{1}H NMR** (400 MHz, $CDCl_3$) δ 7.75 (m, *ΣJ* = 11.8 Hz, 2H, H-*o*-Bz), 7.49 (dddd, $J_{H\text{-}p\text{-}Bz,H\text{-}m\text{-}Bz}$ = 7.4, $J_{H\text{-}p\text{-}Bz,H\text{-}o\text{-}Bz}$ = 1.3 Hz, 1H, H-*p*-Bz), 7.39 (m, *ΣJ* = 16.2 Hz, 2H, H-*m*-Bz), 7.27 (m, *ΣJ* = 14.3 Hz, 2H, H-*m*-Ph), 7.23 – 7.16 (m, 3H, H-*p*-Ph, 2xH-*o*-Ph), 4.40 (ddd, $J_{3,4}$ = $J_{3,10a}$ = 4.4, $J_{3,10b}$ = 2.7 Hz, 1H, H-3), 3.89 (dd, J_{gem} = 10.5, $J_{10a,3}$ = 4.4 Hz, 1H, H-10a), 3.79 (s, 3H, OCH_3), 3.66 – 3.53 (m, 3H, H-10b, 2xH-15), 3.53 – 3.39 (m, 2H, H-14a,b), 2.95 (s, 1H, H-7), 2.84 (t, $J_{16,15}$ = 6.9 Hz, 2H, 2xH-16), 2.78 (dd, $J_{8,13a}$ = 10.5, $J_{8,13b}$ = 2.5 Hz, 1H, H-8), 2.63 (dd, $J_{4,5}$ = 6.0, $J_{4,3}$ = 4.5 Hz, 1H, H-4), 2.50 (dq, $J_{5,11}$ = 7.4, $J_{5,4}$ = 6.2 Hz, 1H, H-5), 2.27 (dddd, J_{gem} = 15.0, $J_{13a,8}$ = 10.7, $J_{13a,14a/b}$ = 6.2, $J_{13a,14a/b}$ = 4.5 Hz, 1H, H-13a), 1.89 (dddd, J_{gem} = 15.1, $J_{13b,14a/b}$ = 7.7, $J_{13b,14a/b}$ = 5.2, $J_{13b,8}$ = 2.5 Hz, 1H, H-13b), 1.24 (s, 3H, 3xH-12), 1.21 (d, $J_{11,5}$ = 7.3 Hz, 3H, 3xH-11), 0.85 (s, 9H, $C(CH_3)_3$), -0.01 (s, 3H, $SiCH_3$), -0.02 (s, 3H, $SiCH_3$) ppm. **^{13}C NMR** (101 MHz, $CDCl_3$) δ 172.84 (C-1), 172.70 (*C*$OOCH_3$), 171.19 (C=O–Bz), 139.20 (C-*i*-Ph), 135.07 (C-*i*-Bz), 131.97 (C-*p*-Bz), 129.11 (C-*o*-Bz), 128.88 (C-*o*-Ph), 128.25 (C-*m*-Ph), 127.83 (C-*m*-Bz), 126.06 (C-*p*-Ph), 71.29 (C-15), 69.42 (C-14), 62.69 (C-10), 60.24 (C-7), 58.57 (C-9), 58.29 (C-6), 57.23 (C-3), 52.93 (COO*C*H_3), 41.53 (C-4), 37.56 (C-8), 36.28 (C-16), 33.43 (C-5), 29.02 (C-13), 25.89 (SiC*C*H_3), 19.45 (C-12), 18.41 (Si*C*CH_3), 13.58 (C-

11), -5.47 (Si*C*H_3), -5.60 (Si*C*H_3) ppm. **HRMS** (ESI) m/z calcd for $C_{36}H_{49}O_7NNaSi$ $[M + Na]^+$ 658.3171; found 658.3169.

Methyl (1*R*,3a*S*,4R,5*S*,7*S*,7a*R*)-1-(((*tert*-butyldimethylsilyl)oxy)methyl)-5-hydroxy-7-methyl-6-methylene-3-oxo-4-(2-phenethoxyethyl)octahydro-3aH-isoindole-3a-carboxylate (10)

Precaution: Please note that BuLi and Et_2AlCl are highly reactive reagents that require careful handling due to their pyrophoric nature and potential health hazards.

To a solution of HTMP (1 mL, 5.95 mmol) in toluene (5 mL), BuLi (2.4 mL, 2.5 M in hexanes, 6.06 mmol) was added at 0 °C. The resulting mixture was stirred for 45 minutes at 0 °C under an argon atmosphere. Following this, Et_2AlCl (5.9 mL, 1.0 M in hexanes, 5.95 mmol) was added slowly dropwise, and the mixture was stirred for an additional 80 min at 0 °C. Next, a solution of epoxide **9a** (756 mg, 1.19 mmol) in toluene (5+5 mL) was added dropwise. The mixture was stirred at 0 °C for 50 minutes and at 23 °C under argon atmosphere until full conversion (4.5 h, TLC, hexane/EtOAc: 7:3). At 0 °C, dry methanol (5 mL) and a small piece of sodium (approx. 3 × 3 × 3 mm) were added. The resulting mixture was allowed to warm up to 23 °C and stirred under an argon atmosphere until full conversion (1 h, TLC, hexane/EtOAc: 1:1). Then a saturated solution of $NaHCO_3$ (2 mL) was added, and the reaction mixture was concentrated under reduced pressure. A 1 M solution of $NaHSO_4$ (20 mL) and EtOAc (20 mL) were added, and the resulting phases were separated. The aqueous phase was extracted with EtOAc (2 × 20 mL) and the combined organic layers were washed with a 1M solution of $NaHSO_4$ (15 mL), brine (15 mL), dried ($MgSO_4$) and concentrated under reduced pressure. The crude product was purified by flash chromatography on silica (eluent: EtOAc in hexane 0% to 50%, the product elutes at 47%), affording the alcohol **10** (340 mg, 54%) as a yellow dense oil. **^{1}H NMR** (400 MHz, $CDCl_3$) δ 7.28 (m, ΣJ = 19.0 Hz, 2H, 2xH-*m*-Ph), 7.22 – 7.17 (m, 3H, H-*p*-Ph, 2xH-*o*-Ph), 5.98 (s, 1H, NH), 5.30 (s, 1H, H-12a), 5.05 (s, 1H, H-12b), 4.72 (d, $J_{7,OH}$ = 1.2 Hz, 1H, OH), 3.97 (dd, $J_{7,8}$ = 9.4, $J_{7,OH}$ = 1.1 Hz, 1H, H-7), 3.76 (s, 3H, OCH_3), 3.72 – 3.63 (m, 3H, H-14a, 2xH-15), 3.55 – 3.41 (m, 3H, 2xH-10, H-14b), 3.23 (m, ΣJ = 17.6 Hz, 1H, H-3), 2.94 – 2.87 (m, 3H, H-5, 2xH-16), 2.54 (ddd, $J_{8,7}$ = 9.5, $J_{8,13a/13b}$ = 7.6, $J_{8,13a/13b}$ = 2.2 Hz, 1H, H-8), 2.31 (dd, $J_{4,3/5}$ = 5.2, $J_{4,3/5}$ = 4.2 Hz, 1H, H-4), 2.14 (m, ΣJ = 25.2 Hz, 1H, H-13a), 1.98 (m, ΣJ = 41.2 Hz, 1H, H-13b), 1.06 (d, $J_{11,5}$ = 6.8 Hz, 3H, 3xH-11), 0.89 (s, 9H, $C(CH_3)_3$), 0.054 (s, 3H, $SiCH_3$), 0.052 (s, 3H, $SiCH_3$) ppm. **^{13}C NMR** (101 MHz, $CDCl_3$) δ 173.14 (C-1/*C*$OOCH_3$), 172.97 (C-1/*C*$OOCH_3$), 148.31 (C-6), 138.27 (C-*i*-Ph), 128.86 (C-*o*-Ph), 128.37 (C-*m*-Ph), 126.25 (C-*p*-Ph), 113.37 (C-12), 72.82 (C-7), 72.11 (C-15), 71.29 (C-14),

67.13 (C-10), 57.24 (C-9), 54.03 (C-3), 52.83 (COO*C*H_3), 46.93 (C-4), 42.84 (C-8), 35.96 (C-16), 30.90 (C-5/C-13), 30.87 (C-5/C-13), 25.82 (SiC*C*H_3), 18.24 (Si*C*CH_3), 13.94 (C-11), -5.47 (Si*C*H_3) ppm. **HRMS** (ESI) m/z calcd for $C_{29}H_{45}O_6NNaSi$ $[M + Na]^+$ 554.2908; found 554.2906.

Methyl (1*R*,3a*S*,4*R*,5*S*,7*S*,7a*R*)-5-hydroxy-1-(hydroxymethyl)-7-methyl-6-methylene-3-oxo-4-(2-phenethoxyethyl)octahydro-3aH-isoindole-3a-carboxylate (11)

To a solution of compound **10** (340 mg, 0.64 mmol) in dry acetonitrile (5 mL), triethylamine trihydrofluoride (630 µL, 3.86 mmol) was added at 23 °C under argon atmosphere and the resulting mixture was stirred until full conversion (20 h, TLC, EtOAc). A saturated solution of $NaHCO_3$ (2 mL) was slowly added (release of gas) and the reaction mixture was concentrated under reduced pressure. A saturated solution of $NaHCO_3$ (10 mL) and EtOAc (10mL) were added, and the resulting phases were separated. The aqueous phase was extracted with EtOAc (3 × 10 mL). The combined organic layers were washed with brine (10 mL), dried ($MgSO_4$) and the solvent was evaporated in vacuo. The crude product was purified by flash chromatography on silica gel (eluent: EtOAc in hexane 50% to 100%, the product elutes at 100%) to give **11** (250 mg, 94%) as a white amorphous solid. **^{1}H NMR** (400 MHz, $CDCl_3$) δ 7.28 (m, ΣJ = 19.1 Hz, 2H, 2xH-*m*-Ph), 7.20 (m, 3H, H-*p*-Ph, 2xH-*o*-Ph), 7.13 (s, 1H, NH), 5.30 (s, 1H, H-12a), 5.06 (s, 1H, H-12b), 4.76 (d, $J_{7,OH}$ = 1.6 Hz, 1H, sec. OH), 3.93 (dd, $J_{7,8}$ = 9.7, $J_{7,OH}$ = 1.2 Hz, 1H, H-7), 3.75 (s, 3H, OCH_3), 3.72 – 3.61 (m, 3H, H-14a, 2xH-15), 3.58 (m, ΣJ = 11.5 Hz, 1H, H-10a), 3.48 – 3.36 (m, 2H, H-10b,14b), 3.29 (m, ΣJ = 13.5 Hz, 1H, H-3), 3.12 (s, 1H, prim. OH), 2.95 – 2.88 (m, 3H, H-5, 2xH-16), 2.51 (ddd, $J_{7,8}$ = 9.7, $J_{8,13a/b}$ = 6.7, $J_{8,13a/b}$ = 3.1 Hz, 1H, H-8), 2.43 (dd, $J_{4,3/5}$ = 5.5, $J_{4,3/5}$ = 3.9 Hz, 1H, H-4), 2.11 – 1.98 (m, 2H, H-13a,b), 1.08 (d, $J_{11,5}$ = 6.8 Hz, 3H, 3xH-11) ppm. **^{13}C NMR** (101 MHz, $CDCl_3$) δ 174.34 (C-1), 173.27 (*C*OOCH_3), 148.29 (C-6), 138.22 (C-*i*-Ph), 128.84 (C-*o*-Ph), 128.39 (C-*m*-Ph), 126.28 (C-*p*-Ph), 113.52 (C-12), 72.79 (C-7), 72.09 (C-15), 71.13 (C-14), 65.88 (C-10), 57.90 (C-9), 54.66 (C-3), 52.93 (COO*C*H_3), 46.46 (C-4), 42.75 (C-8), 35.94 (C-16), 31.02 (C-5), 30.79 (C-13), 13.71 (C-11) ppm. **HRMS** (ESI) m/z calcd for $C_{23}H_{31}O_6NNa$ $[M + Na]^+$ 440.2044; found 440.2043. $[\alpha]_D$ = +44.8° (*c* 0.183; $CHCl_3$).

Methyl (1*R*,3a*S*,4*R*,5*S*,7*S*,7a*R*)-5-hydroxy-7-methyl-6-methylene-3-oxo-4-(2-phenethoxyethyl)-1-(phenoxymethyl)octahydro-3aH-isoindole-3a-carboxylate (12a)

The reaction was carried out following the general procedure for Mitsunobu reaction: phenol (9 mg, 92.2 µmol), PPh_3 (33 mg, 0.13 mmol), compound **11** (35 mg, 83.8 µmol) and DtBAD

(29 mg, 0.13 mmol) in anhydrous THF (1.5 mL). The reaction mixture was stirred at 90 °C under an argon atmosphere for 1 h (TLC, EtOAc) in an oil bath. Flash chromatography (eluent: EtOAc in hexane 0% to 100%, the product elutes at 50% EtOAc), followed by preparative HPLC (eluent: EtOAc in hexane 0% to 100%, the product elutes at 50%) furnished compound **12a** (19 mg, 46%) as a yellowish dense oil. **^{1}H NMR** (400 MHz, $CDCl_3$) δ 7.33 – 7.26 (m, 4H, 2xH-*m*-Ph, 2xH-*m*-OPh), 7.24 – 7.18 (m, 3H, H-*p*-Ph, 2xH-*o*-Ph), 6.99 (t, $J_{p\text{-OPh},m\text{-OPh}}$ = 7.3 Hz, 1H, H-*p*-OPh), 6.85 (dd, $J_{o\text{-OPh},m\text{-OPh}}$ = 8.6, $J_{o\text{-OPh},o\text{-OPh}}$ = 1.2 Hz, 2H, 2xH-*o*-OPh), 6.18 (s, 1H, NH), 5.34 (s, 1H, H-12a), 5.10 (s, 1H, H-12b), 4.00 (d, $J_{7,8}$ = 9.4, 1H, H-7), 3.90 (dd, J_{gem} = 8.9, $J_{10a,3}$ = 3.9 Hz, 1H, H-10a), 3.83 (dd, J_{gem} = $J_{10b,3}$ = 8.8 Hz, 1H, H-10b), 3.77 (s, 3H, OCH_3), 3.74 – 3.64 (m, 3H, H-14a, 2xH-15), 3.58 (m, ΣJ = 17.3, 1H, H-3), 3.50 (ddd, J_{gem} = $J_{14b,13a/b}$ = 9.9, $J_{14b,13a/b}$ = 2.6 Hz, 1H, H-14b), 2.98 (qd, $J_{11,5}$ = 6.8 Hz, $J_{4,5}$ = 5.2 Hz, 1H, H-5), 2.92 (dd, $J_{16,15a}$ = $J_{16,15b}$ = 7.4 Hz, 2H, 2xH-16), 2.56 (m, ΣJ = 20.1 Hz, 1H, H-8), 2.51 (dd, $J_{4,5}$ = 5.2, $J_{4,3}$ = 3.5 Hz, 1H, H-4), 2.18 (m, ΣJ = 29.1 Hz, 1H, H-13a), 2.03 (m, ΣJ = 39.1 Hz, 1H, H-13b), 1.13 (d, $J_{11,5}$ = 6.8 Hz, 3H, 3xH-11) ppm. **^{13}C NMR** (101 MHz, $CDCl_3$) δ 173.10 (C-1), 172.94 (*C*$OOCH_3$), 158.00 (C-*i*-OPh), 147.98 (C-6), 138.24 (C-*i*-Ph), 129.62 (C-*m*-OPh), 128.88 (C-*o*-Ph), 128.40 (C-*m*-Ph), 126.29 (C-*p*-Ph), 121.49 (C-*p*-OPh), 114.28 (C-*o*-OPh), 113.83 (C-12), 72.84 (C-7), 72.15 (C-15), 71.31 (C-14), 71.23 (C-10), 57.10 (C-9), 53.00 (COO*C*H_3), 51.62 (C-3), 47.35 (C-4), 42.95 (C-8), 35.97 (C-16), 31.06 (C-5), 31.02 (C-13), 13.87 (C-11) ppm. **HRMS** (ESI) m/z calcd for $C_{29}H_{35}O_6NNa$ $[M + Na]^+$ 516.2357; found 516.2353. $[\alpha]_D$ = −1.9° (*c* 0.159; $CHCl_3$).

Methyl (1*R*,3a*S*,4*R*,5*S*,7*S*,7a*R*)-1-((2-heptylphenoxy)methyl)-5-hydroxy-7-methyl-6-methylene-3-oxo-4-(2-phenethoxyethyl)octahydro-3aH-isoindole-3a-carboxylate (12b)

The reaction was carried out following the general procedure for Mitsunobu reaction: 2-(hept-1-yl)phenol (69 mg, 0.36 mmol), PPh_3 (95 mg, 0.36 mmol), compound **11** (50 mg, 0.12 mmol) and DtBAD (83 mg, 0.36 mmol) in anhydrous THF (2 mL). The reaction mixture was stirred at 120 °C under an argon atmosphere for 4 h (TLC, EtOAc) in a microwave reactor. Flash chromatography (eluent: EtOAc in hexane 0% to 100%, the product elutes at 40% EtOAc), followed by preparative HPLC (eluent: EtOAc in hexane 0% to 100%, the product elutes at 30%) furnished compound **12b** (9 mg, 13%) as a yellowish dense oil. **^{1}H NMR** (400 MHz, $CDCl_3$) δ 7.29 (m, ΣJ = 18.8 Hz, 2H, 2xH-*m*-Ph), 7.24 – 7.17 (m, 3H, H-*p*-Ph, 2×H-*o*-Ph), 7.17 – 7.11 (m, 2H, H-3‘,5‘), 6.93 (ddd, $J_{4',3'}$ = $J_{4',5'}$ = 7.4, $J_{4',6'}$ = 1.1 Hz, 1H, H-4‘), 6.76 (dd, $J_{6',5'}$ = 8.6, $J_{6',4'}$ = 1.2 Hz, 1H, H-6‘), 5.96 (s, 1H, NH), 5.36 (s, 1H, H-12a), 5.12 (s, 1H, H-12b), 4.79 (d, $J_{OH,7}$ = 1.5 Hz, 1H, OH), 4.01 (dd, $J_{7,8}$ = 8.6, $J_{7,OH}$ = 1.1 Hz, 1H, H-7),

3.89 (dd, J_{gem} = 8.9, $J_{10a,3}$ = 4.7 Hz, 1H, H-10a), 3.84 (dd, J_{gem} = $J_{10b,3}$ = 8.9 Hz, 1H, H-10b), 3.78 (s, 3H, OCH_3), 3.74 – 3.65 (m, 3H, H-14a, 2xH-15), 3.59 (ddd, $J_{3,10b}$ = 8.8 Hz, $J_{3,10a}$ = 4.5 Hz, $J_{3,4}$ = 3.8 Hz, 1H, H-3), 3.51 (ddd, J_{gem} = $J_{14b,13a/b}$ = 9.9, $J_{14b,13a/b}$ = 2.9 Hz, 1H, H-14b), 2.98 (qd, $J_{5,11}$ = 6.7, $J_{5,4}$ = 5.7 Hz, 1H, H-5), 2.92 (dd, $J_{16,15a}$ = $J_{16,15b}$ = 7.4 Hz, 2H, 2xH-16), 2.64 – 2.53 (m, 3H, H-8, 2xH-17), 2.51 (dd, $J_{4,5}$ = 5.4, $J_{4,3}$ = 3.8 Hz, 1H, H-4), 2.19 (m, ΣJ = 24.7 Hz, 1H, H-13a), 2.03 (m, ΣJ = 37.7 Hz, 1H, H-13b), 1.55 (m, ΣJ = 16.1 Hz, 2H, 2xH-18), 1.35 – 1.24 (m, 8H, 2xH-19,20,21,22), 1.15 (d, $J_{11,5}$ = 6.9 Hz, 3H, 3xH-11), 0.89 (t, $J_{23,22}$ = 6.8 Hz, 3H, 3xH-23) ppm. **^{13}C NMR** (101 MHz, $CDCl_3$) δ 172.99 (C-1, *C*OOCH$_3$), 155.78 (C-1‘), 148.11 (C-6), 138.28 (C-*i*-Ph), 131.30 (C-2‘), 130.10 (C-3‘), 128.89 (C-*o*-Ph), 128.42 (C-*m*-Ph), 126.82 (C-5‘), 126.31 (C-*p*-Ph), 121.33 (C-4‘), 113.79 (C-12), 111.09 (C-6‘), 72.84 (C-7), 72.18 (C-15), 71.68 (C-10), 71.33 (C-14), 57.03 (C-9), 53.01 (COO*C*H$_3$), 51.73 (C-3), 47.54 (C-4), 42.92 (C-8), 36.01 (C-16), 31.83 (C-19/20/21/22), 31.08 (C-5/13), 31.03 (C-5/13), 29.98 (C-17/18), 29.94 (C-17/18), 29.52 (C-19/20/21/22), 29.24 (C-19/20/21/22), 22.66 (C-19/20/21/22), 14.11 (C-23), 13.96 (C-11) ppm. **HRMS** (ESI) m/z calcd for $C_{36}H_{49}O_6NNa$ [M + Na]$^+$ 614.3452; found 614.3449. $[\alpha]_D$ = −2.9° (*c* 0.159; $CHCl_3$).

Methyl (1*R*,3a*S*,4*R*,5*S*,7*S*,7a*R*)-5-hydroxy-7-methyl-6-methylene-3-oxo-1-((2-(*tert*-pentyl)phenoxy)methyl)-4-(2-phenethoxyethyl)octahydro-3aH-isoindole-3a-carboxylate (12c)

The reaction was carried out following the general procedure for Mitsunobu reaction: 2-(1,1-dimethylpropyl)phenol (9 mg, 53.9 μmol), PPh_3 (14 mg, 53.9 μmol), compound **11** (15 mg, 35.9 μmol) and DtBAD (12 mg, 53.9 μmol) in anhydrous THF (1 mL). The reaction mixture was stirred at 90 °C under an argon atmosphere for 1 h (TLC, EtOAc) in an oil bath. Flash chromatography (eluent: EtOAc in hexane 0% to 100%, the products elute at 40% EtOAc) furnished main product **12c** (9 mg, 45%) as a yellowish dense oil and side product **S3** (3 mg, 12%, Figure S5) as a yellowish dense oil. **^{1}H NMR** (400 MHz, $CDCl_3$) δ 7.33 – 7.14 (m, 7H, H-3’,5’, 2xH-*o,m*-Ph, H-*p*-Ph), 6.95 (ddd, $J_{4‘,3‘}$ = $J_{4‘,5‘}$ = 7.5, $J_{4‘,6‘}$ = 1.3 Hz, 1H, H-4‘), 6.79 (dd, $J_{6‘,5‘}$ = 8.2, $J_{6‘,4‘}$ = 1.3 Hz, 1H, H-6’), 5.96 (s, 1H, NH), 5.38 (s, 1H, H-12a), 5.12 (s, 1H, H-12b), 4.83 (d, $J_{OH,7}$ = 1.4 Hz, 1H, OH), 4.01 (dd, $J_{7,8}$ = 9.6, $J_{7,OH}$ = 1.2 Hz, 1H, H-7), 3.96 – 3.84 (m, 2H, H-10a,b), 3.80 (s, 3H, OCH_3), 3.74 – 3.66 (m, 3H, H-14a, 2xH-15), 3.61 (m, ΣJ = 17.3 Hz, 1H, H-3), 3.51 (ddd, J_{gem} = 10.4, $J_{14b,13a/b}$ = 9.9, $J_{14b,13a/b}$ = 2.9 Hz, 1H, H-14b), 2.99 (qd, $J_{5,11}$ = 6.7, $J_{5,4}$ = 5.3 Hz, 1H, H-5), 2.92 (dd, $J_{16,15a}$ = $J_{16,15b}$ = 7.4 Hz, 2H, 2xH-16), 2.58 (ddd, $J_{8,7}$ = 9.6, $J_{8,13a/b}$ = 7.6, $J_{8,13a/b}$ = 2.1 Hz, 1H, H-8), 2.47 (dd, $J_{4,5}$ = 5.2, $J_{4,3}$ = 4.2 Hz, 1H, H-4), 2.19 (m, ΣJ = 25.3 Hz, 1H, H-13a), 2.02 (m, ΣJ = 37.6 Hz, 1H, H-13b), 1.79 (m,

ΣJ = 38.4 Hz, 2H, H-19a,b), 1.336 (s, 3H, 3xH-18a), 1.333 (s, 3H, 3xH-18b), 1.13 (d, $J_{11,5}$ = 6.8 Hz, 3H, 3xH-11), 0.64 (t, $J_{20,19}$ = 7.5 Hz, 3H, 3xH-20) ppm. **^{13}C NMR** (101 MHz, $CDCl_3$) δ 172.94 (C-1, *C*OOCH$_3$), 156.85 (C-1’), 147.94 (C-6), 138.24 (C-*i*-Ph), 136.31 (C-2’), 128.89 (C-*o*-Ph), 128.43 (C-3’), 128.41 (C-*m*-Ph), 127.08 (C-5’), 126.31 (C-*p*-Ph), 121.30 (C-4’), 113.91 (C-12), 112.29 (C-6’), 72.69 (C-7), 72.39 (C-10), 72.18 (C-15), 71.30 (C-14), 57.01 (C-9), 53.08 (COO*C*H$_3$), 51.92 (C-3), 47.23 (C-4), 42.91 (C-8), 38.46 (C-17), 35.98 (C-16), 33.79 (C-19), 31.02 (C-5), 30.94 (C-13), 28.26 (C-18a), 28.13 (C-18b), 14.01 (C-11), 9.55 (C-20) ppm. **HRMS** (ESI) m/z calcd for $C_{34}H_{45}O_6NNa$ $[M + Na]^+$ 586.3139; found 586.3134. $[\alpha]_D = -11.1°$ (*c* 0.186; $CHCl_3$).

Methyl (1*R*,3a*S*,4*R*,5*S*,7*S*,7a*R*)-5-hydroxy-7-methyl-6-methylene-3-oxo-4-(2-phenethoxyethyl)-1-((p-tolyloxy)methyl)octahydro-3aH-isoindole-3a-carboxylate (12d)

The reaction was carried out following the general procedure for Mitsunobu reaction: 4-methylphenol (12 mg, 0.11 mmol), PPh_3 (28 mg, 0.11 mmol), compound **11** (30 mg, 71.8 μmol) and DtBAD (24 mg, 0.11 mmol) in anhydrous THF (1 mL). The reaction mixture was stirred at 90 °C under an argon atmosphere for 1 h (TLC, EtOAc) in an oil bath. Flash chromatography (eluent: EtOAc in hexane 0% to 100%, the product elutes at 65% EtOAc), followed by preparative HPLC (eluent: EtOAc in hexane 0% to 100%, the product elutes at 57%) furnished compound **12d** (6 mg, 17%) as a yellowish dense oil. **^{1}H NMR** (399 MHz, $CDCl_3$) δ 7.29 (m, ΣJ = 21.3 Hz, 2H, 2xH-*m*-Ph), 7.24 – 7.16 (m, 3H, H-*p*-Ph, 2×H-*o*-Ph), 7.09 (m, 2H, ΣJ = 8.95 Hz, 2xH-3‘), 6.75 (m, ΣJ = 8.92 Hz, 2H, 2xH-2‘), 6.01 (s, 1H, HN), 5.34 (s, 1H, H-12a), 5.10 (s, 1H, H-12b), 4.00 (d, $J_{7,8}$ = 9.6 Hz, 1H, H-7), 3.87 (dd, J_{gem} = 8.9, $J_{10a,3}$ = 4.1 Hz, 1H, H-10a), 3.77 (s, 4H, H-10b, OCH_3), 3.73 – 3.65 (m, 3H, H-14a, 2xH-15), 3.56 (ddd, $J_{3,10b}$ = 8.7, $J_{3,10a}$ = 4.1, $J_{3,4}$ = 3.9, 1H, H-3), 3.50 (ddd, J_{gem} = 10.5, $J_{14b,13b}$ = 9.2, $J_{14b,13a}$ = 2.8 Hz, 1H, H-14b), 2.97 (qd, $J_{5,11}$ = 6.7, $J_{5,4}$ = 5.4, 1H, H-5), 2.91 (t, $J_{16,15}$ = 7.4 Hz, 2H, 2xH-16), 2.56 (ddd, $J_{8,7}$ = 9.6, $J_{8,13b}$ = 7.4, $J_{8,13a}$ = 2.0 Hz, 1H, H-8), 2.48 (dd, $J_{4,5}$ = 5.1, $J_{4,3}$ = 3.9 Hz, 1H, H-4), 2.29 (s, 3H, Ph-CH$_3$), 2.18 (dddd, J_{gem} = 15.5, $J_{13a,14a}$ = 4.3, $J_{13a,14b}$ = 2.6, $J_{13a,8}$ = 2.0, 1H, H-13a), 2.03 (m, ΣJ = 40.5 Hz, 1H, H-13b), 1.13 (d, $J_{11,5}$ = 6.7 Hz, 3H, 3×H-11) ppm. **^{13}C NMR** (101 MHz, $CDCl_3$) δ 172.98 (C-1, *C*OOCH$_3$), 155.92 (C-1‘), 147.98 (C-6), 138.25 (C-*i*-Ph), 130.83 (C-4‘), 130.05 (C-3‘), 128.90 (C-*o*-Ph), 128.41 (C-*m*-Ph), 126.31 (C-*p*-Ph), 114.13 (C-2‘), 113.83 (C-12), 72.83 (C-7), 72.18 (C-15), 71.49 (C-10), 71.33 (C-14), 57.05 (C-9), 53.01 (COO*C*H$_3$), 51.63 (C-3), 47.35 (C-4), 42.99 (C-8), 35.98 (C-16), 31.05 (C-5,13), 20.47 (Ph-*C*H$_3$), 13.88 (C-11) ppm. **HRMS** (ESI) m/z calcd for $C_{30}H_{37}O_6NNa$ $[M + Na]^+$ 530.2513; found 530.2515. $[\alpha]_D = -7.3°$ (*c* 0.178; $CHCl_3$).

Methyl (1*R*,3a*S*,4*R*,5*S*,7*S*,7a*R*)-5-hydroxy-1-((4-methoxyphenoxy)methyl)-7-methyl-6-methylene-3-oxo-4-(2-phenethoxyethyl)octahydro-3a*H*-isoindole-3a-carboxylate (12e)

The reaction was carried out following the general procedure for Mitsunobu reaction: 4-methoxyphenol (10 mg, 79.0 µmol), PPh_3 (28 mg, 0.11 mmol), compound **11** (30 mg, 71.8 µmol) and DtBAD (24 mg, 0.11 mmol) in anhydrous THF (1 mL). The reaction mixture was stirred at 90 °C under an argon atmosphere for 0.5 h (TLC, EtOAc) in an oil bath. Flash chromatography (eluent: EtOAc in hexane 0% to 100%, the product elutes at 65% EtOAc), followed by preparative HPLC (eluent: EtOAc in hexane 0% to 100%, the product elutes at 60%) furnished compound **12e** (6 mg, 16%) as a yellowish dense oil. **^{1}H NMR** (399 MHz, $CDCl_3$) δ 7.29 (m, ΣJ = 20.7 Hz, 2H, 2xH-*m*-Ph), 7.24 – 7.17 (m, 3H, H-*p*-Ph, 2×H-*o*-Ph), 6.87 – 6.74 (m, 4H, 2xH-2‘,3‘), 6.02 (s, 1H, HN), 5.34 (s, 1H, H-12a), 5.10 (s, 1H, H-12b), 4.00 (d, $J_{7,8}$ = 9.6 Hz, 1H, H-7), 3.85 (dd, J_{gem} = 8.8, $J_{10a,3}$ = 4.0 Hz, 1H, H-10a), 3.78 – 3.74 (m, 7H, H-10b, $PhOCH_3$, OCH_3), 3.72 – 3.64 (m, 3H, H-14a, 2xH-15), 3.60 – 3.45 (m, 2H, H-3,14b), 2.97 (qd, $J_{5,11}$ = 7.1, $J_{5,4}$ = 5.4, 1H, H-5), 2.91 (t, $J_{16,15}$ = 7.4 Hz, 2H, 2xH-16), 2.56 (ddd, $J_{8,7}$ = 9.5, $J_{8,13a/b}$ = 7.5, $J_{8,13a/b}$ = 2.0 Hz, 1H, H-8), 2.47 (dd, $J_{4,5}$ = 5.2, $J_{4,3}$ = 3.8 Hz, 1H, H-4), 2.17 (m, ΣJ = 25.2 Hz, 1H, H-13a), 2.03 (m, ΣJ = 39.5 Hz, 1H, H-13b), 1.13 (d, $J_{11,5}$ = 6.9 Hz, 3H, 3xH-11) ppm. **^{13}C NMR** (101 MHz, $CDCl_3$) δ 172.99 (C-1, *C*$OOCH_3$), 154.31 (C-4‘), 152.15 (C-1‘), 147.98 (C-6), 138.24 (C-*i*-Ph), 128.89 (C-*o*-Ph), 128.41 (C-*m*-Ph), 126.31 (C-*p*-Ph), 115.30 (C-3‘), 114.72 (C-2‘), 113.83 (C-12), 72.82 (C-7), 72.18 (C-10,15), 71.33 (C-14), 57.06 (C-9), 55.70 (COO*C*H_3/PhO*C*H_3), 53.01 (COO*C*H_3/PhO*C*H_3), 51.67 (C-3), 47.32 (C-4), 43.00 (C-8), 35.98 (C-16), 31.04 (C-5,13), 13.88 (C-11) ppm. **HRMS** (ESI) m/z calcd for $C_{30}H_{37}O_7NNa$ $[M + Na]^+$ 546.2462; found 546.2464. $[\alpha]_D = -3.9°$ (*c* 0.144; $CHCl_3$).

Methyl (1*R*,3a*S*,4*R*,5*S*,7*S*,7a*R*)-1-((4-chlorophenoxy)methyl)-5-hydroxy-7-methyl-6-methylene-3-oxo-4-(2-phenethoxyethyl)octahydro-3a*H*-isoindole-3a-carboxylate (12f)

The reaction was carried out following the general procedure for Mitsunobu reaction: 4-chlorophenol (10 mg, 79.0 µmol), PPh_3 (28 mg, 0.11 mmol), compound **11** (30 mg, 71.8 µmol) and DtBAD (24 mg, 0.11 mmol) in anhydrous THF (1 mL). The reaction mixture was stirred at 90 °C under an argon atmosphere for 0.5 h (TLC, EtOAc) in an oil bath. Flash chromatography (eluent: EtOAc in hexane 0% to 100%, the product elutes at 65% EtOAc), followed by preparative HPLC (eluent: EtOAc in hexane 0% to 100%, the product elutes at 56%) furnished compound **12f** (7 mg, 18%) as a yellowish dense oil. **^{1}H NMR** (400 MHz, $CDCl_3$) δ 7.32 – 7.17 (m, 7H, 2xH-*m*-Ph, H-*p*-Ph, 2xH-*o*-Ph, 2xH-3’), 6.78 (m, ΣJ = 15.4

Hz, 2H, 2xH-2'), 6.05 (s, 1H, NH), 5.34 (s, 1H, H-12a), 5.10 (s, 1H, H-12b), 3.99 (d, $J_{7,8}$ = 9.5 Hz, 1H, H-7), 3.86 (dd, J_{gem} = 8.9, $J_{10a,3}$ = 4.1 Hz, 1H, H-10a), 3.80 – 3.76 (s, 4H, H-10b, OCH_3), 3.73 – 3.64 (m, 3H, H-14a, 2xH-15), 3.56 (ddd, $J_{3,10b}$ = 8.2, $J_{3,10a}$ = $J_{3,4}$ = 4.1 Hz, 1H, H-3), 3.50 (ddd, J_{gem} = 10.4, $J_{14b,13a/b}$ = 9.2, $J_{14b,13a/b}$ = 2.8 Hz, 1H, H-14b), 2.98 (qd, $J_{5,11}$ = 6.8, $J_{5,4}$ = 5.2 Hz, 1H, H-5), 2.91 (t, $J_{16,15}$ = 7.4 Hz, 2H, 2xH-16), 2.55 (ddd, $J_{7,8}$ = 9.7, $J_{8,13a/b}$ = 7.6, $J_{8,13a/b}$ = 2.1 Hz, 1H, H-8), 2.48 (dd, $J_{4,5}$ = 5.3, $J_{4,3}$ = 4.1 Hz, 1H, H-4), 2.22 – 2.12 (m, ΣJ = 27.2 Hz, 1H, H-13a), 2.08 – 1.97 (m, ΣJ = 38.1 Hz, 1H, H-13b), 1.13 (d, $J_{11,5}$ = 6.8 Hz, 3H, 3xH-11) ppm. **^{13}C NMR** (101 MHz, $CDCl_3$) δ 173.02 (C-1/*C*OOCH3), 172.89 (C-1/*C*OOCH3), 156.60 (C-1'), 147.89 (C-6), 138.22 (C-*i*-Ph), 129.51 (C-3'), 128.89 (C-*o*-Ph), 128.41 (C-*m*-Ph), 126.46 (C-4'), 126.32 (C-*p*-Ph), 115.56 (C-2'), 113.90 (C-12), 72.81 (C-7), 72.18 (C-15), 71.64 (C-10), 71.30 (C-14), 57.01 (C-9), 53.03 (COO*C*H3), 51.45 (C-3), 47.33 (C-4), 42.98 (C-8), 35.97 (C-16), 31.06 (C-5,13), 13.87 (C-11) ppm. **HRMS** (ESI) m/z calcd for $C_{29}H_{34}O_6NClNa$ $[M + Na]^+$ 550.1967; found 550.1969. $[\alpha]_D$ = −7.8° (*c* 0.158; $CHCl_3$).

Methyl (1*R*,3a*S*,4*R*,5*S*,7*S*,7a*R*)-1-((3,4-dichlorophenoxy)methyl)-5-hydroxy-7-methyl-6-methylene-3-oxo-4-(2-phenethoxyethyl)octahydro-3aH-isoindole-3a-carboxylate (12g)

The reaction was carried out following the general procedure for Mitsunobu reaction: 3,4-dichlorophenol (18 mg, 0.11 mmol), PPh_3 (28 mg, 0.11 mmol), compound **11** (30 mg, 71.8 µmol) and DtBAD (25 mg, 0.11 mmol) in anhydrous THF (1 mL). The reaction mixture was stirred at 90 °C under an argon atmosphere for 2 h (TLC, EtOAc) in an oil bath. Flash chromatography (eluent: EtOAc in hexane 0% to 100%, the products elute at 65% EtOAc), followed by preparative HPLC (eluent: EtOAc in hexane 0% to 100%, the products elute at 56%) furnished compound **12g** (6 mg, 15%) as a yellowish dense oil and side product **S4** (4 mg, 8%, Figure S5) as a yellowish dense oil. **^{1}H NMR** (400 MHz, $CDCl_3$) δ 7.34 (d, $J_{5',6'}$ = 8.9 Hz, 1H, H-5'), 7.29 (m, ΣJ = 25.3 Hz, 2H, 2xH-*m*-Ph), 7.24 – 7.18 (m, 3H, H-*p*-Ph, 2×H-*o*-Ph), 6.95 (d, $J_{2',6'}$ = 2.9 Hz, 1H, H-2'), 6.72 (dd, $J_{6',5'}$ = 8.9, $J_{6',2'}$ = 2.9 Hz, 1H, H-6'), 6.09 (s, 1H, NH), 5.35 (s, 1H, H-12a), 5.10 (s, 1H, H-12b), 3.98 (d, $J_{7,8}$ = 9.6 Hz, 1H, H-7), 3.85 (dd, J_{gem} = 8.6, $J_{10a,3}$ = 4.3 Hz, 1H, H-10a), 3.80 – 3.77 (m, 4H, H-10b, OCH_3), 3.75 – 3.63 (m, 3H, H-14a, 2xH-15), 3.56 (ddd, $J_{3,10b}$ = 8.2, $J_{3,10a}$ = 4.2, $J_{3,4}$ = 4.0 Hz, 1H, H-3), 3.49 (ddd, J_{gem} = 10.5, $J_{14b,13a/b}$ = 9.2, $J_{14b,13a/b}$ = 2.6 Hz, 1H, H-14b), 2.98 (qd, $J_{5,11}$ = 6.8, $J_{5,4}$ = 5.4, 1H, H-5), 2.91 (t, $J_{16,15}$ = 7.4 Hz, 2H, 2xH-16), 2.55 (ddd, $J_{7,8}$ = 9.5, $J_{8,13a/b}$ = 6.7, $J_{8,13a/b}$ = 2.0 Hz, 1H, H-8), 2.48 (dd, $J_{4,5}$ = 5.2, $J_{4,3}$ = 4.0 Hz, 1H, H-4), 2.17 (m, ΣJ = 25.7 Hz, 1H, H-13a), 2.09 – 1.96 (m, ΣJ = 37.5 Hz, 1H, H-13b), 1.13 (d, $J_{11,5}$ = 6.9 Hz, 3H, 3xH-11) ppm. **^{13}C NMR** (101 MHz, $CDCl_3$) δ 173.05 (C-1), 172.83 (*C*OOCH3), 156.99 (C-1'), 147.84 (C-6), 138.21

(C-*i*-Ph), 133.06 (C-3‘), 130.89 (C-5‘), 128.89 (C-*o*-Ph), 128.42 (C-*m*-Ph), 126.33 (C-*p*-Ph), 124.91 (C-4‘), 116.09 (C-2‘), 114.45 (C-6‘), 113.97 (C-12), 72.79 (C-7), 72.19 (C-15), 71.83 (C-10), 71.29 (C-14), 57.00 (C-9), 53.09 (COO*C*H_3), 51.33 (C-3), 47.31 (C-4), 42.97 (C-8), 35.97 (C-16), 31.06 (C-3,15), 13.86 (C-11) ppm. **HRMS** (ESI) m/z calcd for $C_{29}H_{33}O_6NCl_2Na$ $[M + Na]^+$ 584.1577; found 584.1581. $[\alpha]_D = -7.8°$ (*c* 0.146; $CHCl_3$).

Supplementary scheme S1 and synthesis of bromide S1

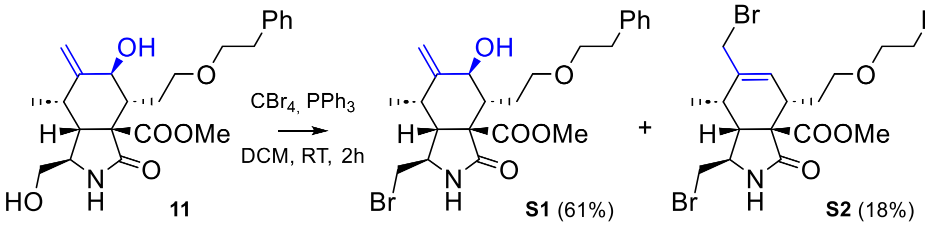

Scheme S1. The optimized reaction conditions of the Appel reaction

Methyl (1*R*,3a*S*,4*R*,5*S*,7*S*,7a*R*)-1-(bromomethyl)-5-hydroxy-7-methyl-6-methylene-3-oxo-4-(2-phenethoxyethyl)octahydro-3aH-isoindole-3a-carboxylate (S1) and methyl (1*R*,3a*R*,4*S*,7*S*,7a*R*)-1,6-bis(bromomethyl)-7-methyl-3-oxo-4-(2-phenethoxyethyl)-1,2,3,4,7,7a-hexahydro-3aH-isoindole-3a-carboxylate (S2)

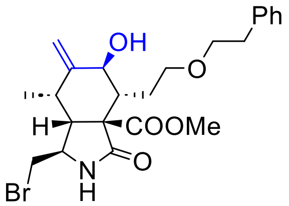

Ph_3P (189 mg, 0.72 mmol) and CBr_4 (238 mg, 0.72 mmol) were added to a solution of compound **6** (95 mg, 0.23 mmol) in dry DCM (5 mL), at 23 °C under argon atmosphere. The resulting mixture was stirred until full conversion (2 h, TLC, EtOAc). Then, the mixture was concentrated under reduced pressure. The crude product was purified by flash chromatography on silica (eluent: EtOAc in hexane 0% to 100%, the products elute at 66%), followed by preparative HPLC (eluent: EtOAc in hexane 0% to 100%, the products elute at 40%), yielding compound **S1** (67 mg, 61%) as a colorless dense oil and compound **S2** (22 mg, 18%) as a yellowish dense oil. **S1: ^{1}H NMR** (400 MHz, $CDCl_3$) δ 7.28 (m, ΣJ = 20.6 Hz, 2H, 2xH-*m*-Ph), 7.22 – 7.15 (m, 3H, H-*p*-Ph, 2xH-*o*-Ph), 6.04 (s, 1H, NH), 5.31 (s, 1H, H-12a), 5.07 (s, 1H, H-12b), 4.78 (d, $J_{OH,7}$ = 1.1 Hz, 1H, OH), 3.94 (dd, $J_{7,8}$ = 9.3 Hz, $J_{7,OH}$ = 1.1 Hz, 1H, H-7), 3.77 (s, 3H, OCH_3), 3.72 – 3.61 (m, 3H, H-14a, 2xH-15), 3.52 – 3.39 (m, 2H, H-3,14a), 3.33 (m, ΣJ = 40.4 Hz, 2H, 2xH10), 2.96 – 2.88 (m, 3H, H-5, 2xH-16), 2.50 (ddd, $J_{8,7}$ = 9.6, $J_{8,13a/13b}$ = 7.4, $J_{8,13a/13b}$ = 2.1 Hz, 1H, H-8), 2.44 (dd, $J_{4,3/5}$ = 5.3, $J_{4,3/5}$ = 3.6 Hz, 1H, H-4), 2.13 (m, ΣJ = 26.0 Hz, 1H, H-13a), 1.99 (m, ΣJ = 39.0 Hz, 1H, H-13b), 1.11 (d, $J_{11,5}$ = 6.8 Hz, 3H, 3×H-11) ppm. **^{13}C NMR** (101 MHz, $CDCl_3$) δ 172.82 (C-1/*C*OOCH₃), 172.66 (C-1/*C*OOCH₃), 147.84 (C-6), 138.34 (C-*i*-Ph), 128.98 (C-*o*-Ph), 128.52 (C-*m*-Ph), 126.42 (C-*p*-Ph), 114.07 (C-12), 72.95 (C-7), 72.27 (C-15), 71.35 (C-14), 57.75 (C-9), 53.83 (C-3), 53.17 (COO*C*H₃), 50.27 (C-4), 43.08 (C-8), 36.93 (C-10), 36.08 (C16), 31.16 (C5/13), 31.13 (C5/13), 13.98 (C-11) ppm. **HRMS** (ESI) m/z calcd for $C_{23}H_{30}O_5N^{79}BrNa$ $[M + Na]^+$ 502.1200; found 502.1196.

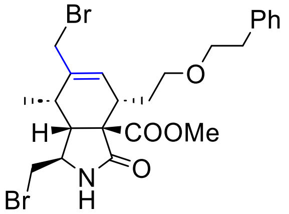

S2: **^{1}H NMR** (400 MHz, $CDCl_3$) δ 7.26 (m, ΣJ = 16.3 Hz, 2H, 2xH-*m*-Ph), 7.23 – 7.15 (m, 3H, H-*p*-Ph, 2xH-*o*-Ph), 6.09 (s, 1H, NH), 5.98 (m, ΣJ = 6.5 Hz, 1H, H-7), 3.96 (m, ΣJ = 23.0 Hz, 2H, 2xH-12), 3.78 (s, 3H, OCH_3), 3.71 – 3.61 (m, 2H, H-3,15a), 3.57 (m, ΣJ = 17.0 Hz, 1H, H-15b), 3.51 (m, ΣJ = 24.5 Hz, 2H, 2xH-14), 3.41 (dd, J_{gem} = 10.2, $J_{10a,3}$ = 3.9 Hz, 1H, H-10a), 3.30 (dd, J_{gem} = 10.3, $J_{10b,3}$ = 9.0 Hz, 1H, H-10b), 2.85 (dd, $J_{16,15a}$ = $J_{16,15b}$ = 7.0 Hz, 2H, 2xH-16), 2.75 (m, ΣJ = 10.3 Hz, 1H, H-8), 2.55 (m, ΣJ = 17.2 Hz, 1H, H-5), 2.49 (dd, $J_{4,3/5}$ = 5.2, $J_{4,3/5}$ = 3.8 Hz, 1H, H-4), 2.14 – 1.94 (m, 2H, H-13a,b), 1.37 (d, $J_{11,5}$ = 7.2 Hz, 3H, 3×H-11) ppm. **^{13}C NMR** (101 MHz, $CDCl_3$) δ 172.81 (C-1), 172.40 (*C*$OOCH_3$), 139.36 (C-*i*-Ph), 138.16 (C-6), 135.65 (C-7), 129.05 (C-*o*-Ph), 128.33 (C-*m*-Ph), 126.12 (C-*p*-Ph), 71.48 (C-15), 69.75 (C-14), 60.16 (C-9), 55.38 (C-3), 53.10 (COO*C*H_3), 52.76 (C-4), 37.55 (C-8/10), 37.49 (C-8/10), 36.40 (C-16), 33.64 (C-5), 32.58 (C-12), 29.63 (C-13), 12.81 (C-11) ppm. **HRMS** (ESI) m/z calcd for $C_{23}H_{29}O_4N^{79}Br_2Na$ $[M + Na]^+$ 564.0356; found 564.0352.

Supplementary figure S5 and characterization of side-products S3 and S4

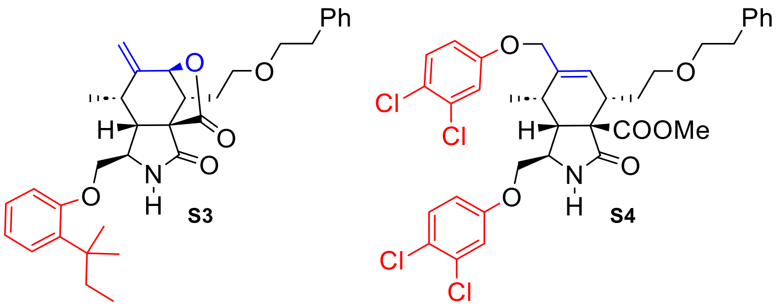

Figure S5. Side-products of Mitsunobu reaction

(1*R*,3a*S*,6*S*,8*S*,8a*R*,9*R*)-8-methyl-7-methylene-1-((2-(tert-pentyl)phenoxy)methyl)-9-(2-phenethoxyethyl)hexahydro-3H,4H-3a,6-methanooxepino[3,4-c]pyrrole-3,4-dione (S3)

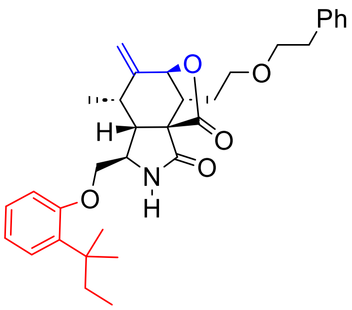

The side-product was obtained during synthesis of compound **12c**. **^{1}H NMR** (400 MHz, $CDCl_3$) δ 7.30 (m, ΣJ = 15.5 Hz, 2H, 2xH-*m*-Ph), 7.25 – 7.16 (m, 5H, H-3‘, H-5‘, H-*p*-Ph, 2xH-*o*-Ph), 6.96 (t, $J_{4',5'}$ = $J_{4',3'}$ = 7.6 Hz, 1H, H-4‘), 6.79 (d, $J_{6',5'}$ = 6.7 Hz, 1H, H-6‘), 6.06 (s, 1H, NH), 5.20 (d, J_{gem} = 2.5 Hz, 1H, H-12a), 5.13 (d, J_{gem} = 2.7 Hz, 1H, H-12b), 4.93 (d, $J_{7,8}$ = 5.5 Hz, 1H, H-7), 4.25 (dd, J_{gem} = 9.3, $J_{10a,3}$ = 2.3 Hz, 1H, H-10a), 3.98 (ddd, $J_{3,4}$ = 10.3, $J_{3,10b}$ = 9.1, $J_{3,10a}$ = 2.3 Hz, 1H, H-3), 3.85 (dd, J_{gem} = $J_{10b,3}$ = 9.1 Hz, 1H, H-10b), 3.64 (m, ΣJ = 39,2 Hz, 2H, 2xH-15), 3.46 (m, ΣJ = 42.2 Hz, 2H, 2xH-14), 3.04 (m, ΣJ = 24.4 Hz, 1H, H-5), 2.94 (ddd, $J_{8,10a/b}$ = 10.9, $J_{8,7}$ = 5.5, $J_{8,10a/b}$ = 3.1 Hz, 1H, H-8), 2.88 (dd, $J_{16,15a}$ = $J_{16,15b}$ = 7.2 Hz, 2H, 2xH-16), 2.79 (dd, $J_{4,3}$ = $J_{4,5}$ = 10.3 Hz, 1H, H-4), 1.87 – 1.73 (m, 4H, H-13a, H-13b, 2xH-18), 1.34 – 1.32 (m, 9H, 3xH-11, 3xH-20, 3xH-21), 0.63 (t, $J_{19,18}$ = 7.5 Hz, 3H, 3xH-19) ppm. **^{13}C NMR** (101 MHz, $CDCl_3$) δ 173.03 (-*C*OO-), 170.03 (C-1), 156.70 (C-1‘), 141.61 (C-6), 138.74 (C-*i*-Ph), 136.48 (C-2‘), 128.84 (C-*o*-Ph), 128.67 (C-3‘), 128.57 (C-*m*-Ph), 127.32 (C-5‘), 126.50 (C-*p*-Ph), 121.76 (C-4‘), 116.14 (C-12), 112.38 (C-6‘), 84.39 (C-7), 72.19 (C-10/15), 72.11 (C-10/15), 68.85 (C-14), 55.77 (C-9), 54.19 (C-3), 44.99 (C-8), 42.21 (C-4), 38.58 (C-17), 36.30 (C-16), 34.05 (C-18), 28.43 (C-5/20/21), 28.37 (C-5/20/21), 28.32 (C-5/20/21), 26.07 (C-13), 15.30 (C-11), 9.63 (C-19) ppm. **HRMS** (ESI) m/z calcd for $C_{33}H_{41}O_5NNa$ $[M + Na]^+$ 554.2877; found 554.2878.

Methyl (1*R*,3a*R*,4*S*,7*S*,7a*R*)-1,6-bis((3,4-dichlorophenoxy)methyl)-7-methyl-3-oxo-4-(2-phenethoxyethyl)-1,2,3,4,7,7a-hexahydro-3aH-isoindole-3a-carboxylate (S4)

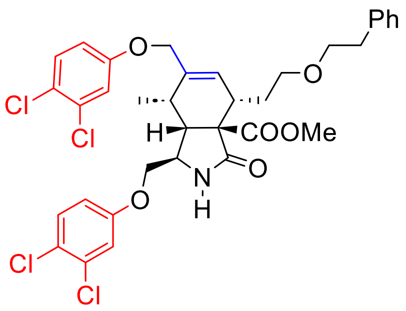

The side-product was obtained during synthesis of compound **12g**. **^{1}H NMR** (400 MHz, $CDCl_3$) δ 7.36 – 7.31 (m, 2H, H-5',11'), 7.29 – 7.16 (m, 5H, H-*p*-Ph, 2xH-*o*-Ph, 2xH-*m*-Ph), 6.98 – 6.96 (m, 2H, H-2',8'), 6.74 (dd, $J_{6',5'/12',11'}$ = 8.9, $J_{6',2'/12',8'}$ = 2.9 Hz, 1H, H-6'/12'), 6.70 (dd, $J_{6',5'/12',11'}$ = 8.9, $J_{6',2'/12',8'}$ = 2.9 Hz, 1H, H-6'/12'), 6.02 – 5.98 (m, 2H, H-7, NH), 4.44 (d, J_{gem} = 10.9 Hz, 1H, H-12a), 4.36 (d, J_{gem} = 11.0 Hz, 1H, H-12b), 3.91 (dd, J_{gem} = 8.6, $J_{10a,3}$ = 3.5 Hz, 1H, H-10a), 3.82 – 3.76 (m, 4H, H-10b, OCH_3), 3.71 – 3.66 (m, 2H, H-3,15a), 3.63 – 3.51 (m, 3H, H-14a,b,15b), 2.89 – 2.83 (m, 3H, H-8, 2xH-16), 2.64 (m, ΣJ = 30.2 Hz, 1H, H-5), 2.53 (dd, $J_{4,5}$ = $J_{4,3}$ = 4.4 Hz, 1H, H-4), 2.18 (ddd, J_{gem} = 14.0, $J_{13a,14a/14b/8}$ = 6.9, $J_{13a,14a/14b/8}$ = 2.5 Hz, 1H, H-13a), 2.03 (m, ΣJ = 39.7 Hz, 1H, H-13b), 1.27 (d, $J_{11,5}$ = 7.3 Hz, 3H, 3xH-11) ppm. **^{13}C NMR** (101 MHz, $CDCl_3$) δ 173.24 (C-1), 172.42 (*C*$OOCH_3$), 157.19 (C-1'/7'), 156.99 (C-1'/7'), 139.28 (C-*i*-Ph), 136.75 (C-6), 134.78 (C-7), 133.11 (C-3'/9'), 132.96 (C-3'/9'), 130.92 (C-5'/11'), 130.77 (C-5'/11'), 128.96 (C-*o*-Ph), 128.22 (C-*m*-Ph), 126.01 (C-*p*-Ph), 124.99 (C-4'/10'), 124.44 (C-4'/10'), 116.53 (C-2'/8'), 116.19 (C-2'/8'), 114.58 (C-6'/12'), 114.53 (C-6'/12'), 72.50 (C-10), 71.37 (C-15), 69.76 (C-14), 69.41 (C-12), 59.71 (C-9), 53.29 (C-3), 53.03 (COO*C*H_3), 50.36 (C-4), 37.22 (C-8), 36.32 (C-16), 33.42 (C-5), 29.69 (C-13), 13.21 (C-11) ppm. **HRMS** (ESI) m/z calcd for $C_{35}H_{35}O_6NCl_4Na$ $[M + Na]^+$ 728.1111; found 728.1115.

HPLC purity of final cytochalasan analogues

Separation conditions

RP Prime column (3 µm, 120 Å, 150 × 3.0 mm I.D.), mobile phase: MeCN/H_2O (7/3), and alternatively* (1:1), flow rate: 0.5 mL/min. Analytes dissolved in methanol at a concentration of 1-2 mg/mL.

Table S3. Purity of final cytochalasan analogues.

Sample	Retention time [min]	Purity [%]
**8a**	4.23	99.9
**8b**	12.78	99.2
**8c**	8.64	99.9
**11***	4.14	99.9
**12a**	3.46	99.9
**12b**	7.73	99.9
**12c**	4.64	99.9
**12d**	3.68	99.1
**12e**	3.07	98.3
**12f**	4.34	98.5
**12g**	4.96	98.1

HPLC traces of final cytochalasan analogues

8a - purity 99.9, T_R = 4.23 min.

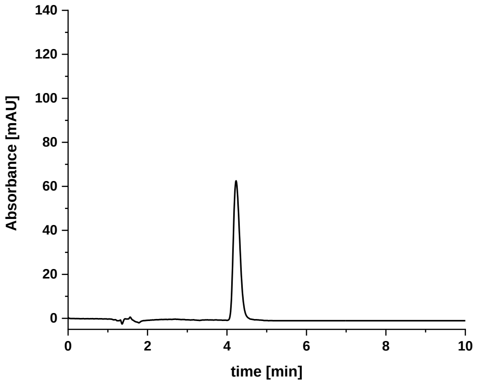

8b - purity 99.2, T_R = 12.78 min.

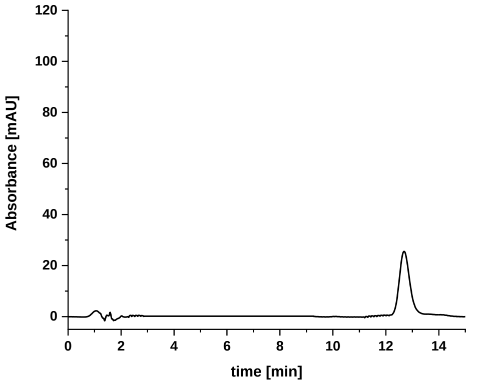

8c - purity 99.9, T_R = 8.64 min.

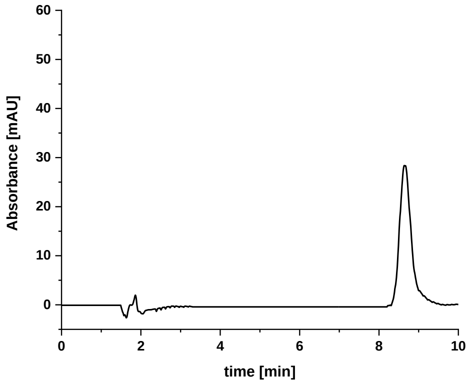

11 - purity 99.9, T_R = 4.14 min.

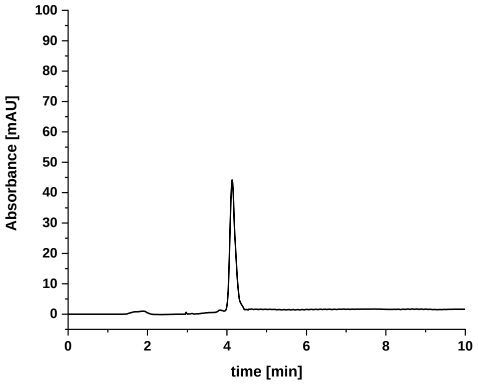

12a - purity 99.9, T_R = 3.46 min.

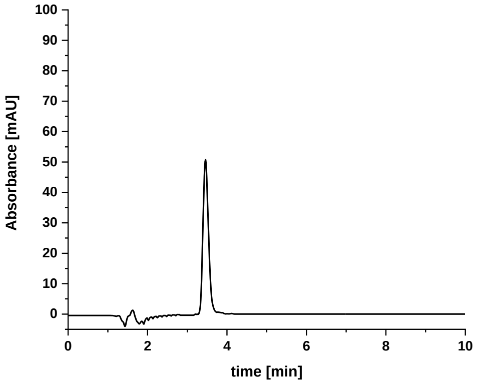

12b - purity 99.9, T_R = 7.73 min.

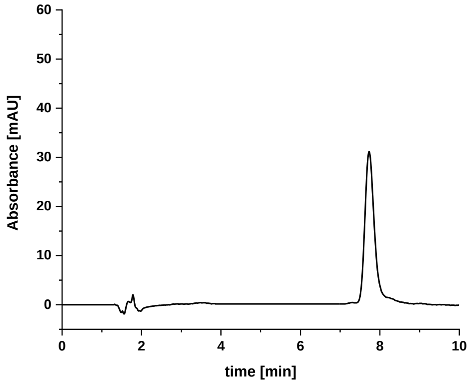

12c - purity 99.9, T_R = 4.64 min.

12d - purity 99.1, T_R = 3.68 min.

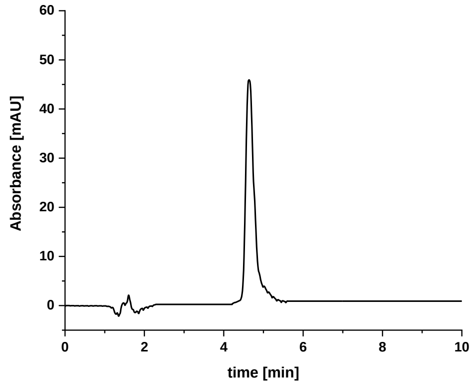

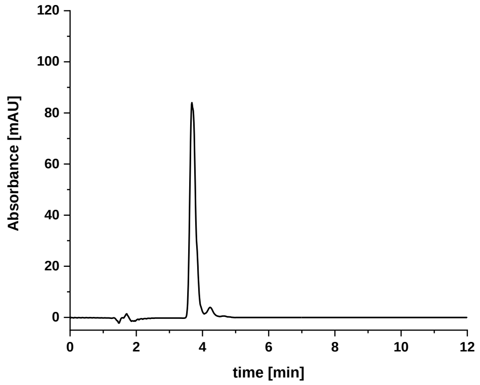

12e - purity 98.3, T_R = 3.07 min.

12f - purity 98.5, T_R = 4.34 min.

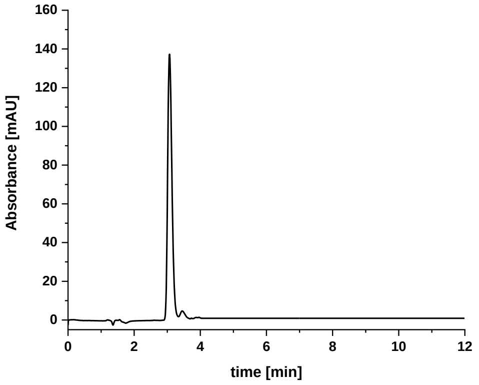

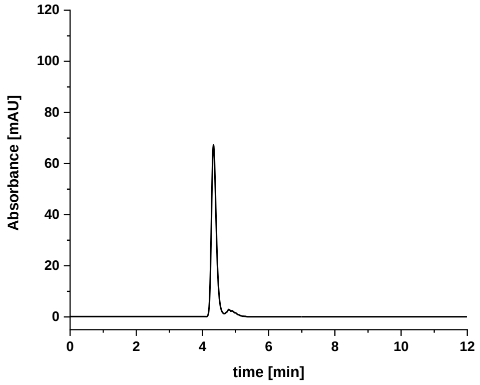

12g - purity 98.1, T_R = 4.96 min.

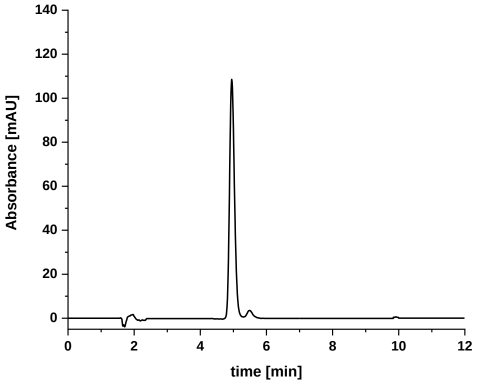

Copies of NMR spectra

Compound 7

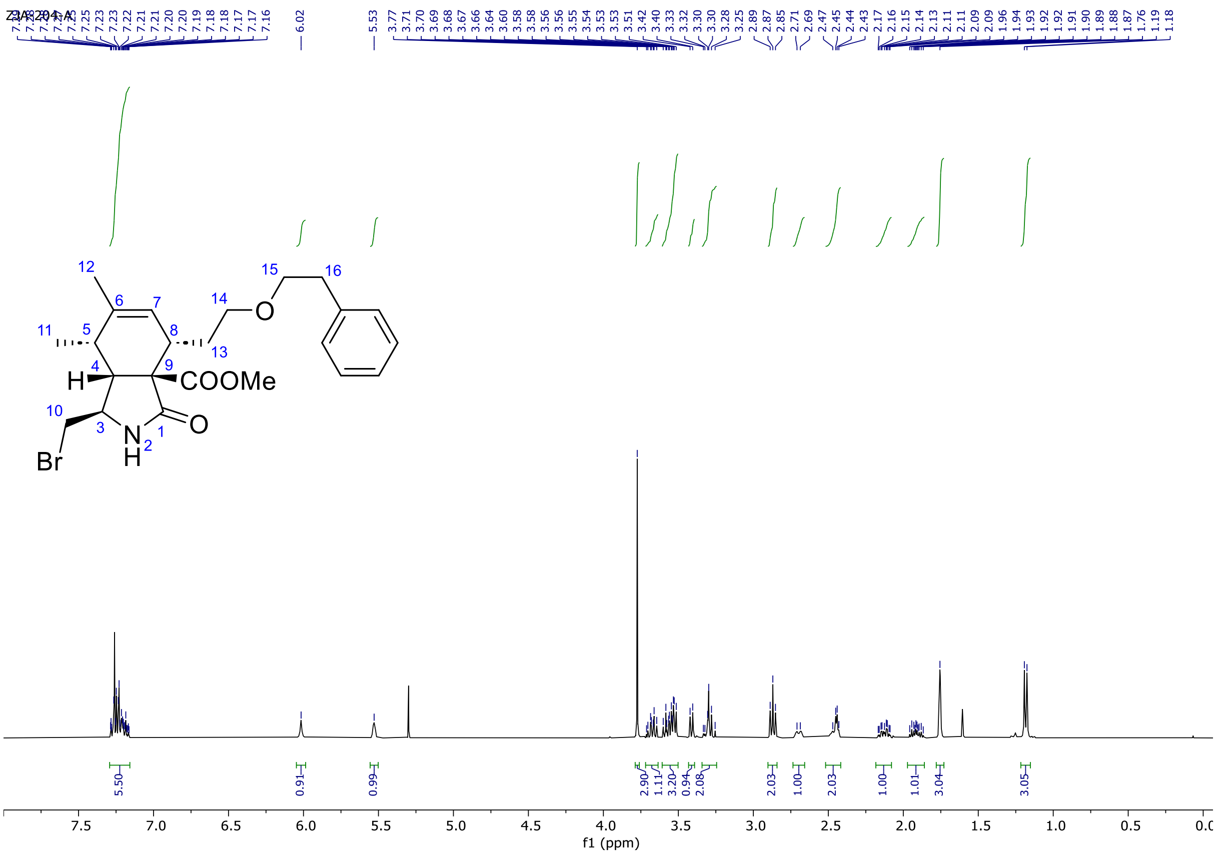

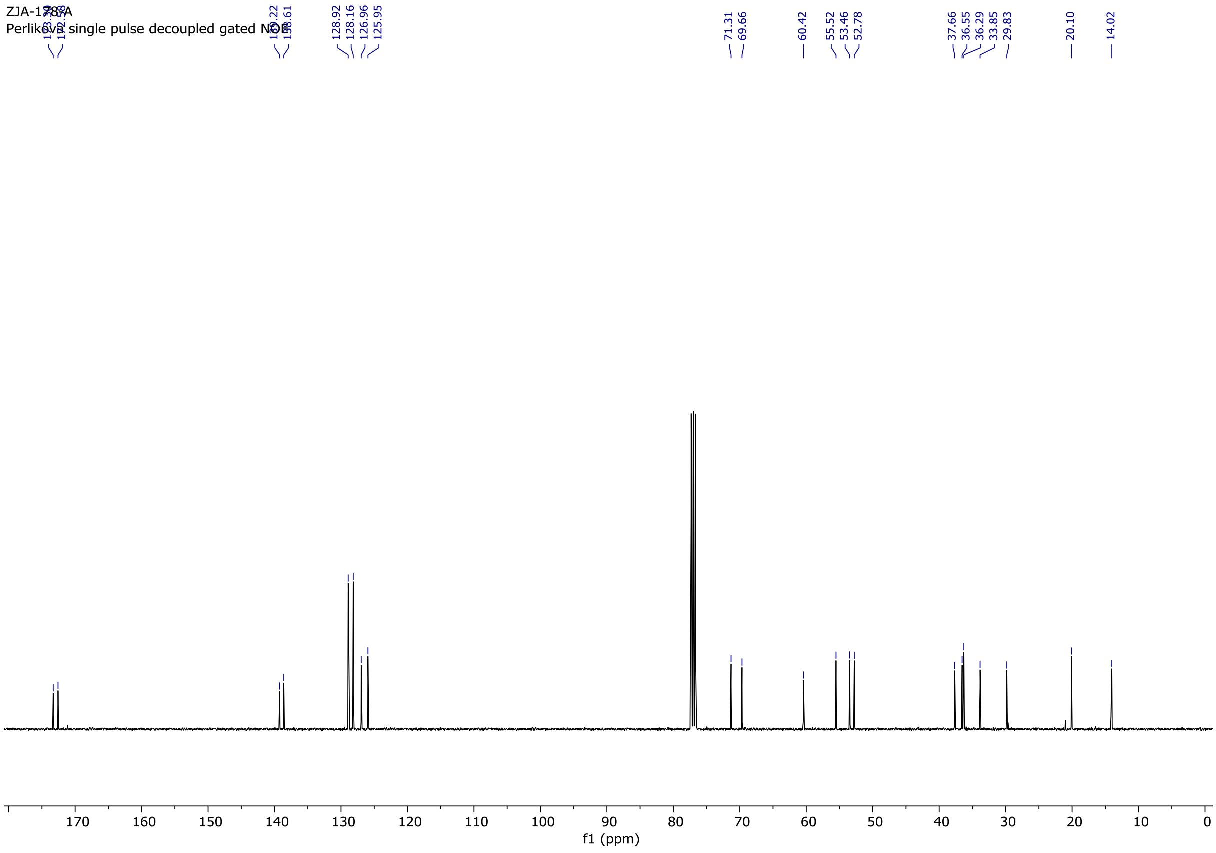
ZJA-178A
Perlikova single pulse decoupled gated NOE
138.61
128.92
128.16
126.96
125.95
71.31
69.66
60.42
55.52
53.46
52.78
37.66
36.55
36.29
33.85
29.83
20.10
14.02
170 160 150 140 130 120 110 100 90 80 70 60 50 40 30 20 10 0
f1 (ppm)

Compound 8a

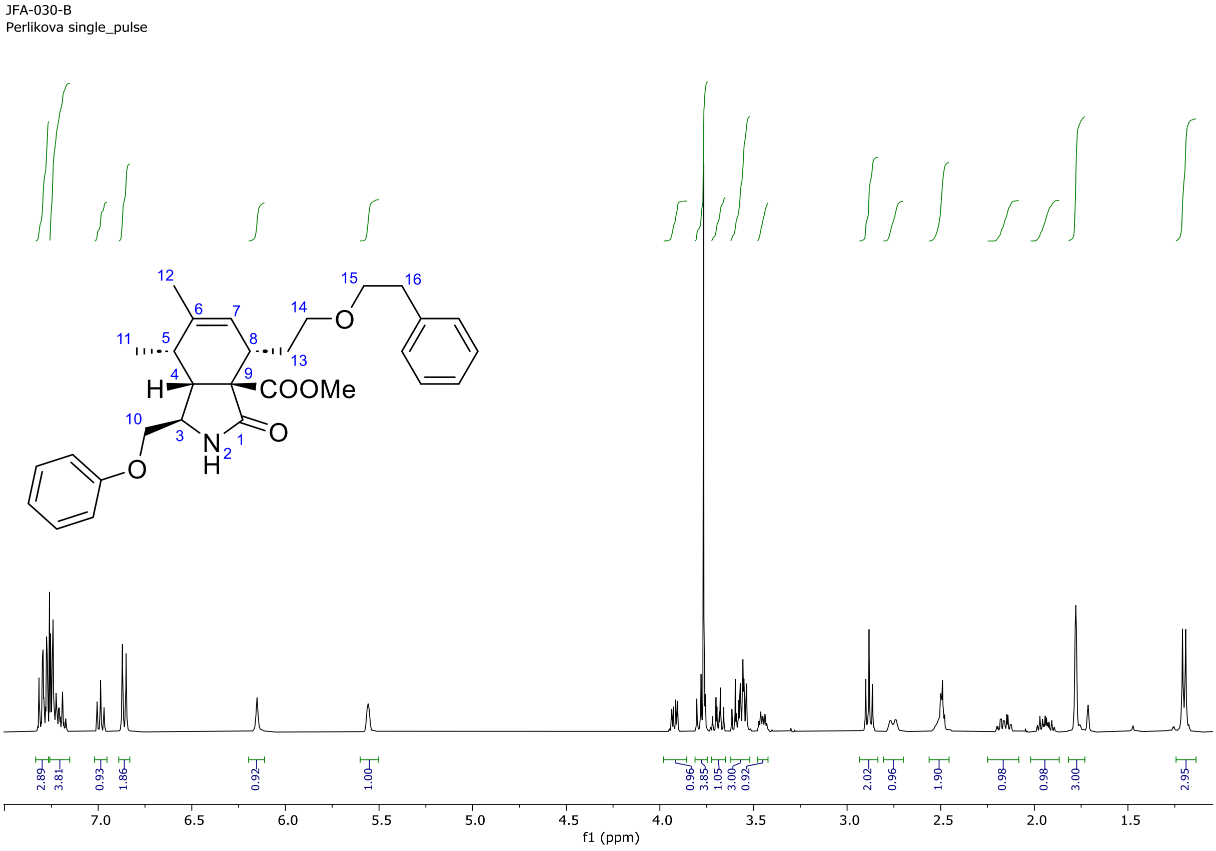

JFA-030-B
Perlikova single_pulse
12
6
7
14
15
16
11
5
8
13
O
H
4
9
COOMe
10
3
N
H
2
1
O
O
2.89
3.81
0.93
1.86
0.92
1.00
0.96
3.85
1.05
3.00
0.92
2.02
0.96
1.90
0.98
0.98
3.00
2.95
7.0
6.5
6.0
5.5
5.0
4.5
4.0
3.5
3.0
2.5
2.0
1.5
f1 (ppm)

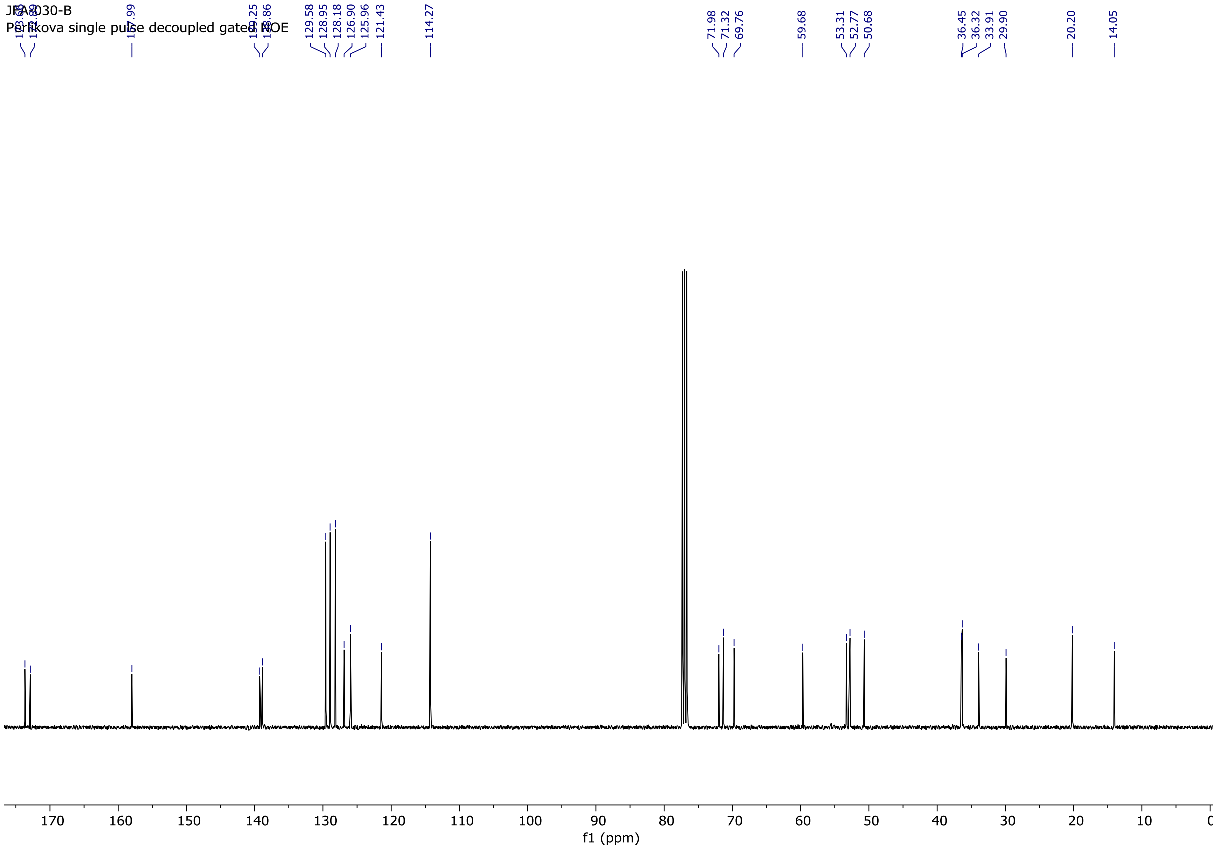

JFA-030-B
Perlikova single pulse decoupled gated NOE
129.58
128.95
128.18
126.90
125.96
121.43
114.27
71.98
71.32
69.76
59.68
53.31
52.77
50.68
36.45
36.32
33.91
29.90
20.20
14.05
170
160
150
140
130
120
110
100
90
80
70
60
50
40
30
20
10
f1 (ppm)

Compound 8b

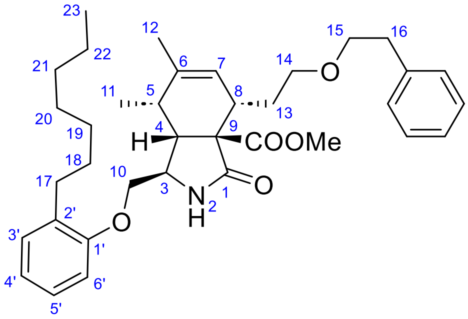
23
22
21
20
19
18
17
12
11
5
6
7
8
13
14
15
16
O
H
4
9
COOMe
10
3
N
H
2
1
O
O
2'
3'
1'
4'
5'
6'

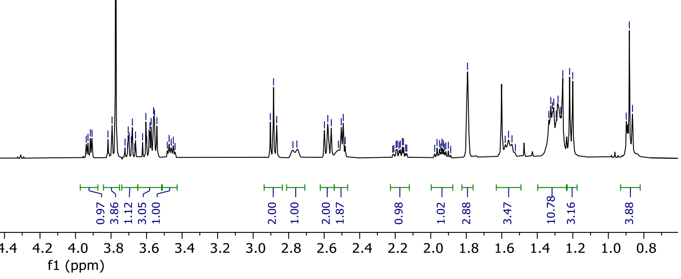

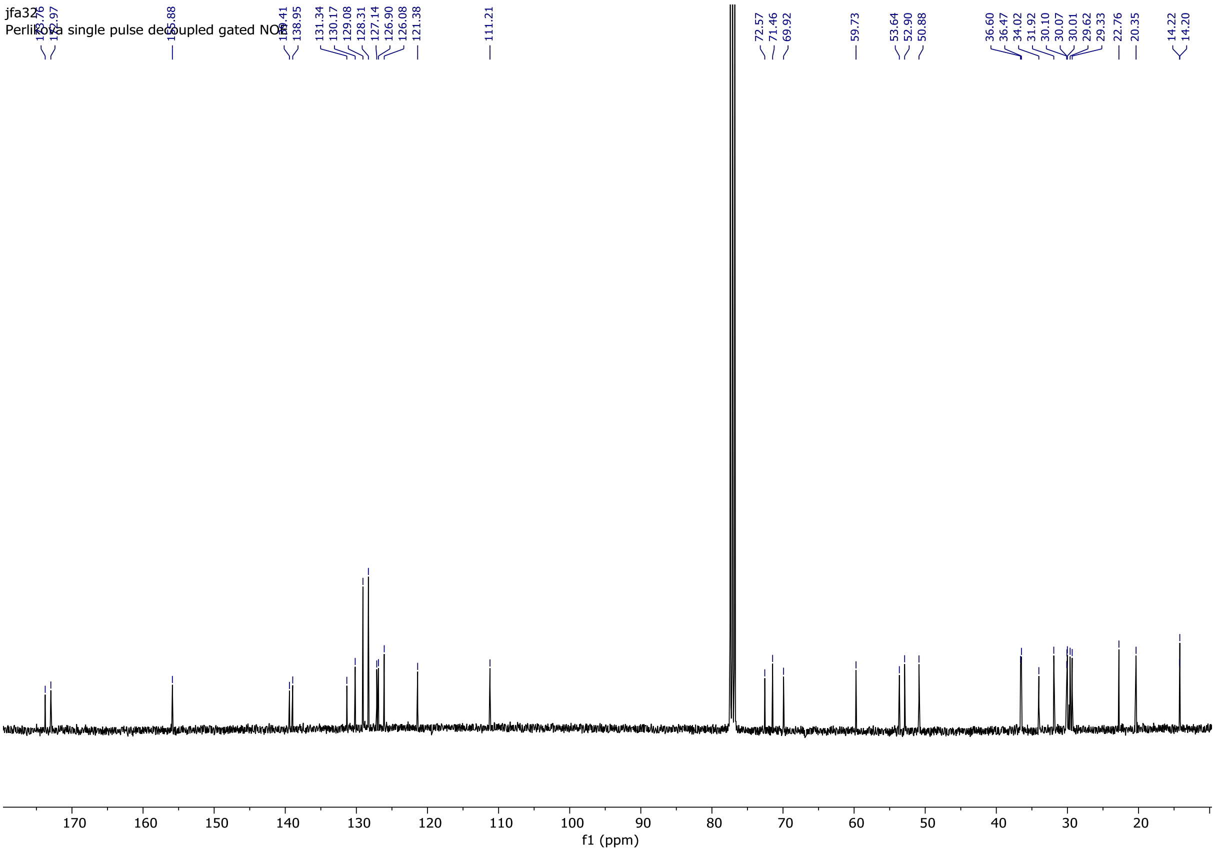

Compound 8c

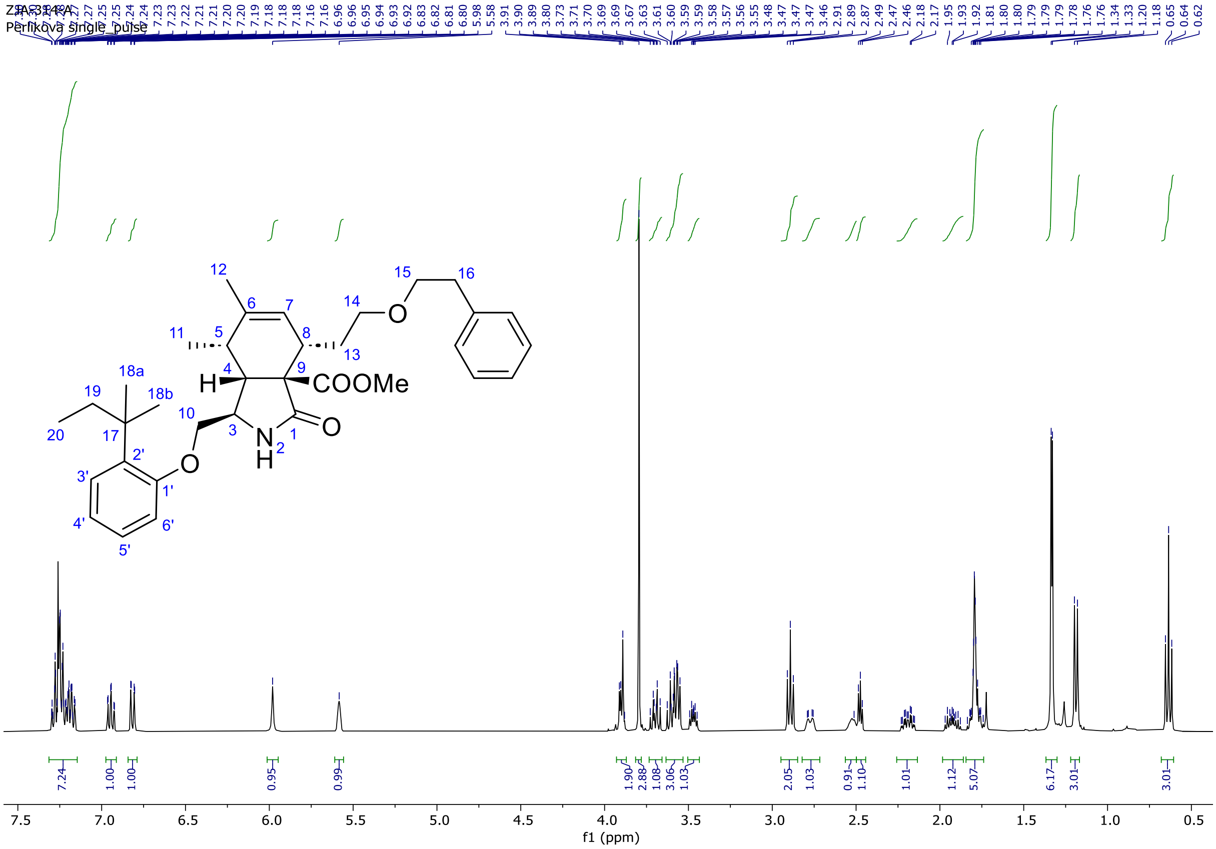
ZJA-314-A
Perlikova single pulse
7.27
7.25
7.25
7.24
7.24
7.23
7.23
7.22
7.21
7.21
7.20
7.20
7.19
7.18
7.18
7.18
7.16
7.16
6.96
6.96
6.95
6.94
6.93
6.92
6.83
6.82
6.81
6.80
5.98
5.58
3.91
3.90
3.89
3.80
3.73
3.71
3.70
3.69
3.69
3.67
3.63
3.61
3.60
3.59
3.59
3.58
3.57
3.56
3.55
3.48
3.47
3.47
3.46
2.91
2.89
2.87
2.49
2.47
2.46
2.18
2.17
1.95
1.93
1.92
1.81
1.80
1.80
1.79
1.79
1.79
1.78
1.76
1.76
1.34
1.33
1.20
1.18
0.65
0.64
0.62
12
6
7
15
16
14
O
11
5
8
13
18a
H
4
9
COOMe
19
18b
10
3
N
1
O
20
17
H
2
2'
O
3'
1'
4'
6'
5'
7.24
1.00
1.00
0.95
0.99
1.90
2.88
1.08
3.06
1.03
2.05
1.03
0.91
1.10
1.01
1.12
5.07
6.17
3.01
3.01
7.5
7.0
6.5
6.0
5.5
5.0
4.5
4.0
3.5
3.0
2.5
2.0
1.5
1.0
0.5
f1 (ppm)

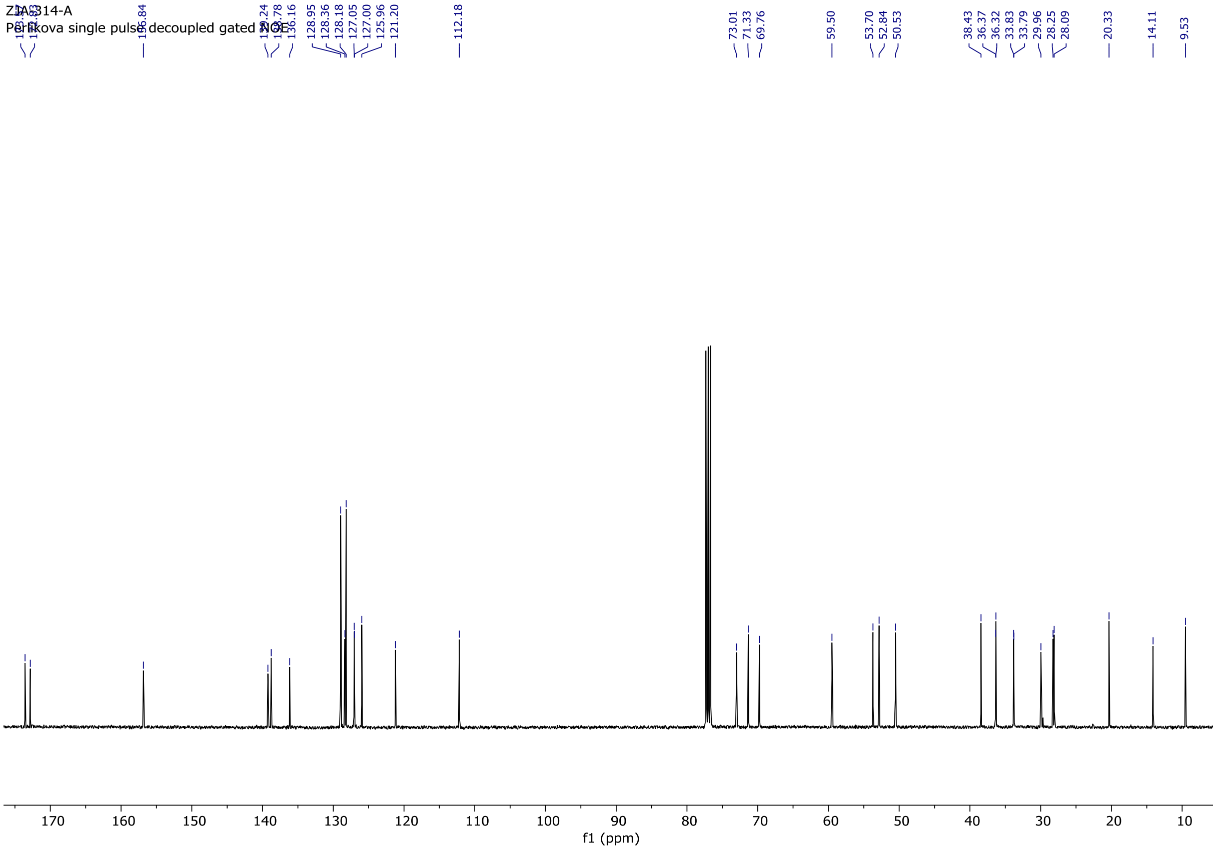
ZJA-314-A
Perlikova single pulse decoupled gated NOE
[illegible]
[illegible]
156.84
139.24
138.78
136.16
128.95
128.36
128.18
127.05
127.00
125.96
121.20
112.18
73.01
71.33
69.76
59.50
53.70
52.84
50.53
38.43
36.37
36.32
33.83
33.79
29.96
28.25
28.09
20.33
14.11
9.53
170
160
150
140
130
120
110
100
90
80
70
60
50
40
30
20
10
f1 (ppm)

Compound 9a

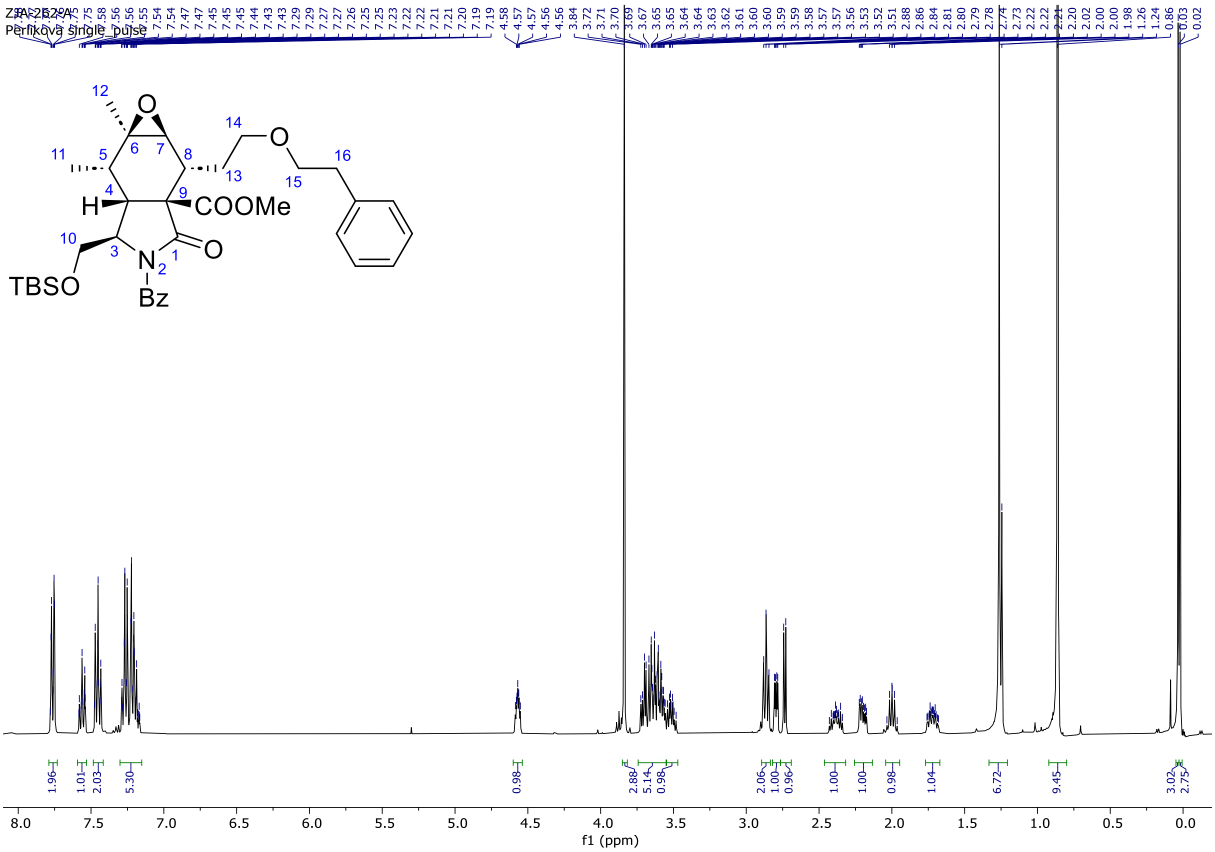
ZJA-262-A
Perlikova single_pulse
TBSO
Bz
COOMe
f1 (ppm)

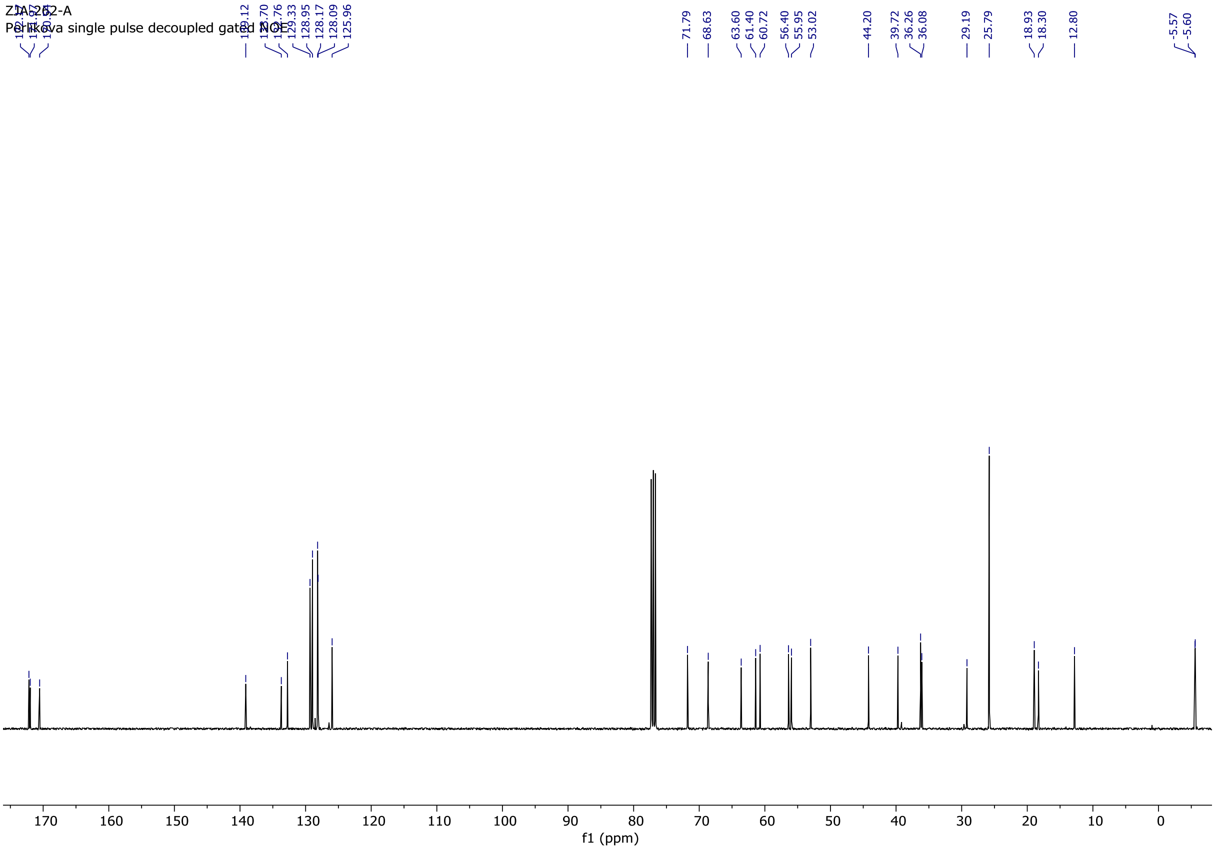
ZJA-262-A
Perlikova single pulse decoupled gated NOE
f1 (ppm)

Compound 9b

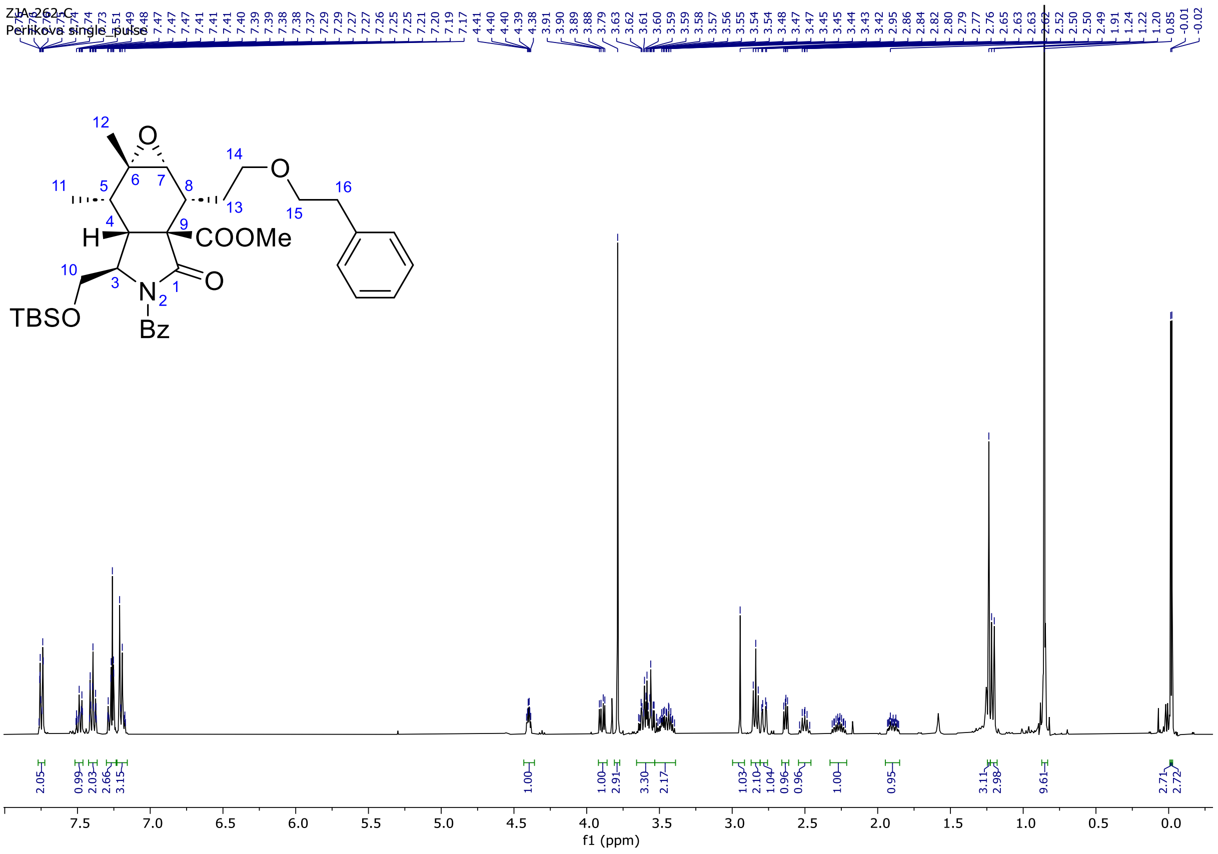
ZJA-262-C
Perlikova single_pulse
TBSO
Bz
COOMe
f1 (ppm)

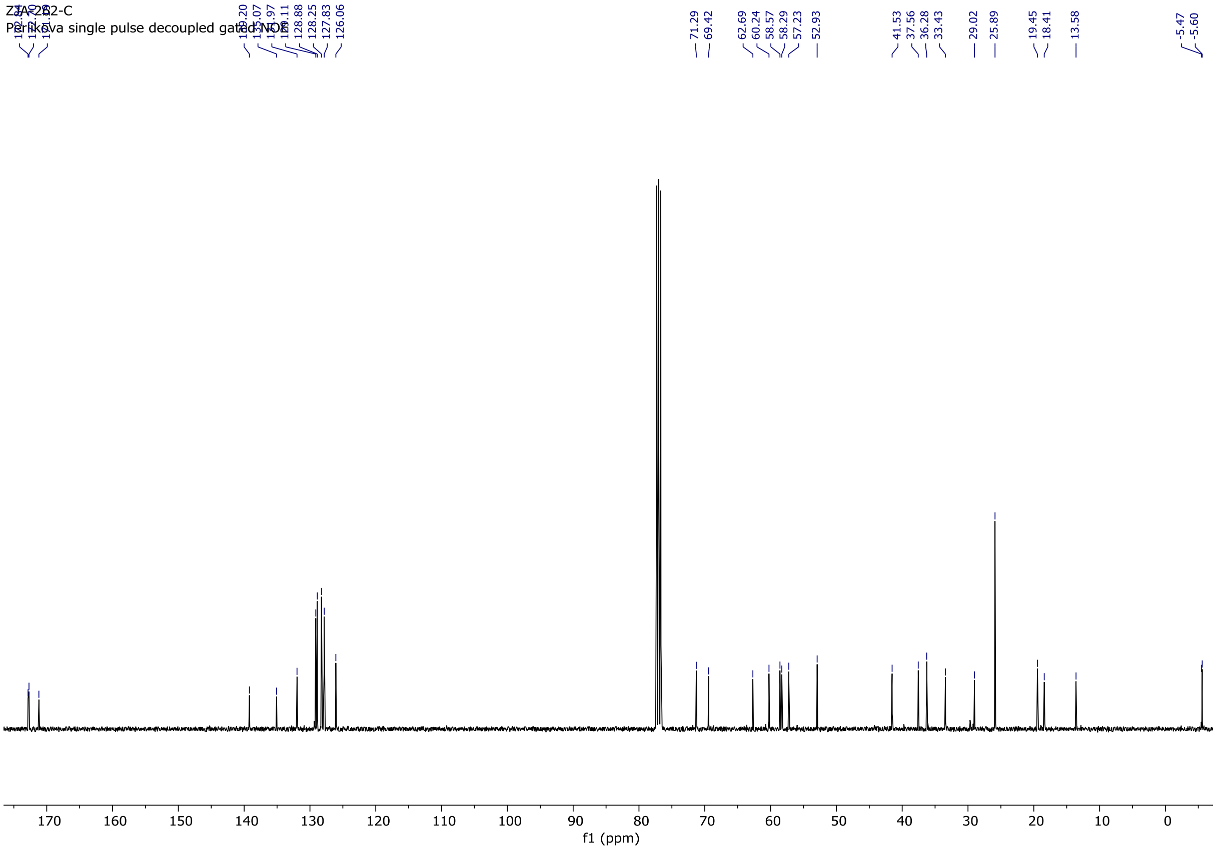
ZJA-262-C
Perlikova single pulse decoupled gated NOE
f1 (ppm)

Compound 10

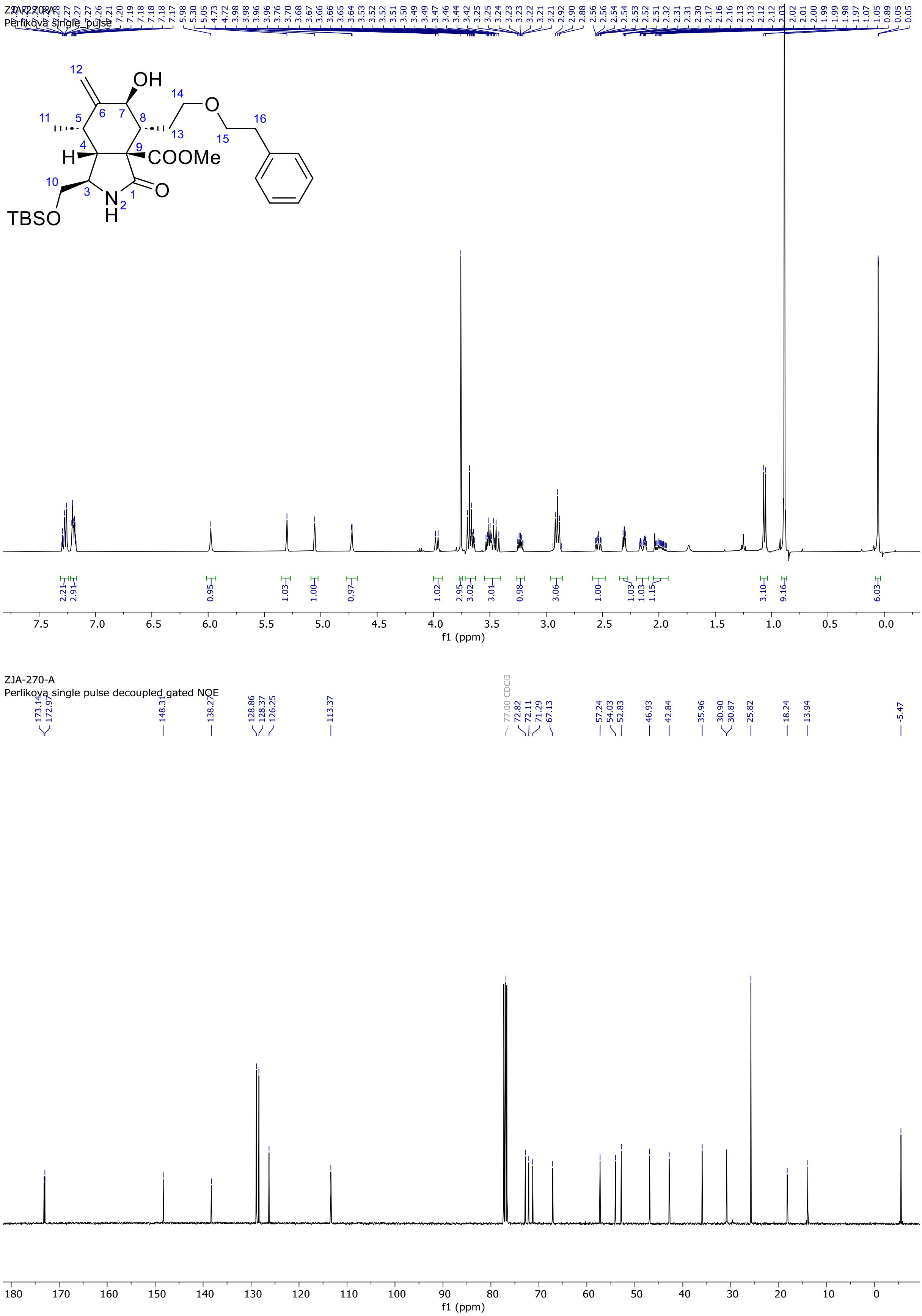
ZJA-270-A
Perlikova single_pulse
OH
COOMe
TBSO
f1 (ppm)
ZJA-270-A
Perlikova single pulse decoupled gated NOE
173.14
172.97
148.31
138.27
128.86
128.37
126.25
113.37
77.00 CDCl3
72.82
72.11
71.29
67.13
57.24
54.03
52.83
46.93
42.84
35.96
30.90
30.87
25.82
18.24
13.94
-5.47
f1 (ppm)

Compound 11

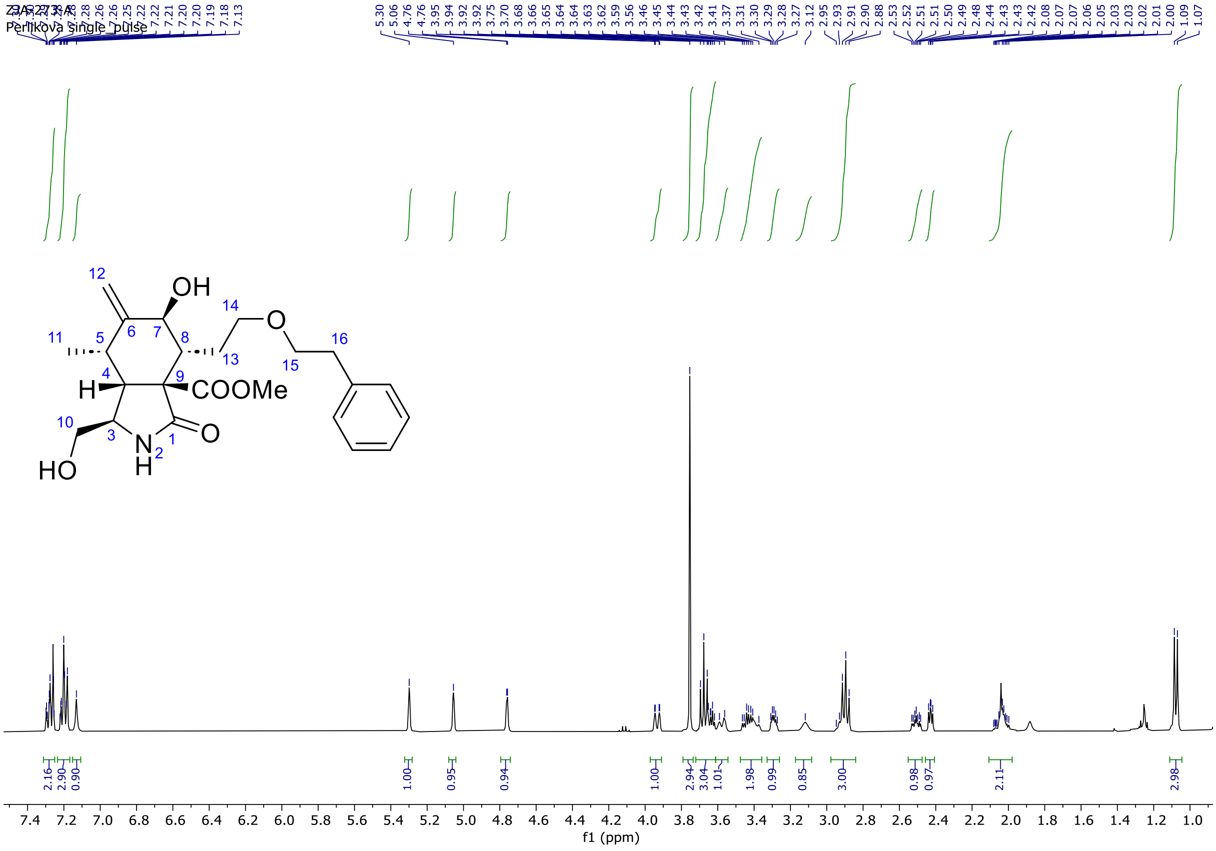

ZJA-273-A
Perlikova single pulse
5.30 5.06 4.76 3.94 3.92 3.75 3.70 3.68 3.66 3.65 3.64 3.63 3.62 3.59 3.56 3.46 3.45 3.44 3.43 3.42 3.41 3.37 3.31 3.30 3.29 3.28 3.27 3.12 2.95 2.93 2.91 2.90 2.88 2.53 2.52 2.51 2.50 2.49 2.48 2.44 2.43 2.42 2.08 2.07 2.06 2.05 2.03 2.02 2.01 2.00 1.09 1.07
12 OH 14 O 16 11 5 6 7 8 13 15 4 9 H COOMe 10 3 N 1 O 2 HO H
2.16 2.90 0.90 1.00 0.95 0.94 1.00 2.94 3.04 1.01 1.98 0.99 0.85 3.00 0.98 0.97 2.11 2.98
f1 (ppm)

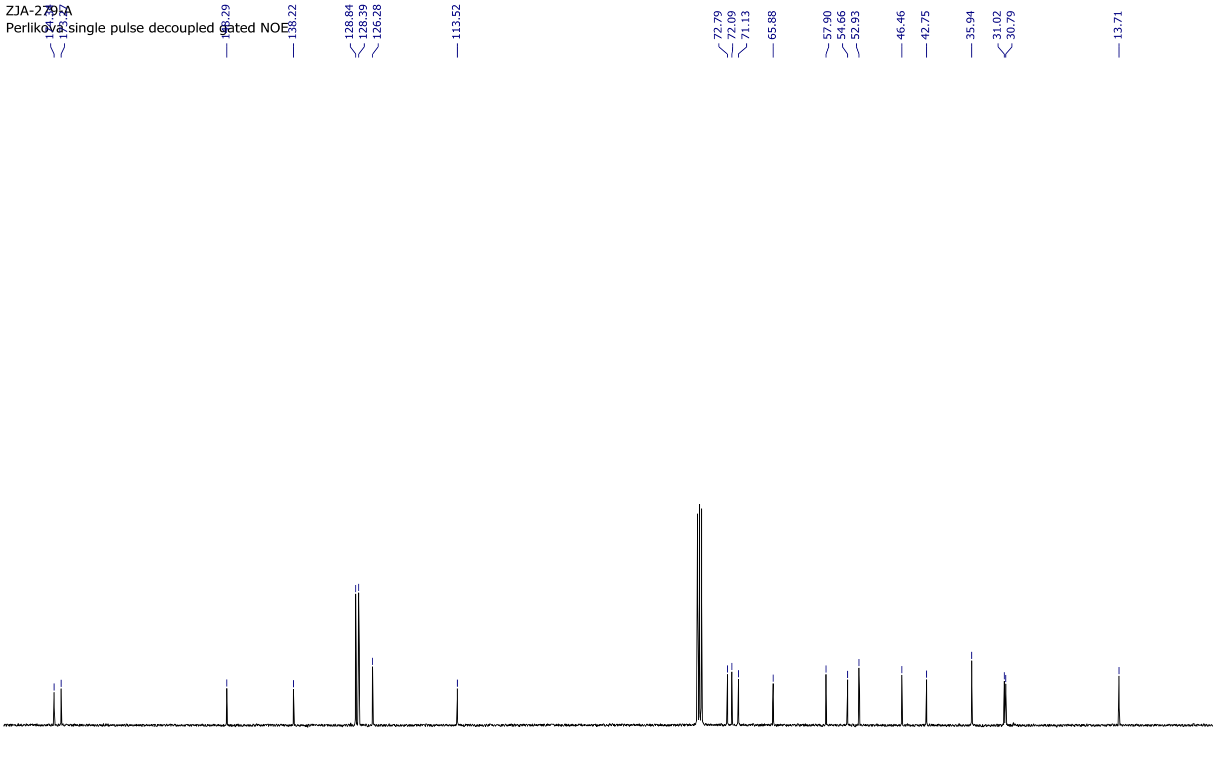

ZJA-279-A
Perlikova single pulse decoupled gated NOE
138.22 128.84 128.39 126.28 113.52 72.79 72.09 71.13 65.88 57.90 54.66 52.93 46.46 42.75 35.94 31.02 30.79 13.71
f1 (ppm)

Compound 12a

ZJA-367-B
Perlikova single_pulse

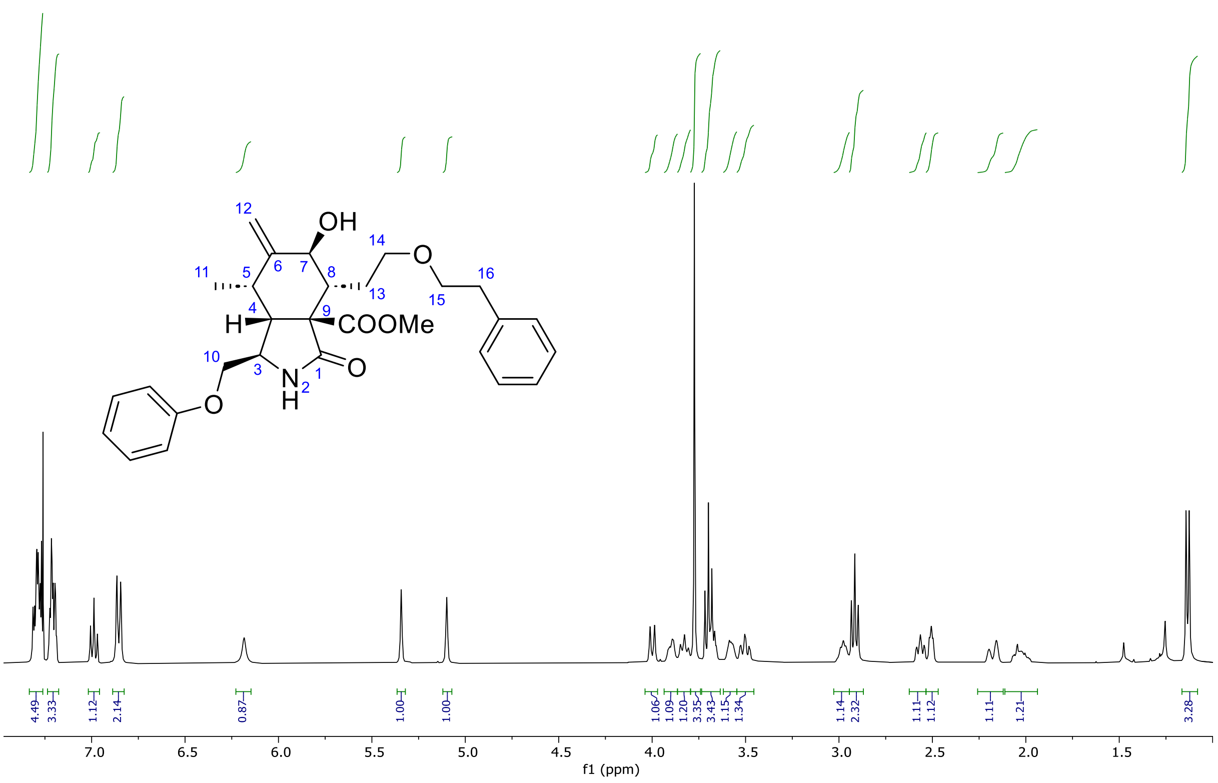

ZJA-367-B
Perlikova single pulse decoupled gated NOE

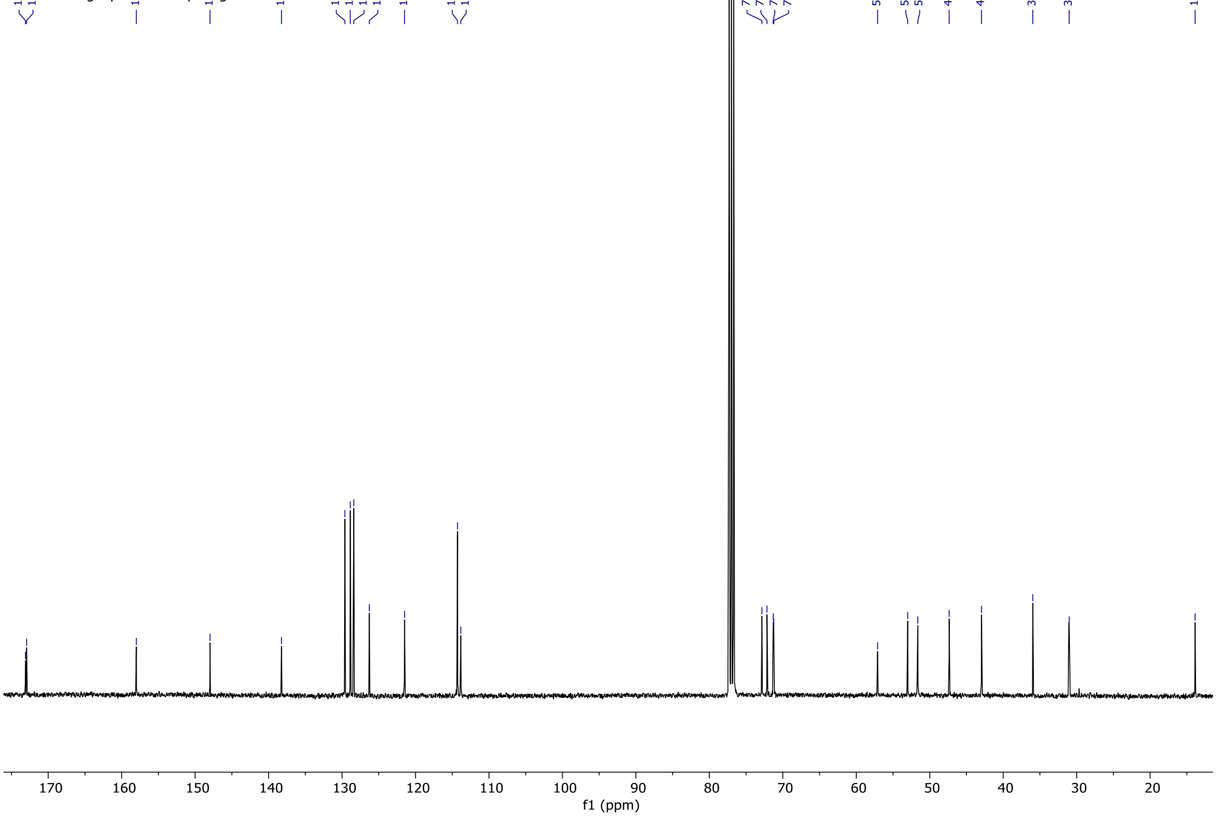

Compound 12b

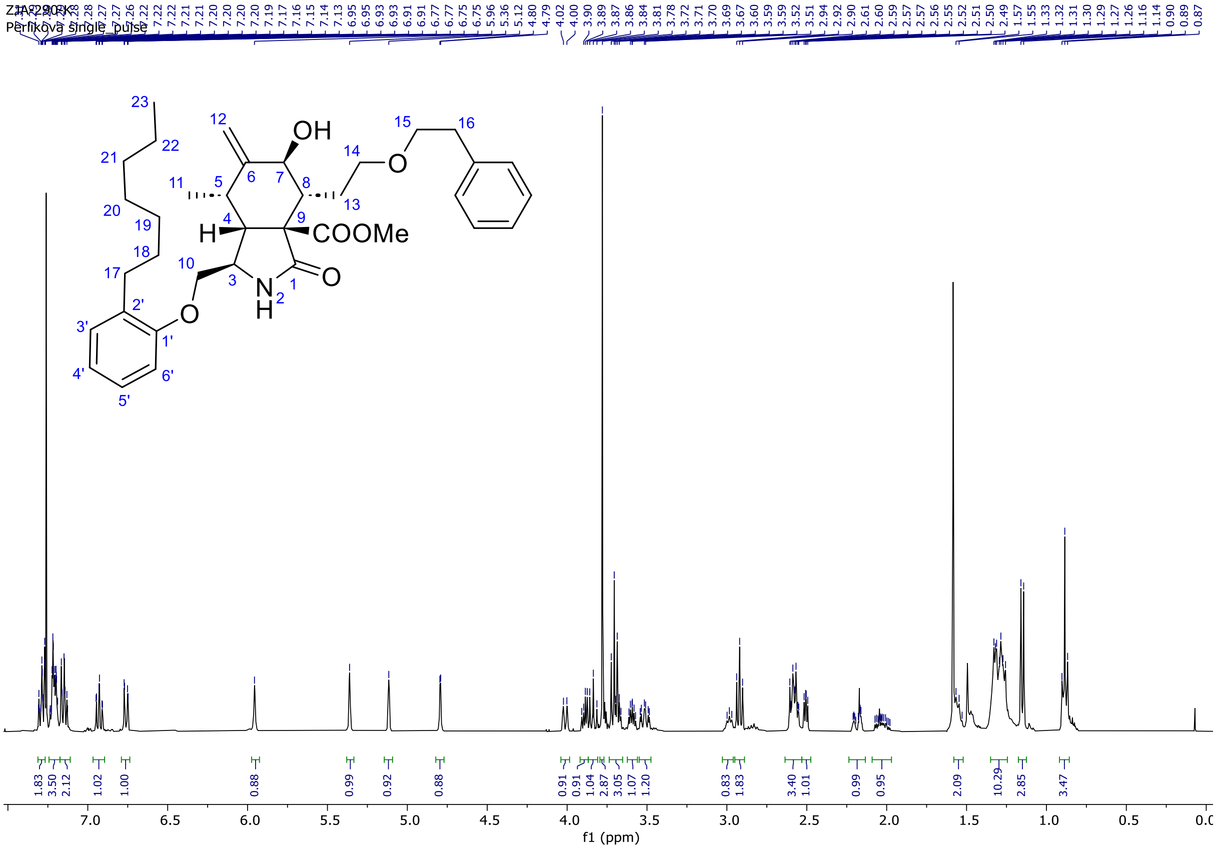

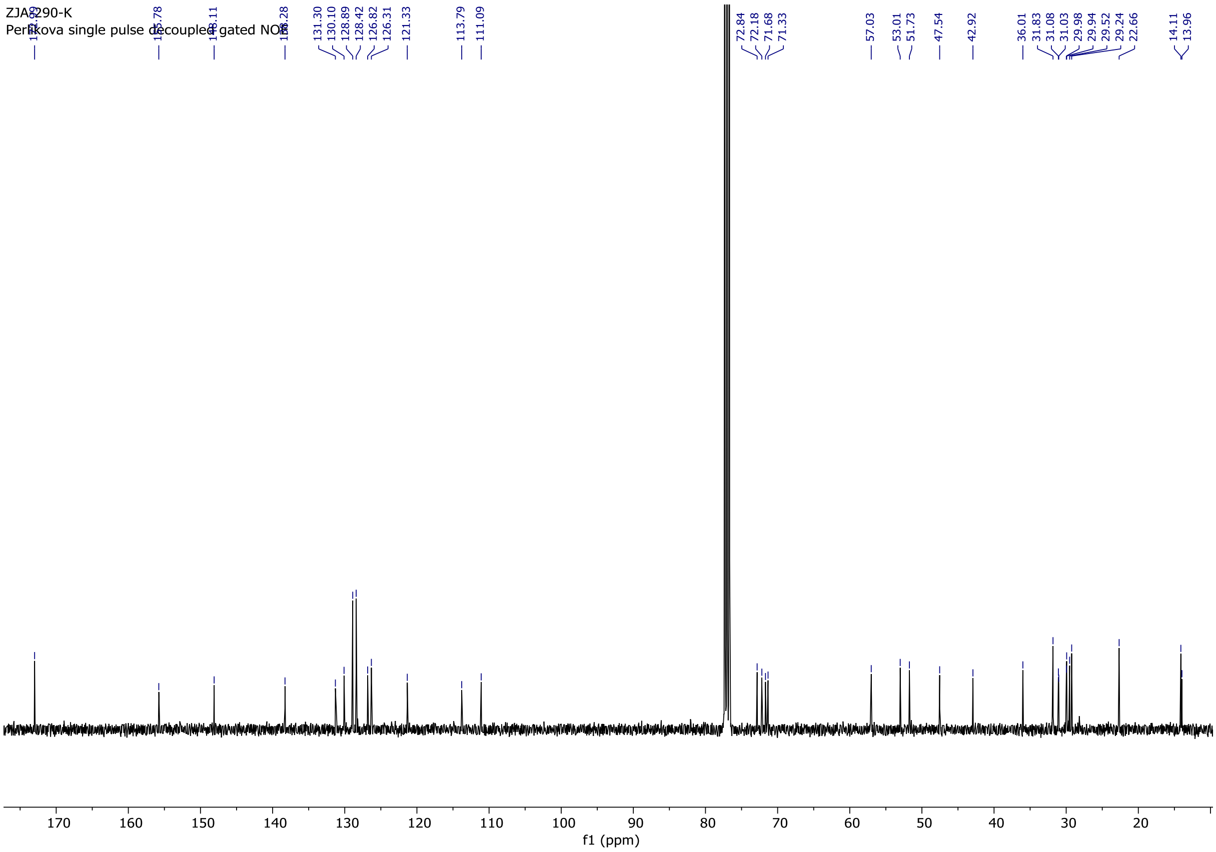

Compound 12c

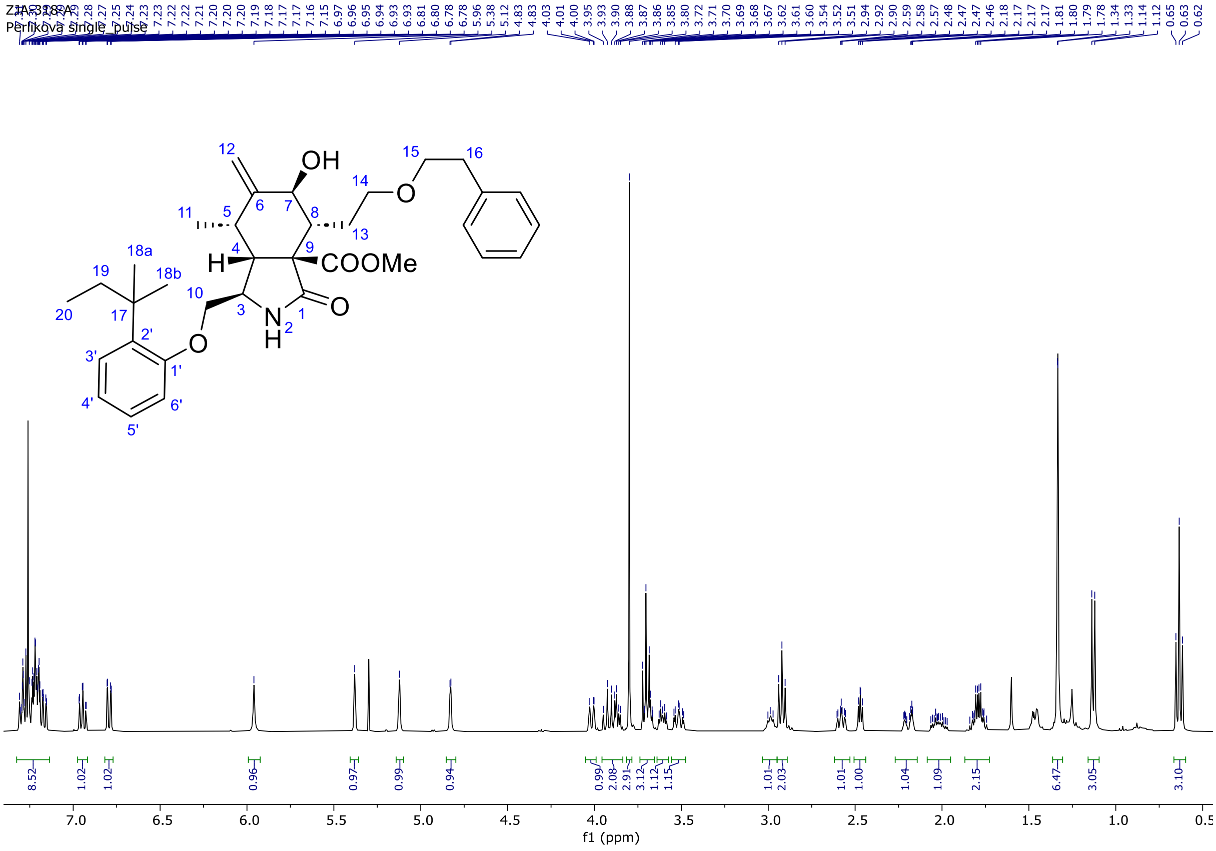

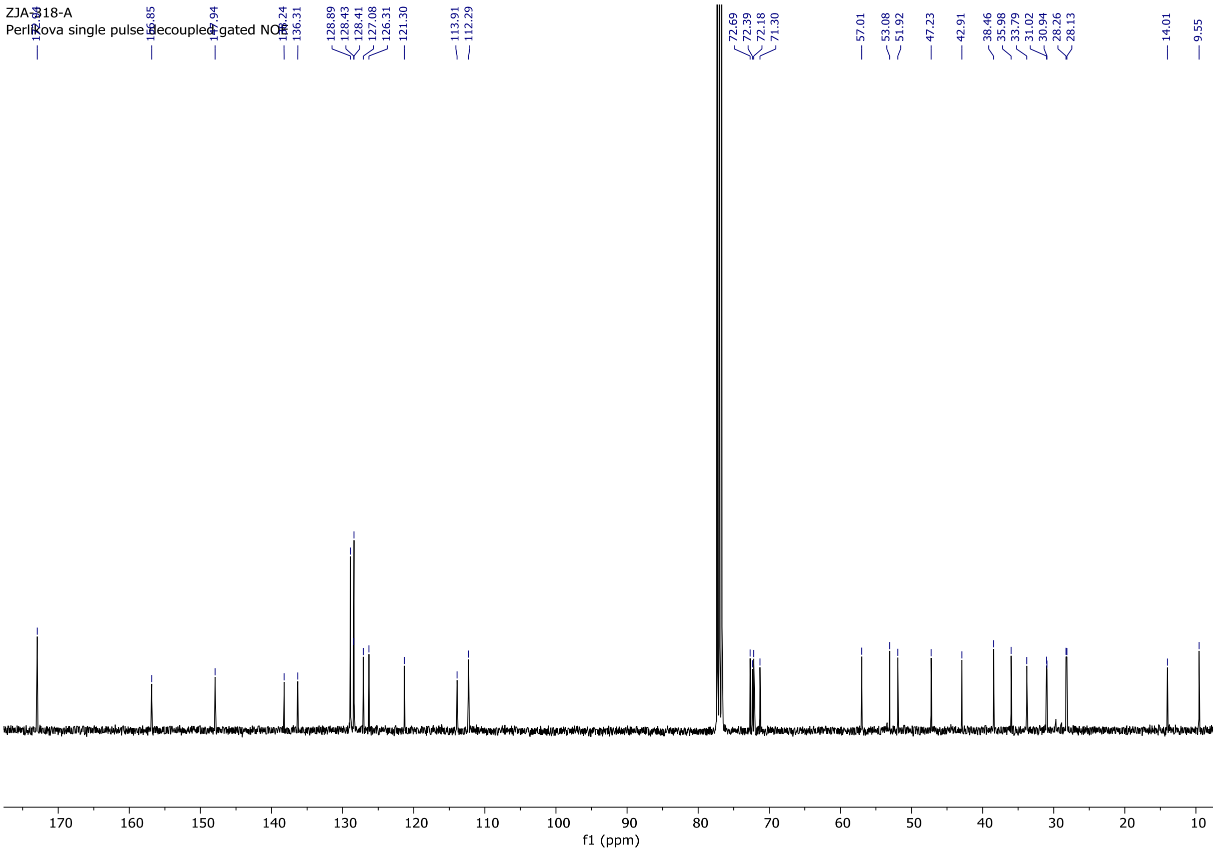

Compound 12d

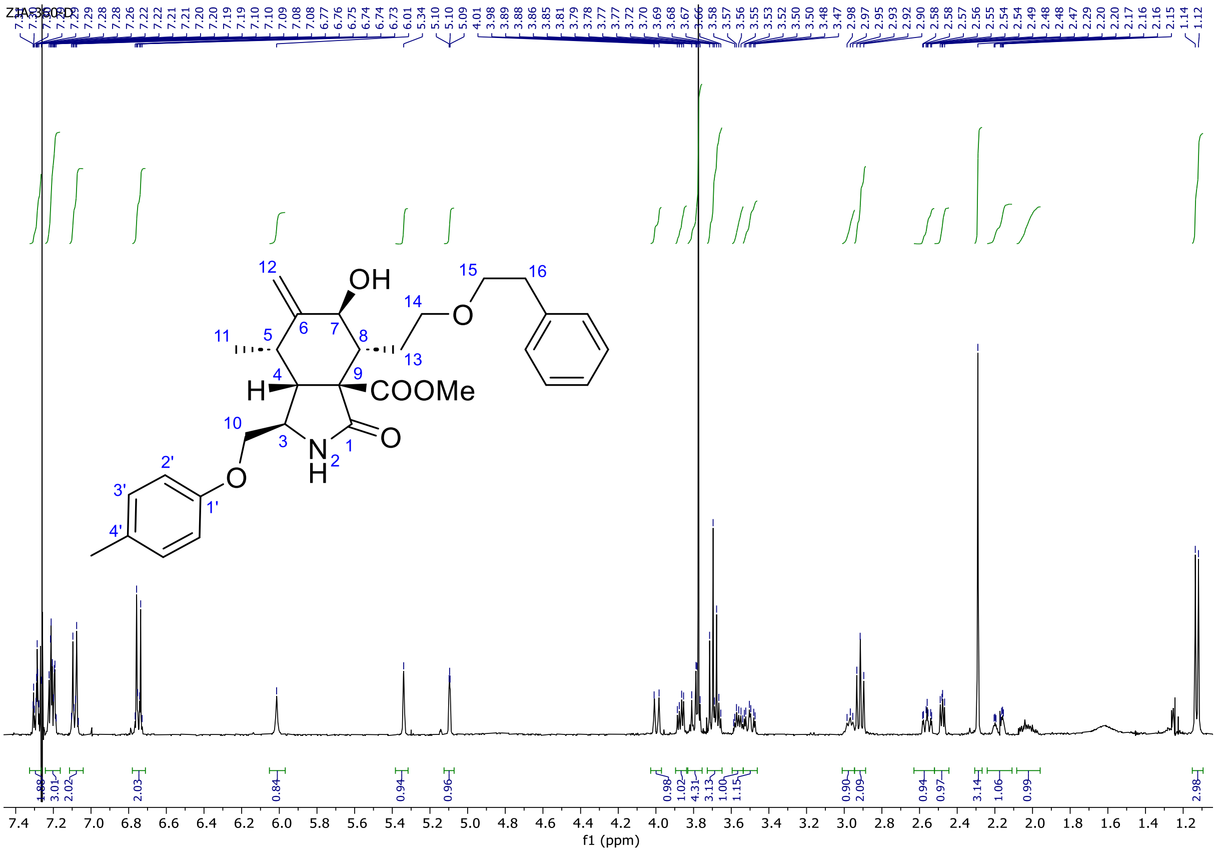

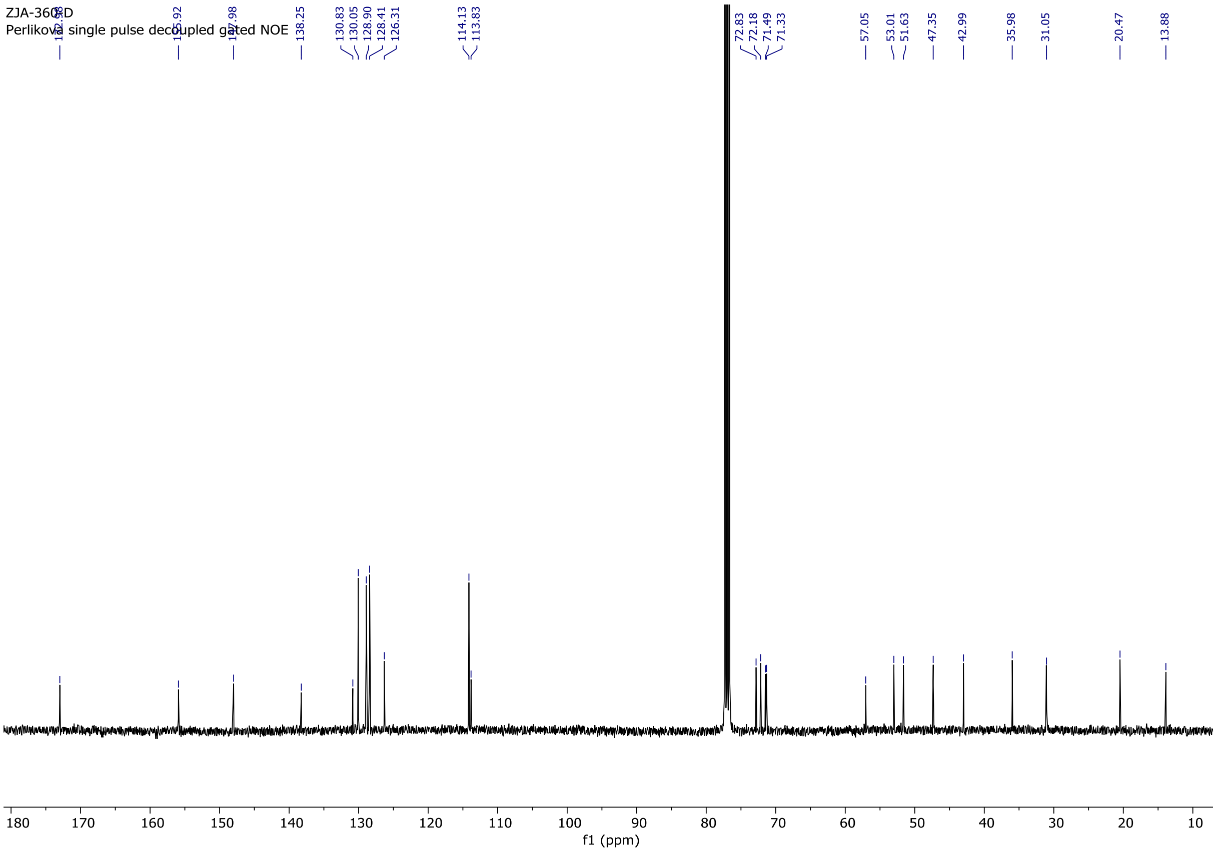

Compound 12e

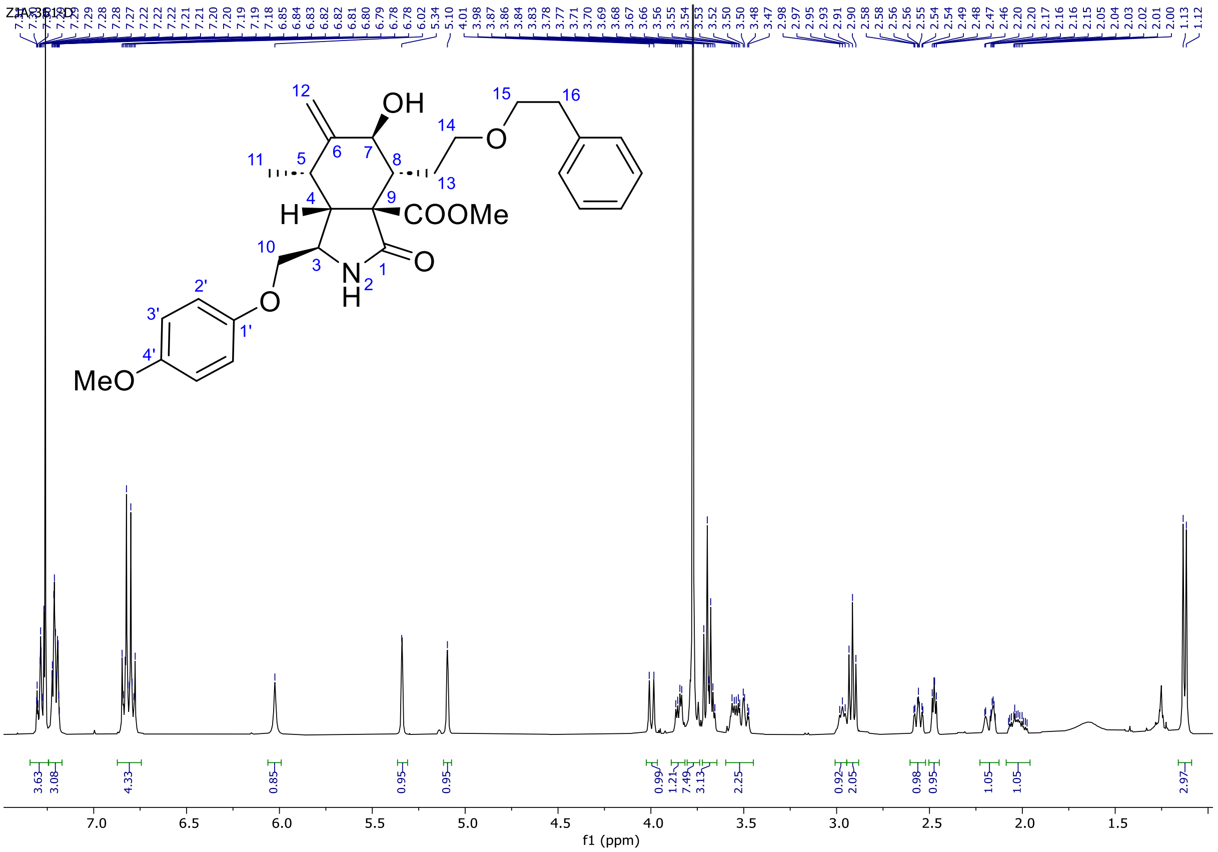

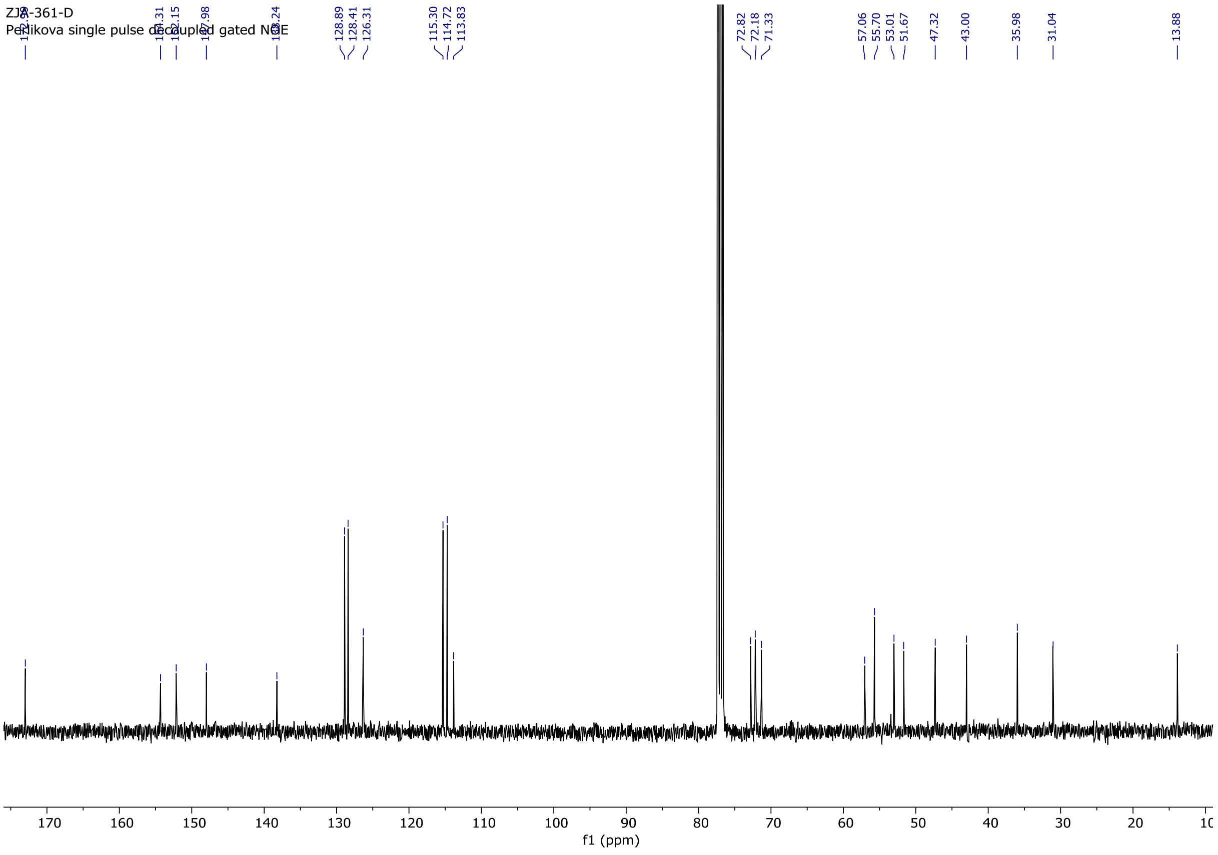

Compound 12f

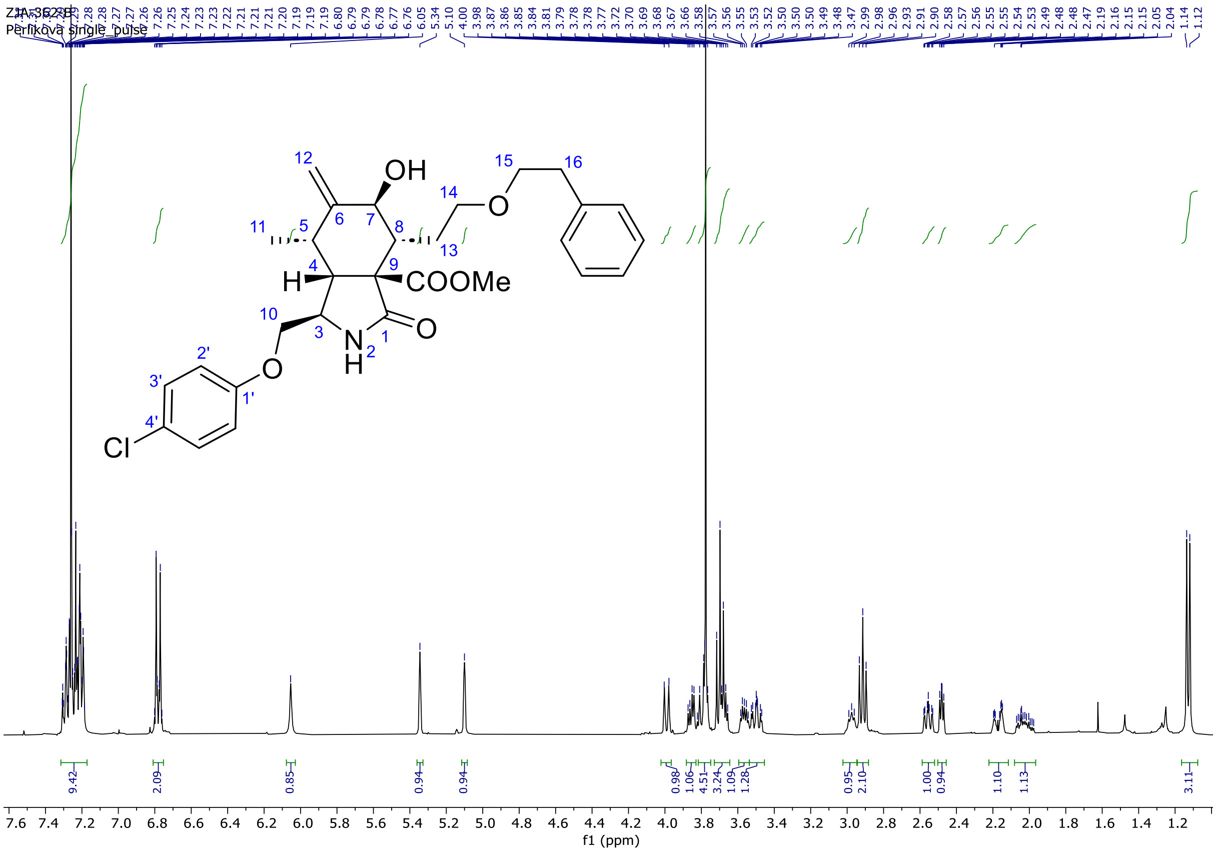

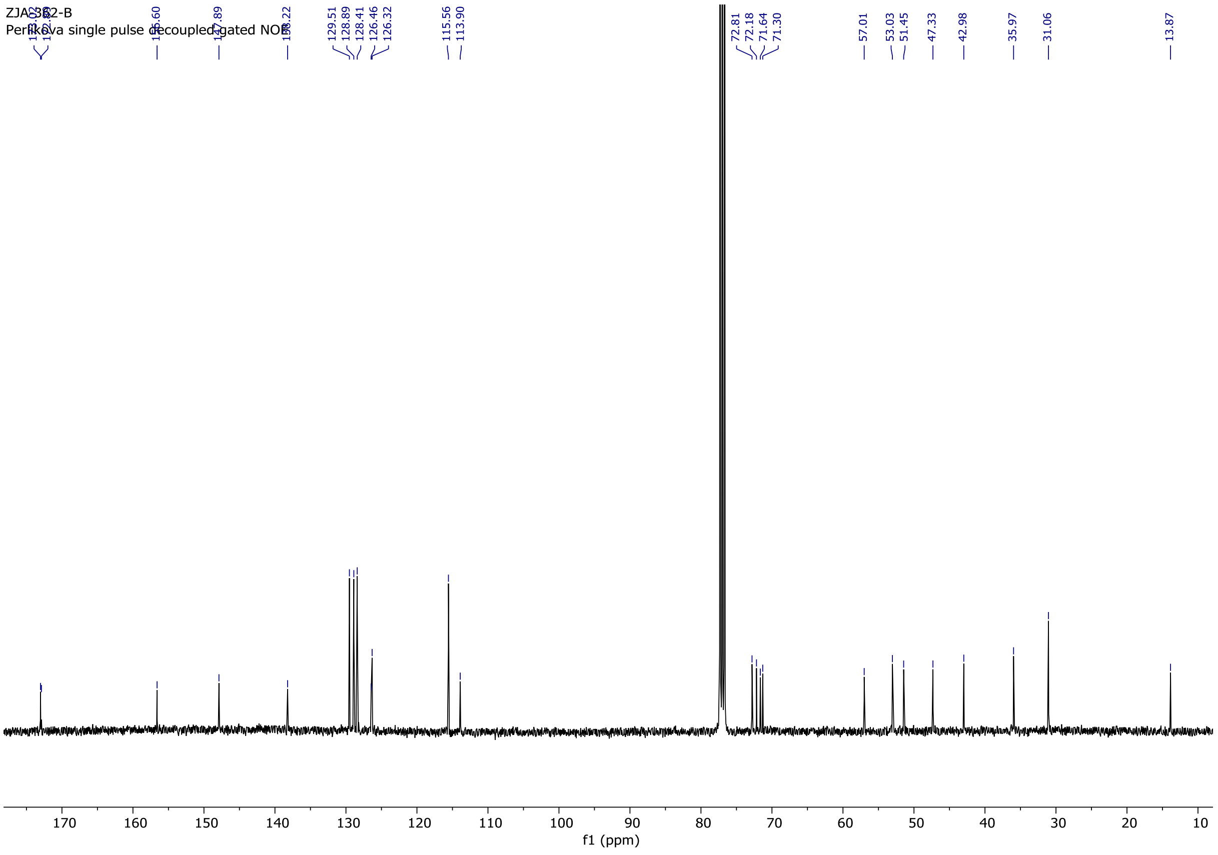

Compound 12g

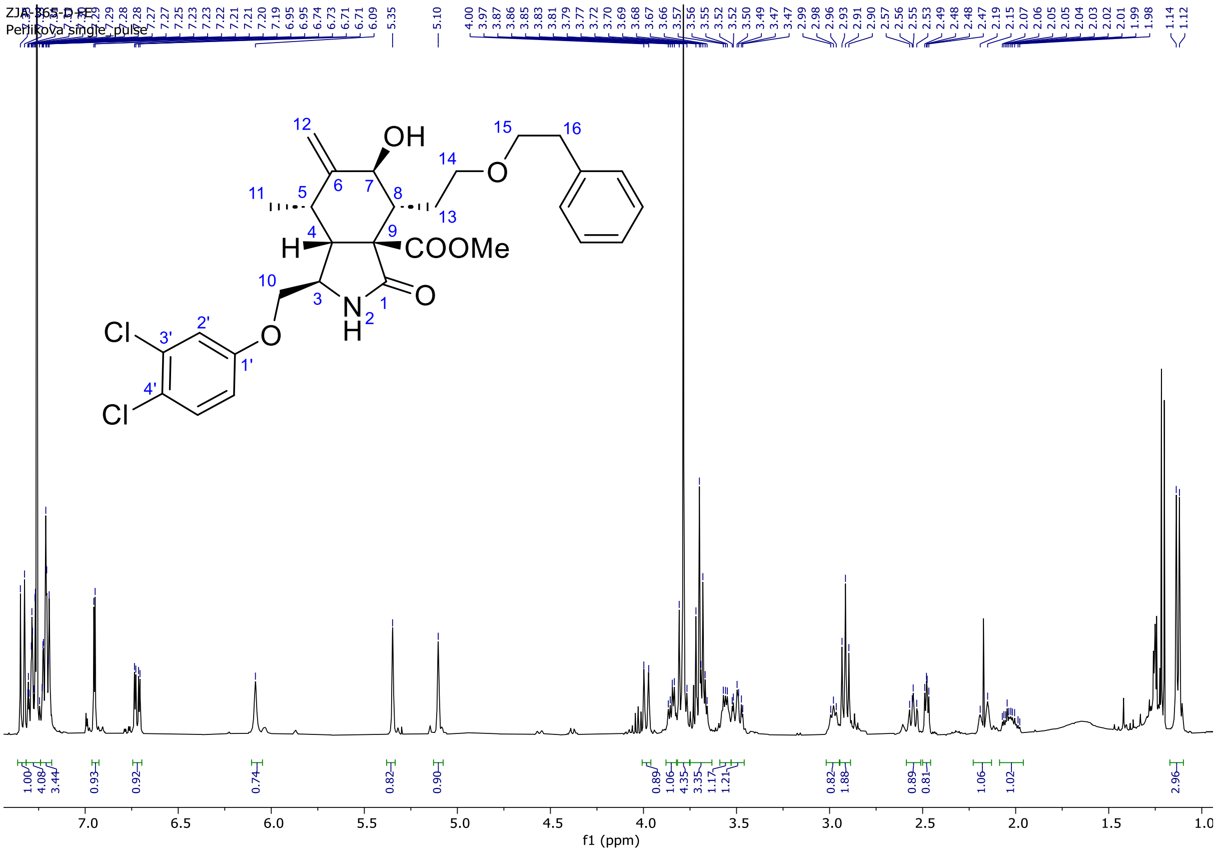

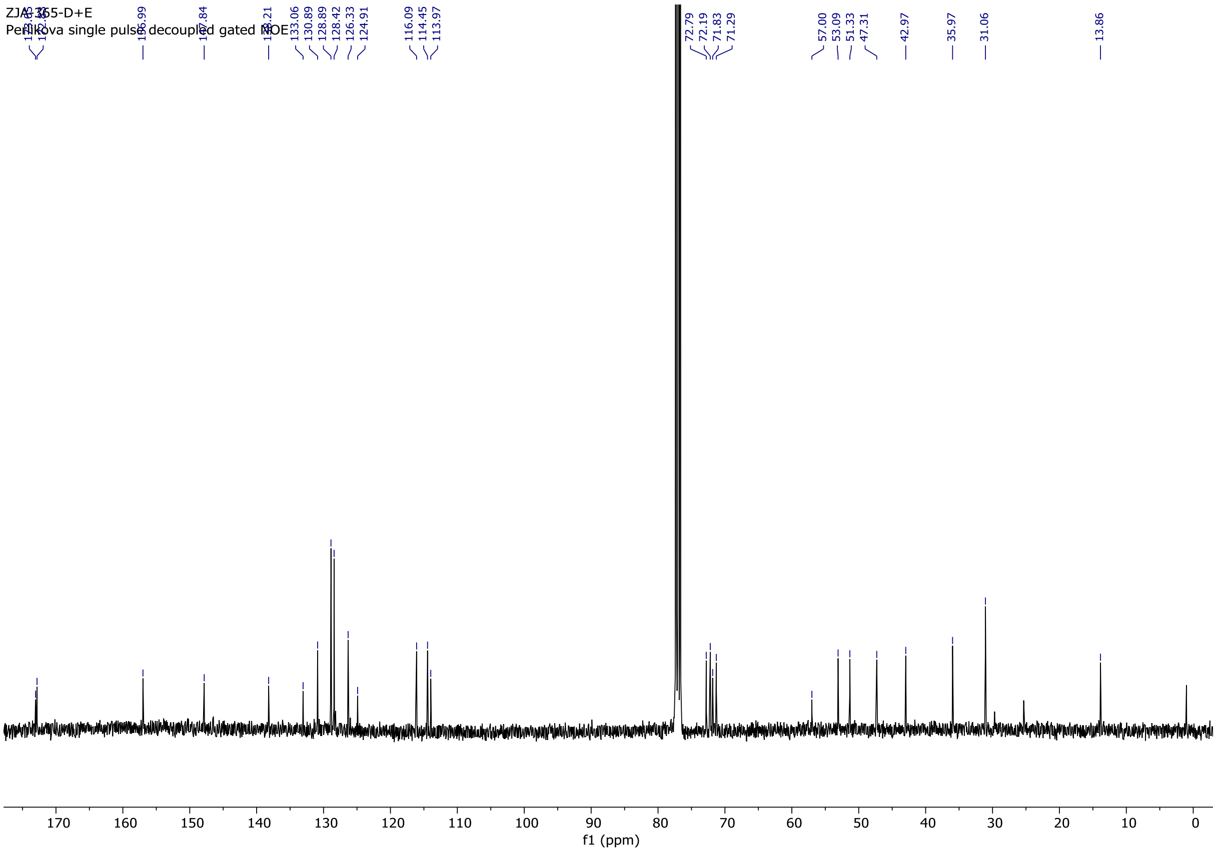

Compound S1

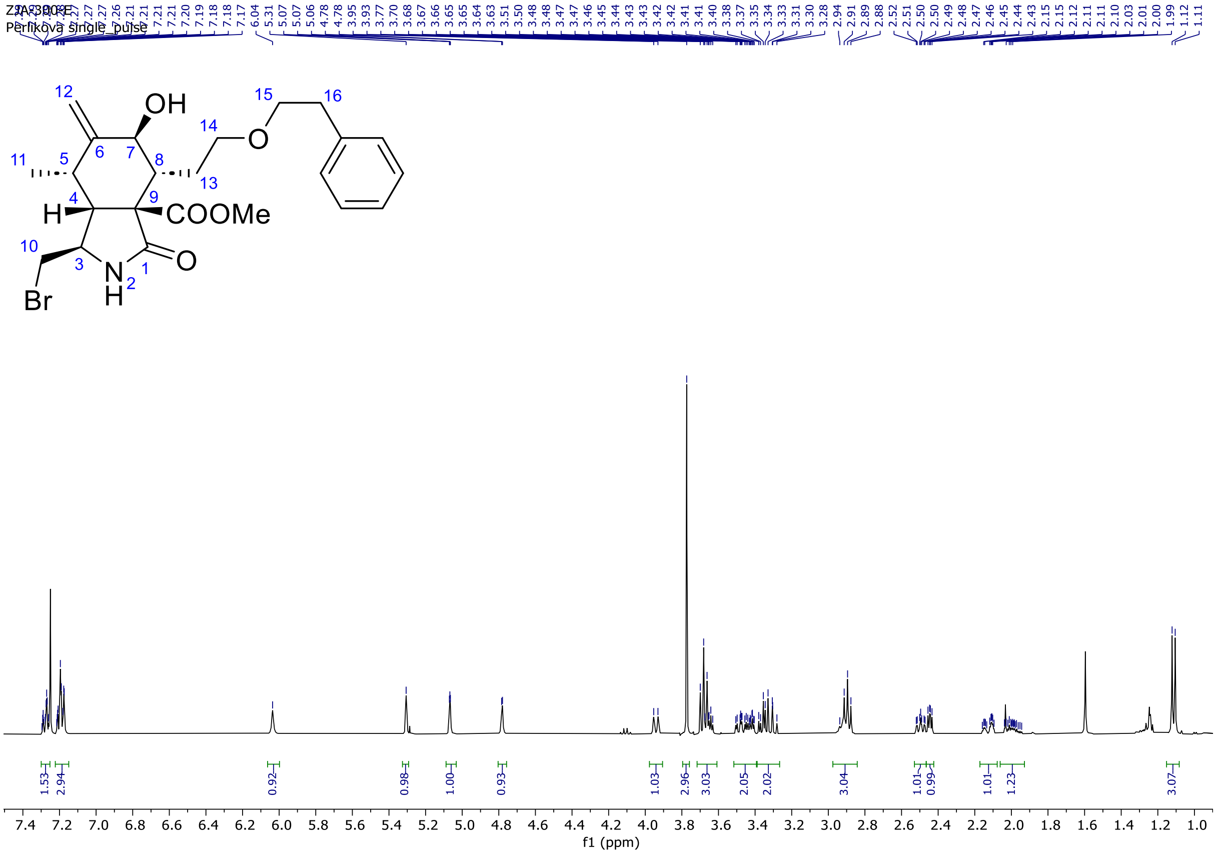
ZJA-300-E
Perlíková single_pulse
12
OH
15
16
14
O
11
5
6
7
8
13
4
9
H
COOMe
10
3
1
O
N
2
H
Br
f1 (ppm)

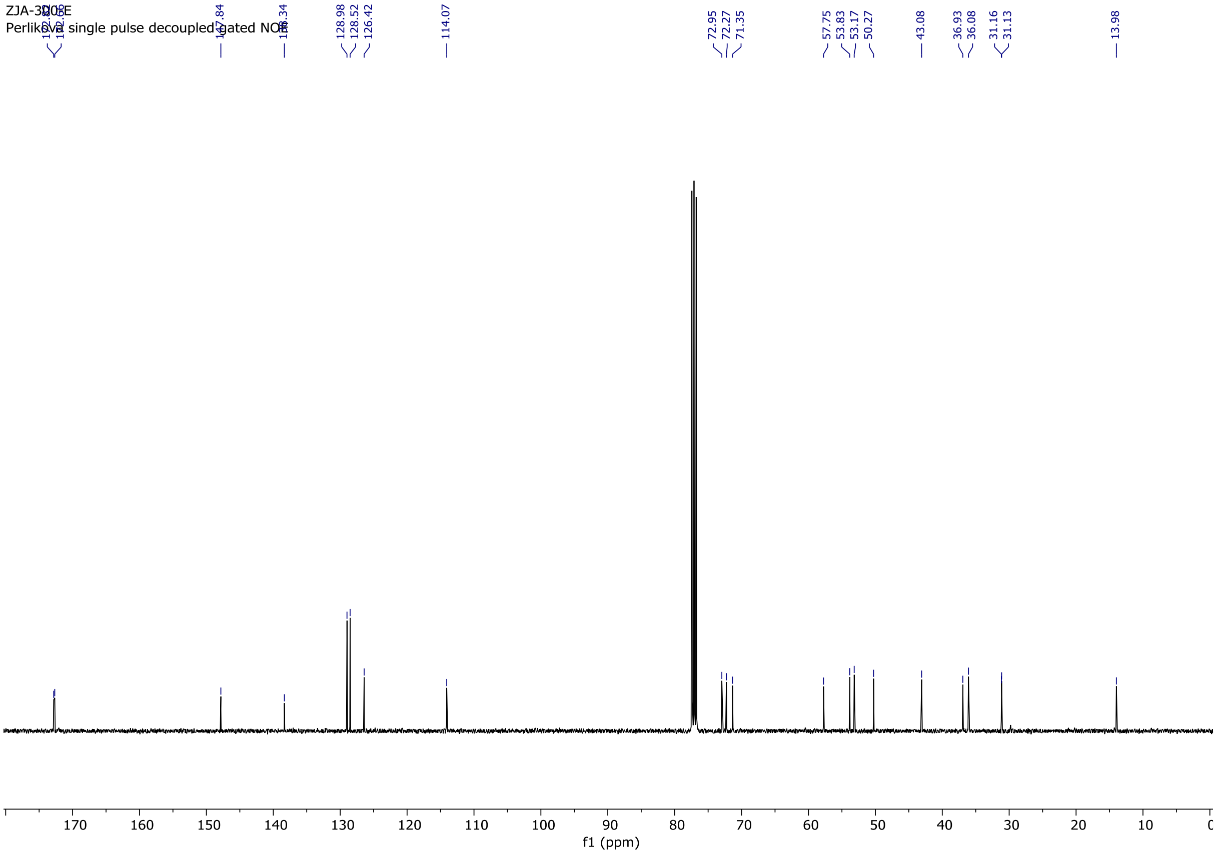
ZJA-300-E
Perlíková single pulse decoupled gated NOE
147.84
128.98
128.52
126.42
114.07
72.95
72.27
71.35
57.75
53.83
53.17
50.27
43.08
36.93
36.08
31.16
31.13
13.98
f1 (ppm)

Compound S2

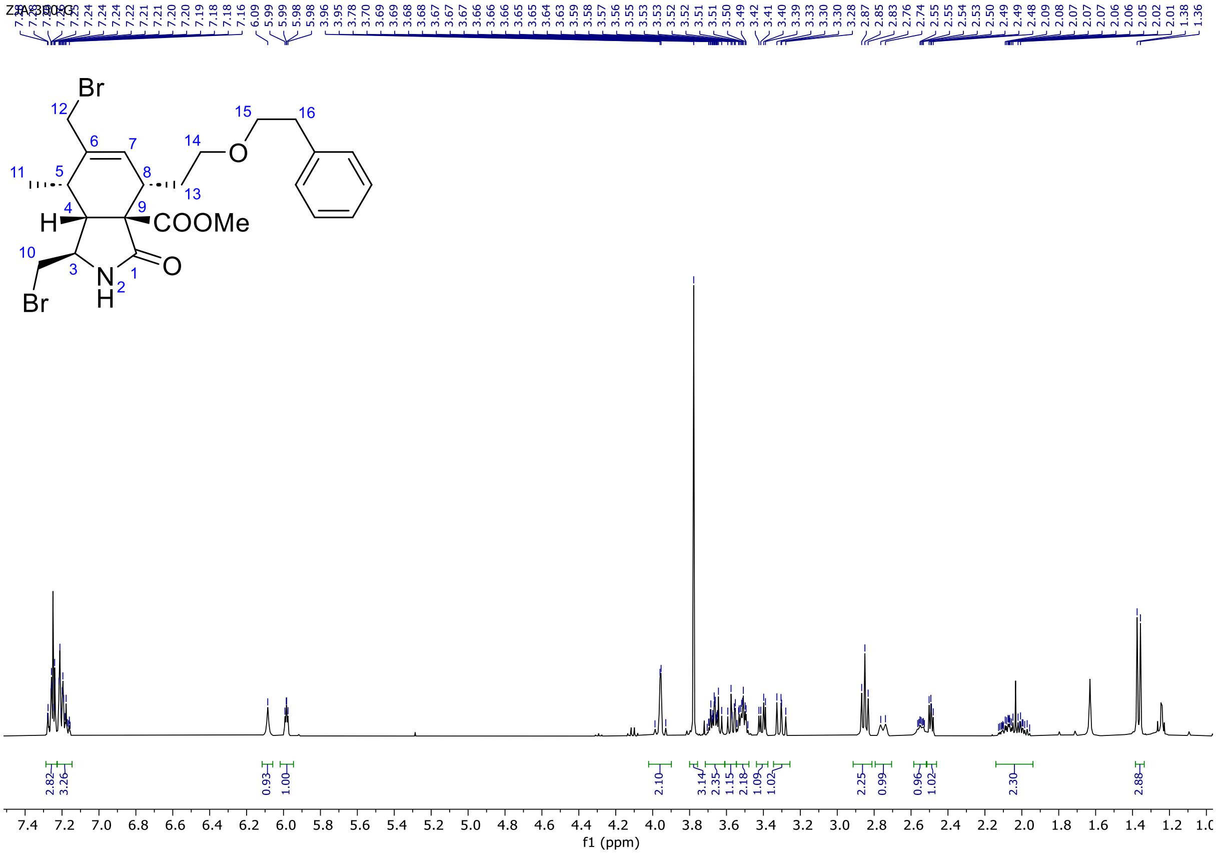
ZJA-308-G
Br
12
6
7
11
5
8
14
O
15
16
13
4
9
H
COOMe
10
3
1
N
2
O
H
Br
2.82
3.26
0.93
1.00
2.10
3.14
2.35
1.15
2.18
1.09
1.02
2.25
0.99
0.96
1.02
2.30
2.88
f1 (ppm)

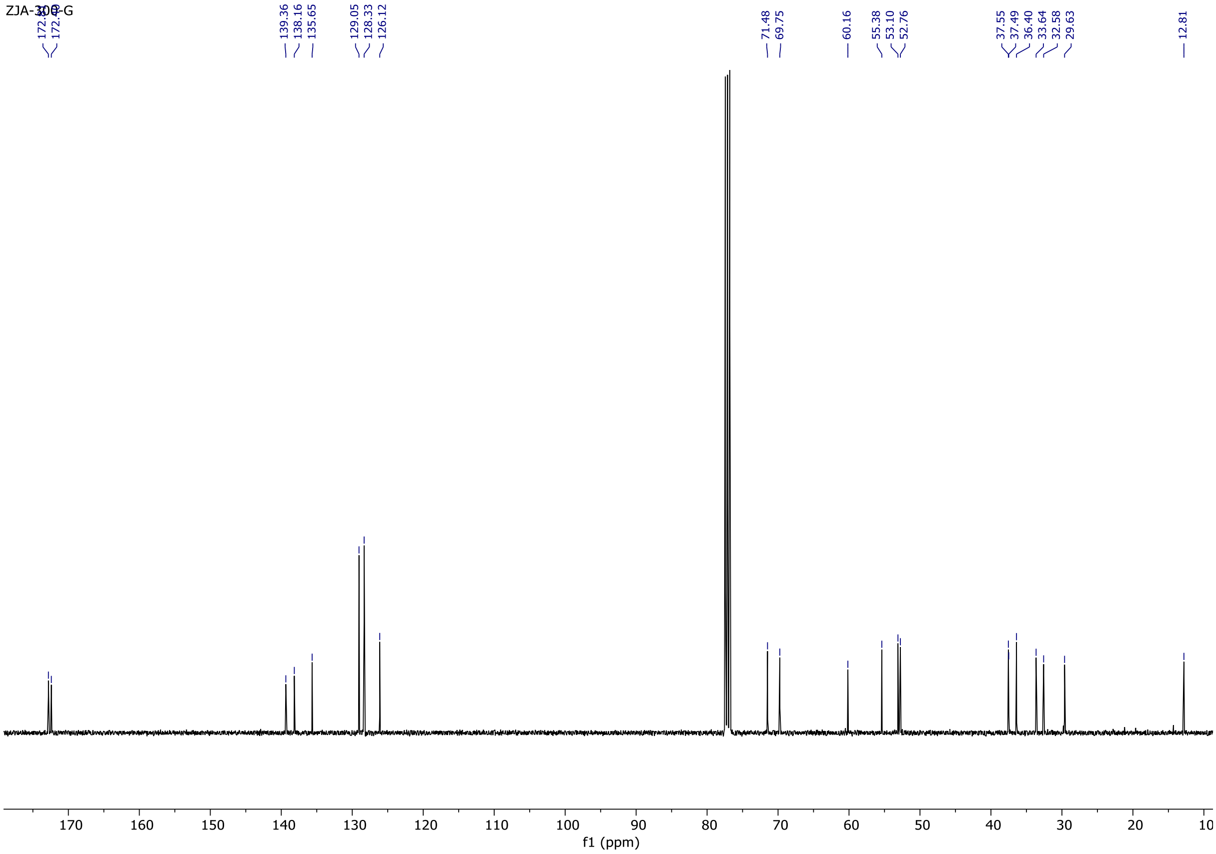
ZJA-308-G
139.36
138.16
135.65
129.05
128.33
126.12
71.48
69.75
60.16
55.38
53.10
52.76
37.55
37.49
36.40
33.64
32.58
29.63
12.81
f1 (ppm)

Compound S3

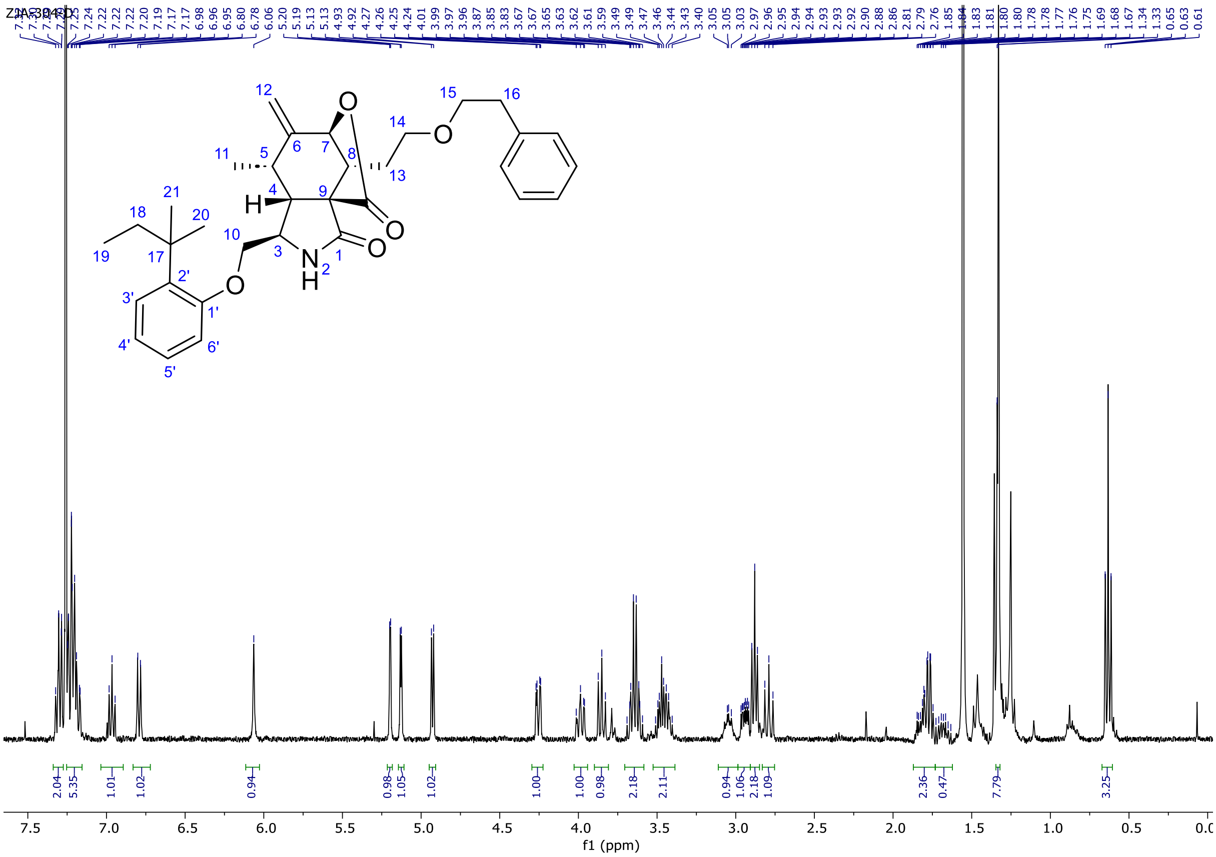

ZJA-304-D
7.25 7.24 7.22 7.22 7.22 7.20 7.19 7.17 7.17 6.98 6.96 6.95 6.80 6.78 6.06 5.20 5.19 5.13 5.13 4.93 4.92 4.27 4.26 4.25 4.24 4.01 3.99 3.97 3.96 3.87 3.85 3.83 3.67 3.65 3.63 3.62 3.61 3.59 3.49 3.49 3.47 3.46 3.44 3.43 3.40 3.05 3.05 3.03 2.97 2.96 2.95 2.94 2.93 2.93 2.92 2.90 2.88 2.86 2.81 2.79 2.76 1.85 1.83 1.81 1.80 1.80 1.78 1.77 1.76 1.75 1.69 1.68 1.67 1.34 1.33 0.65 0.63 0.61
12
O
15
16
14
O
11
5
6
7
8
13
21
H
4
9
18
20
O
10
19
17
3
N
1
O
2'
2
H
3'
O
1'
4'
6'
5'
2.04 5.35 1.01 1.02 0.94 0.98 1.05 1.02 1.00 1.00 0.98 2.18 2.11 0.94 1.06 2.18 1.09 2.36 0.47 7.79 3.25
7.5 7.0 6.5 6.0 5.5 5.0 4.5 4.0 3.5 3.0 2.5 2.0 1.5 1.0 0.5
f1 (ppm)

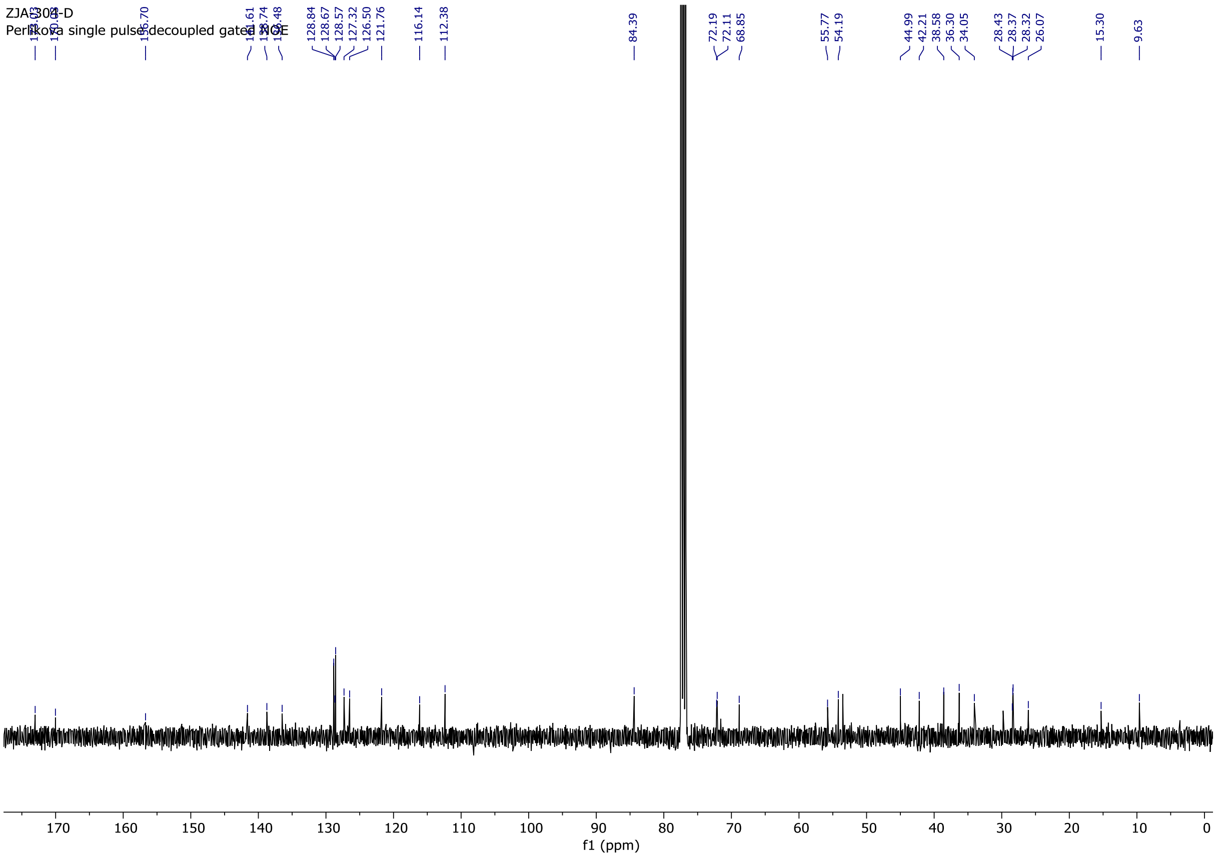

ZJA-304-D
Perlikova single pulse decoupled gated NOE
141.61 128.84 128.67 128.57 127.32 126.50 121.76 116.14 112.38 84.39 72.19 72.11 68.85 55.77 54.19 44.99 42.21 38.58 36.30 34.05 28.43 28.37 28.32 26.07 15.30 9.63
170 160 150 140 130 120 110 100 90 80 70 60 50 40 30 20 10 0
f1 (ppm)

Compound S4

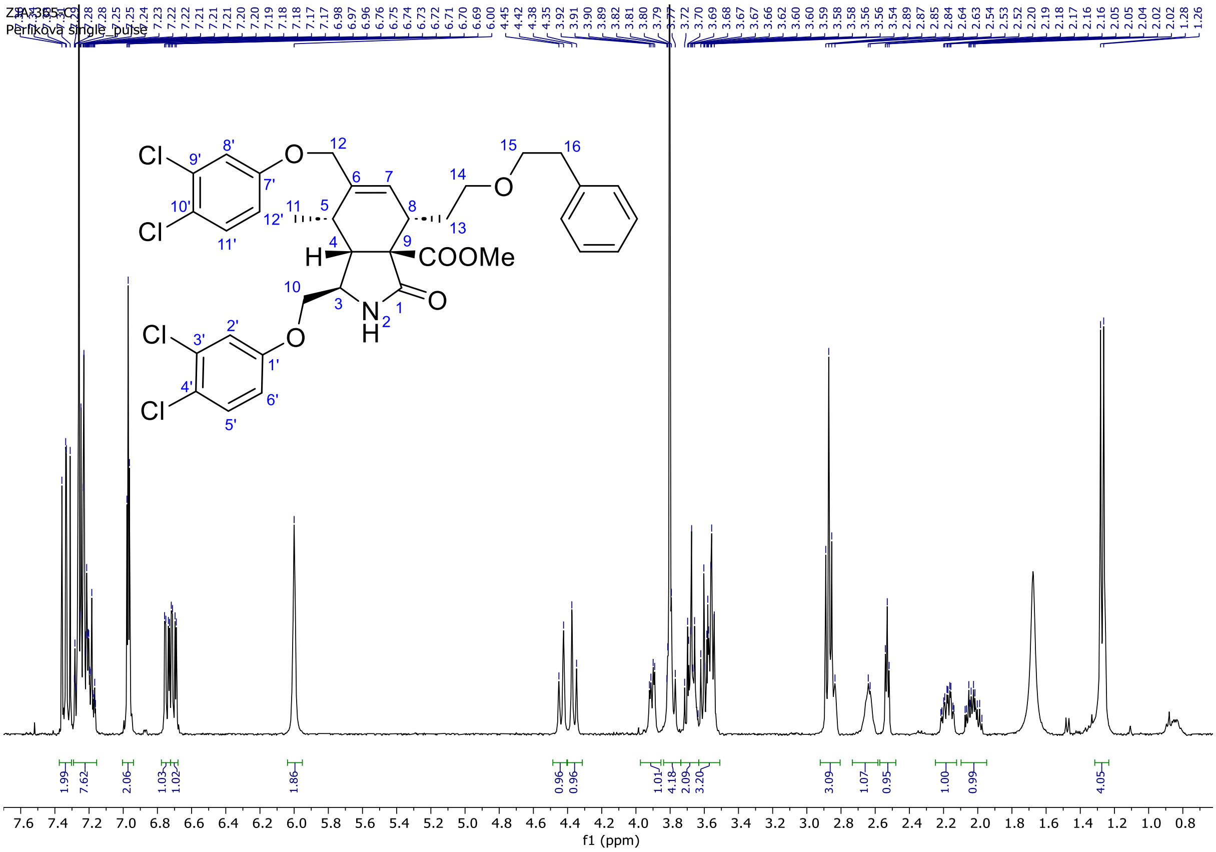
ZJA-365-C
Perlikova single_pulse
7.28 7.28 7.25 7.25 7.24 7.23 7.22 7.22 7.21 7.21 7.21 7.20 7.20 7.19 7.18 7.18 7.17 7.17 6.98 6.97 6.96 6.76 6.75 6.74 6.73 6.72 6.71 6.70 6.69 6.00 4.45 4.42 4.38 4.35 3.92 3.91 3.90 3.89 3.82 3.81 3.80 3.79 3.77 3.72 3.70 3.69 3.68 3.67 3.67 3.66 3.62 3.60 3.60 3.59 3.58 3.58 3.56 3.56 3.54 2.89 2.87 2.85 2.84 2.64 2.63 2.54 2.53 2.52 2.20 2.19 2.18 2.17 2.16 2.16 2.05 2.05 2.04 2.02 2.02 1.28 1.26
Cl
O
COOMe
H
N
H
1.99 7.62 2.06 1.03 1.02 1.86 0.96 0.96 1.01 4.18 2.09 3.20 3.09 1.07 0.95 1.00 0.99 4.05
7.6 7.4 7.2 7.0 6.8 6.6 6.4 6.2 6.0 5.8 5.6 5.4 5.2 5.0 4.8 4.6 4.4 4.2 4.0 3.8 3.6 3.4 3.2 3.0 2.8 2.6 2.4 2.2 2.0 1.8 1.6 1.4 1.2 1.0 0.8
f1 (ppm)

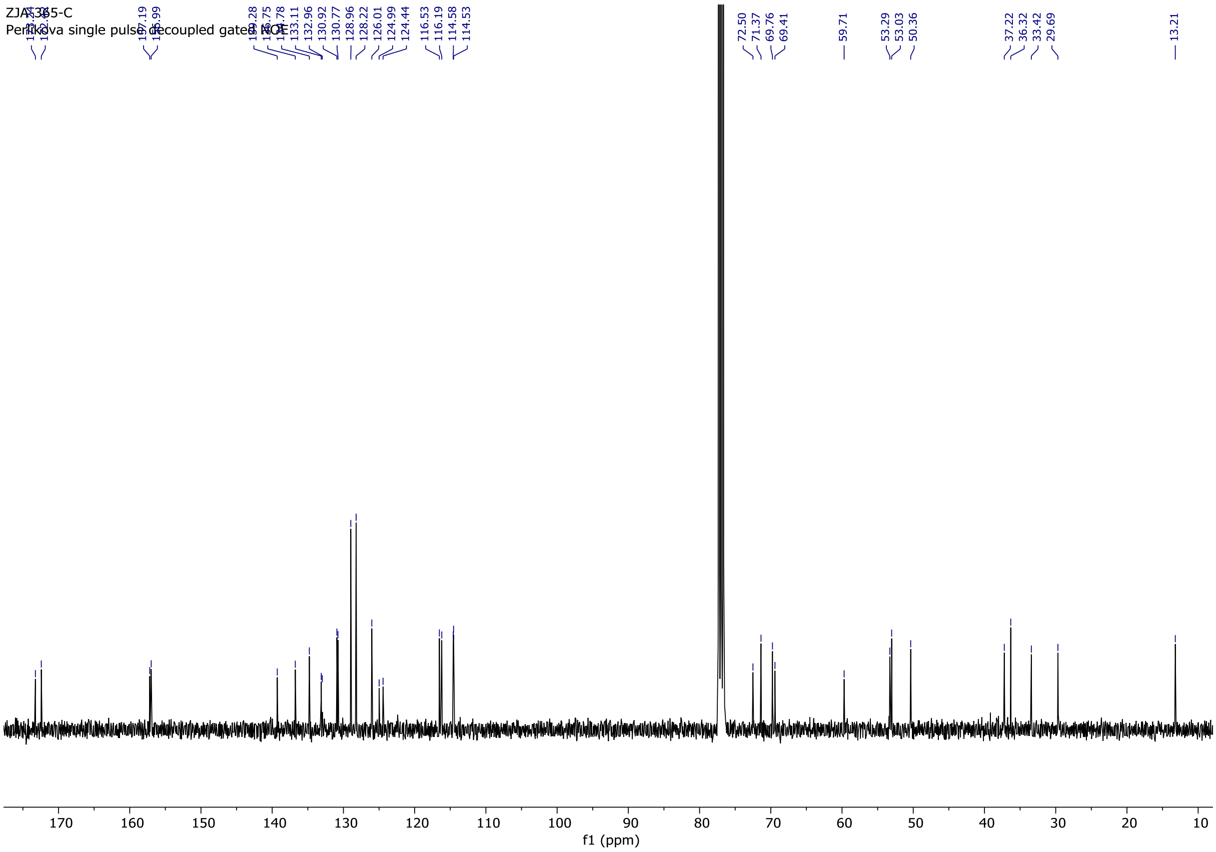
ZJA-365-C
Perlikova single pulse decoupled gated NOE
133.11 132.96 130.92 130.77 128.96 128.22 126.01 124.99 124.44 116.53 116.19 114.58 114.53
72.50 71.37 69.76 69.41
59.71
53.29 53.03 50.36
37.22 36.32 33.42 29.69
13.21
170 160 150 140 130 120 110 100 90 80 70 60 50 40 30 20 10
f1 (ppm)

References

(1) Nair, U. B.; Joel, P. B.; Wan, Q.; Lowey, S.; Rould, M. A.; Trybus, K. M. Crystal Structures of Monomeric Actin Bound to Cytochalasin D. *J. Mol. Biol.* **2008**, *384* (4), 848–864. https://doi.org/10.1016/j.jmb.2008.09.082.

(2) Javorská, Ž.; Rimpelová, S.; Labíková, M.; Perlíková, P. Synthesis of Cytochalasan Analogues with Aryl Substituents at Position 10. *Org. Biomol. Chem.* **2024**, *22* (22), 4536–4549. https://doi.org/10.1039/D4OB00634H.

(3) Yu, J.; Li, C.; Zeng, H. Dearomatization-Rearomatization Strategy for *Ortho* -Selective Alkylation of Phenols with Primary Alcohols. *Angew. Chem. Int. Ed.* **2021**, *60* (8), 4043–4048. https://doi.org/10.1002/anie.202010845.

(4) Binder, M.; Tamm, C.; Turner, W. B.; Minato, H. Nomenclature of a Class of Biologically Active Mould Metabolites: The Cytochalasins, Phomins, and Zygosporins. *J. Chem. Soc. Perkin 1* **1973**, 1146. https://doi.org/10.1039/p19730001146.
